# In Silico Analysis of Cross-Species Sequence Variability in Host Interferon Antiviral Pathway Proteins and SARS-CoV-2 Susceptibility

**DOI:** 10.15212/ZOONOSES-2024-0028

**Published:** 2024-11-27

**Authors:** Sally A. Mayasich, Peter G. Schumann, Maxwell Botz, Carlie A. LaLone

**Affiliations:** 1 Aquatic Sciences Center, University of Wisconsin-Madison, Madison, USA; 2 US Environmental Protection Agency, Office of Research and Development, Center for Computational Toxicology and Exposure, Great Lakes Toxicology and Ecology Division, Duluth, MN, USA; 3 Oak Ridge Institute for Science and Education, Duluth, MN, USA

**Keywords:** SARS-CoV-2, COVID-19, cross-species susceptibility, interferon, protein-protein interaction

## Abstract

**Background:**

Zoonotic transmission of severe acute respiratory coronavirus 2 (SARS-CoV-2) has been found to result in infections in more than 30 mammalian species. The SARS-CoV-2 spike protein binds to the host’s angiotensin converting enzyme 2 (ACE2) cell surface receptor to gain entry into the cell. ACE2 protein sequence conservation has therefore been evaluated across species, and species with amino acid substitutions in ACE2 were ranked low for susceptibility to SARS-CoV-2 infection. However, many of these species have become infected by the virus.

**Methods:**

This study investigated the conservation of 24 host protein targets, including the entry proteins ACE2 and transmembrane serine protease 2 (TMPRSS2); 21 proteins in the interferon-I (IFN-I) antiviral response pathway; and tethrin, a protein that suppresses new virion release from cells. Bioinformatics approaches including Sequence Alignment to Predict Across Species Susceptibility (SeqAPASS), Molecular Operating Environment (MOE), and iCn3D software were used to compare protein sequence similarity, conserved domains, and critical amino acids for host-viral protein-protein interactions. The types of bonding interactions were scored, and the results were compared with empirical data indicating which species have or have not become infected.

**Results:**

This pathway approach revealed that 1) 13 proteins were conserved, whereas five lacked data sufficient to determine specific critical amino acids; 2) variation in protein-protein interfaces is tolerated for many amino acid substitutions, and these substitutions follow taxonomic clades rather than correlating with empirically determined species infection status; and 3) four proteins (MDA5, NEMO, IRF3, and ISG15) contained potential domains or specific amino acids whose substitution may result in PPI disruption.

**Conclusion:**

This work provides evidence that certain substitutions in four IFN-I antiviral pathway proteins appear able to disrupt interactions and may be distinctive to resistant species, thus potentially aiding in determining species’ likelihood of transmitting SARS-CoV-2.

## INTRODUCTION

Many proteins in the innate immune system become involved in interactions with pathogens that enter host cells. Initiation of the interferon-I (IFN-I) antiviral response is necessary for expression of IFNs, which in turn promote the expression of interferon stimulated genes (ISGs) and thereby inhibit or limit viral replication. Early in infection, IFN-I is the main component of the innate immune system that is suppressed by the SARS-CoV-2 coronavirus [1,2]. Unchecked viral replication increases disease severity, host-to-host transmission, and the potential for mutations creating more virulent strains. Recent empirical investigations have shown that most studied mammals allow viral entry [3,4], whereas outcomes differ regarding the generation of viral loads, and intra- and interspecies transmission [5,6]. These findings have implicated factors in the antiviral pathway as important determinants of viral replication and the spread of infection, after viral entry into host cells.

In this study, 24 proteins were investigated to understand their conservation across species. These proteins included entry proteins; proteins in the IFN-I antiviral response pathway; and tethrin (bone marrow stromal antigen 2 [BST-2]), a last defense to restrict virion release from cells (Table 1, S1 Table). The objective of this study was to evaluate pathway conservation and whether amino acid substitutions in IFN-I pathway proteins might help predict species susceptibility to SARS-CoV-2 infection. Our hypothesis was that amino acid substitutions affect not only entry proteins but also IFN-I protein target interactions with viral stressors, thereby creating cross-species variation in susceptibility to SARS-CoV-2.

To gain entry to cells, the SARS-CoV-2 viral spike (S) protein binds the angiotensin converting enzyme 2 (ACE2) receptor; subsequently the S protein is primed by transmembrane serine protease 2 (TMPRSS2), which proteolytically cleaves and activates viral envelope glycoproteins, thus enabling membrane fusion [7]. ACE2 has been the sole subject of studies aimed at predicting alternate host susceptibility. In the first report of a SARS-CoV virus in 2003 [8], and reports of the original SARS-CoV-2 Wuhan strain [3,9–13] and its variants [14,15], researchers have compared ACE2 sequences across species, with respect to interacting amino acid residues. However, wild and domestic mammals have been reported to contract COVID-19 regardless of the wide range of substitutions in the ACE2-S interacting amino acids [4,16].

Extensive protein-protein interaction (PPI) networks have been demonstrated among SARS-CoV-2 viral RNA, non-structural proteins (NSPs), structural and accessory (open reading frame [ORF]) proteins, and host molecules [17]. The viral structural proteins include the S, envelope (E), membrane (M), and nucleocapsid (N) proteins. Viral-host PPIs in the IFN-I pathway (S1 Fig), as well as proteins involved in viral entry and “exit,” have been investigated through comparison of full protein sequences, conserved functional domains, and individual amino acid residues across species (Table 1). The criteria for the selection of the proteins investigated herein were 1) evidence of PPI between a SARS-CoV-2 protein and the host protein, and 2) experimentally demonstrated inhibition of IFN and ISG expression caused by the viral-host PPI.

New Approach Methods (NAMs) are being developed in toxicology to avoid the use of live animals in chemical safety evaluations [18]. These methods include in vitro and in silico methods that can be used to predict cross-species susceptibility to chemicals. In silico NAMs are well suited to cross-species evaluations of PPIs in pathogen infection. The US Environmental Protection Agency’s Sequence Alignment to Predict Across Species Susceptibility (SeqAPASS) tool (https://www.epa.gov/chemical-research/sequence-alignment-predict-across-species-susceptibility) [19] was applied to evaluate conservation of the PPIs between host and SARS-CoV-2 pathogen protein stressors across species. The SeqAPASS tool (Version 6.1) assesses protein sequence data across species, by comparing the full or primary amino acid sequence (Level 1), conserved portions of a sequence representing functional domain(s) (Level 2), and individual amino acid positions, to determine whether the critical contact residues are conserved (Level 3). Each level requires a more in-depth preliminary investigation of the protein to identify features and residues involved in interactions with a stressor. Empirical data found through literature searches, protein sequence and structural databases, and computational molecular models were used to identify available sequences and species known to have tested positive for SARS-CoV-2 RNA or antibodies (seroconversion). In addition, the literature was mined to identify conserved binding domains, and interacting amino acid residues between SARS-CoV-2 proteins and host protein targets. As empirically determined SARS-CoV-2 infections across many species of mammals continue to be reported [16], cell entry apparently occurs in these species.

Most viral protein mutations in the recent SARS-CoV-2 variants have not had their structures elucidated beyond the ACE2-S protein interaction. Therefore, this analysis included only evaluations involving the original Wuhan strain of SARS-CoV-2.

The pathway approach described herein offers a novel and comprehensive perspective on the crucial epidemiological concerns regarding various species’ propensity to allow viral production and transmission.

## MATERIALS AND METHODS

### Host protein target selection

To determine protein targets of SARS-CoV-2, the first criterion for protein selection was evidence of PPI between a SARS-CoV-2 protein and the host protein. These proteins included the entry proteins ACE2 and TMPRSS2, and the exit protein tethrin. Second, IFN-I proteins were the study focus because of their key roles in initial antiviral defense as a response pathway. Therefore, proteins in the canonical innate immune pathways involved in general responses to viruses—particularly those known to be involved in responses to coronaviruses including the first SARS-CoV—were considered in our literature review. The literature searches were conducted in the PubMed and Google Scholar databases. A wide variety of search terms were used in conjunction with “SARS-CoV-2” and “COVID-19” to identify information on specific protein interactions, such as “SARS-CoV-2 nsp3+IRF3” (with both acronyms and full terms used in search strings). This process identified proteins that are crucial components of the IFN-I pathway signaling cascade that have also been shown to interact with SARS-CoV-2 proteins (S1A and B Fig). Searches were then performed in the Research Collaboratory for Structural Bioinformatics (RCSB) Protein Data Bank (PDB) structure database (RCSB. org), National Center for Biotechnology Information (NCBI; ncbi.nlm.nih.gov/protein), and UniProt (uniprot. org) protein databanks, as well as the CDD conserved domain database (ncbi.nlm.nih.gov/cdd) [20,21], to find structural, functional, and conserved domain information for the identified host protein targets of SARS-CoV-2 proteins. Critical amino acids for binding were determined for targets for which experimental data—such as site-directed mutagenesis, solved structures, or structural modeling—were available. The 24 proteins meeting these criteria are listed in Table 1.

### Determination of critical amino acids for protein-protein interaction

Critical amino acids for host-viral PPI were determined from the literature for several targets for which experiments such as truncation (MAVS) or cleavage (IRF3, NEMO) had been performed (Table 1, S1 Table). In some cases, e.g., IRF3, structures had previously been determined for PPI between IRF3 and its adaptor proteins STING, MAVS, and TRIF, which bind at the same location as rotavirus non-structural protein 1 (NSP1). The binding locations of these normal host-host PPIs were used to infer potential binding locations for SARS-CoV-2 proteins with the host target.

Structural modeling had previously been conducted for TBK1 [22] and BST-2 [23] to propose interacting residues, through modeling strategies explained in those publications. The likely pocket residues for BST-2 were confirmed with the Molecular Operating Environment (MOE) software (CCG, Montreal, CA) Site Finder tool, using the unbound crystal structure PDB 3NWH.

For targets for which the PPI had been structurally determined, e.g., by X-ray diffraction or cryo-EM, the interacting amino acids were identified using iCn3D (https://www.ncbi.nlm.nih.gov/Structure/icn3d/icn3d.html) [24], as well as the MOE software contacts tool (default settings), to view inter-chain interactions. Both iCn3D and MOE identify the contact type as “bond” (hydrogen, metal, ionic, arene, or covalent) or “distance” (e.g., Van der Waals forces). The energy (Gibbs free energy) of the interaction is given in kcal/mol, and the distance of contact is given in angstroms (Å) with a default cut-off for MOE of < 4.5 Å and iCn3D set to 5 Å. Interactions were compared for each structure, and across any additional structures for which more than one structure was available per target in the RCSB PDB. Additionally, any contacts specified by the researchers who published the structure were also compared. All amino acids forming bonds, as indicated by any method, were selected as critical bond contact amino acids. Distance contact amino acids were selected when agreement was found among most methods and/or the MOE contact data indicated that the host residue contacted two or more residues in the viral protein. Distance contacts without agreement among the majority of methods and with single contact residues were selected only if either the distance was < 3.8 Å or the energy was < −0.5 kcal/mol. Contacts not meeting these criteria were not included in the SeqAPASS Level 3 analyses.

### SeqAPASS evaluations and clustered heatmaps

SeqAPASS queries for each protein were run according to the SeqAPASS User Guide (Version 6.1; epa.gov/chemical-research/seqapass-user-guide), based on information available for each selected host protein. If no information was available on specific residues, only SeqAPASS Level 1 was performed, and Level 2 was performed if specific hits in the CDD corresponded to protein binding locations. For the proteins for which critical amino acids for binding were also determined, SeqAPASS Levels 1, 2, and 3 were performed. Briefly, a query protein sequence or NCBI accession number was used to begin a query. Herein, the human protein was used as the query species for all SeqAPASS jobs. The SeqAPASS algorithms use information from the NCBI protein database, conserved domains database, and taxonomy database, and align sequences with the Stand-Alone Basic Local Alignment Search Tool for proteins (BLASTp) and the Constraint-based Multiple Alignment Tool (COBALT). We did not use the susceptibility cut-off and ortholog candidate predictions; instead, our focus was on the percentage similarity to the query (human) sequence. SeqAPASS calculates the percent similarity by dividing the best hit BLAST bitscore for each hit species by the query species BLAST bitscore, and multiplying by 100 [19].

Normally, SeqAPASS Level 3 evaluations yield a final “yes” or “no” susceptibility call based on the conservation of individual, critical amino acids. However, each PPI within the SARS-CoV-2 infection pathway is mediated by multiple amino acid residue contacts. Therefore, to account for each of these interacting amino acids, we converted the SeqAPASS Level 3 evaluation results through an external analysis not connected to the SeqAPASS tool into a similarity score with a custom R (v4.1.3) function. Within SeqAPASS Level 3, amino acid conservation is estimated according to three parameters: residue identity, side chain classification, and molecular weight, which are given either a “yes” or “no” result based on comparison with a reference protein sequence [19]. Of note, a match for molecular weight is an amino acid with a difference of ≤ 30 g/mol with respect to the template amino acid (as described in the SeqAPASS User Guide). The similarity scoring function summed all “yes” calls for each interacting amino acid across these three parameters, and this count was doubled to weight the stronger bonds (for amino acids involved in hydrogen, arene, or ionic bonding). The summed value of the bond and distance residues was then normalized through conversion into a percentage of the total possible score (i.e., the score if all parameters were “yes” for each amino acid), thus yielding the “percentage similarity score.” This procedure was performed for each set of bond and distance interacting amino acids in each selected host protein in the study with crystal or cryo-electron microscopy (cryoEM) structures (Table 1), across all species. Empirical data capturing infection, replication, and transmission from the literature were compared with relevant protein sequence data.

Heatmap generation and hierarchical clustering were performed with percentage similarity data generated through SeqAPASS for Levels 1 and 2, and the above-described similarity scores for Level 3, after data curation. The R package “ComplexHeatmap” [25] was used, wherein dendrogram clusters were determined through Euclidean distance calculations. Sidebar annotations of viral RNA, seroconversion, and transmission were added according to results from previous studies.

## RESULTS

### Critical contact amino acids in host target proteins

The amino acids in the host proteins involved in PPI or in post-translational modifications were determined, because substitution at these positions in other species might cause disruption of the PPI, potentially resulting in differences in SARS-CoV-2 infection and transmissibility. Of the 24 proteins evaluated, 13 had, at the time of the study, been empirically elucidated with physiochemical methods including X-ray crystallography or cryoEM, and some viral-host protein complexes had more than one structure available in the RCSB PDB (S1 Table). All structures were of high quality, with resolutions below 3 Å, except for the horseshoe bat ACE2-S (PDB 7XA7), which had a resolution of 3.3 Å. Several lines of evidence of PPI were gathered through structural analyses in MOE software and iCn3D web-based structure viewer (S2 and S3 Tables). Both the MOE and iCn3D structural analyses indicated the types of binding interactions as hydrogen, covalent, ionic, or arene. Most bonds were hydrogen, some ionic, and arene or pi-stacking bonds also occurred for residues with aromatic ring structures, such as in phenylalanine. For this study, all of these were considered together as “bond” interactions. Interactions within a default cutoff of 4.5 Å (e.g., potential van der Waals forces) were designated as “distance” interactions by MOE, whereas a cutoff setting of 5 Å was used in iCn3D. The following lines of evidence were compared: interactions identified by 1) MOE and 2) iCn3D; 3) interactions determined by researchers who empirically solved the structures; and 4) interactions across different solved structures for the same complexed proteins. The results for these lines of evidence were similar but not in complete agreement (S2 and S3 Tables). A similar number of bond and distance interactions was identified for each of these structures (Table 1), with an average of 8.6 bond and 9.6 distance residues per protein. This finding was somewhat skewed by TOMM70, which had 13 bond and 33 distance residues. Of the 13 host proteins with solved structures, nine of the bound structures were host protein and SARS-CoV-2 protein complexes (40S ribosomal subunits uS3 and uS5-NSP1, ACE2-S, G3BP1-N, ISG15-NSP3, NEMO-NSP5, POLA1-NSP1, RAE1-Orf6, and TOMM70-Orf9b). In the four other structures, the host target protein was complexed with activating proteins (such as IRF3 with STING, MAVS, and TRIF; and MAVS with IRF3 and TRAF6), proteins from other viruses (IRF3 bound to rotavirus NSP1; STAT1 bound to Vaccinia [pox] virus protein 018), or an inhibitor bound to the active site (TMPRSS2 complex with nafamostat). These findings were considered to plausibly indicate that these sites are binding regions for the SARS-CoV-2 proteins. Additional post-translational modification sites were studied for ACE2 (N-glycosylation sites) [9], IRF3 (Plpro cleavage site) [26], and MDA5 and STAT1 and 2 (phosphorylation sites). Predicted binding residues from the literature were investigated for five additional proteins without bound crystal or cryoEM structures: BST-2, GIGYF2, MDA5, STAT2, and TBK1 (Table 1).

For ACE2, two human [27,28] and seven other mammalian ACE2-receptor-binding domain (RBD) structures (original Wuhan strain) have been solved. These additional mammals include the dog [29], cat [12], sea lion [30], pangolin [31], intermediate horseshoe bat [32], horse [33], and minke whale [30] (Table 2). We used MOE to investigate contacts in the mammalian structures, and identified 22 amino acids showing bond or distance interactions in most species (S4 Table). Highly conserved positions included S19, E35, Y83, K353, and D355; K31 and Q42 showed substitutions in only bats (asparagine) and minke whales (arginine), respectively. Most species had substitutions at Q24, D30, H34, D38, and M82. Bonding occurred in all species at K353, and at D38 regardless of D38E substitutions in all species but the minke whale. At all other positions, either bond or distance interactions were observed for the identical residues in different species. At least one amino acid in the RBD was paired in common in all species at each ACE2 binding position, regardless of the identity of the amino acid in the ACE2 position for that species, except at E35 and M82. The most interactions that occurred in common were G496, N501, G502, and Y505 in the S protein RBD with the conserved K353 in ACE2 in all host species. In variable positions, most substitutions were a partial match, but at H34, sea lions, pangolins, and horses have serine, and at M82, dogs, cats, horses, sea lions, and minke whales have threonine non-conservative substitutions (Table 2).

### Empirically determined species infections, seroconversion, and transmission

Since the start of the COVID-19 pandemic in late 2019, efforts have been made to identify potential intermediate or reservoir hosts. Bats are generally accepted to be a reservoir species [7]. Although speculation regarding the origin of the COVID-19 pandemic is ongoing, many mammals have been found to be susceptible and to act as intermediate hosts. Reports including confirmed natural infections as well as experimentally exposed animals with outcomes of either resistance, or infection with or without symptoms and transmission, have been compiled by Nielsen et al. (2023) and Nerpel et al. (2022) [4,16]. From this information and further investigation of the primary literature, we generated a list of species of interest (with available data on exposure outcomes) (Table 3). Species determined to transmit the virus in addition to primates include the prairie deer mouse (*Peromyscus maniculatus bairdii*), golden hamster, white-tailed deer, raccoon dog, red fox, domestic cat, domestic ferret, American mink, and Egyptian rousette bat. Species in which oral or nasal viral shedding was found but direct transmission was not investigated, include the woodrat, skunk [34], and red fox [35]. Notable species not found to transmit the virus include the house mouse (*Mus musculus*), pig, domestic dog, cow, and raccoon. Some recent data, with possible caveats, are as follows. The house mouse resists viral entry of the original Wuhan strain [36]. Of 39 Norway rats sampled in the sewer system in Antwerp, Belgium, none had SARS-CoV-2 RNA or antibodies while the original virus was circulating [37]. More than 600 racehorses in California were tested through 2020 for viral presence in nasal secretions (qPCR) and serum antibodies (ELISA), and of these, 0% positive qPCR tests and 5.9% positive tests for serum antibodies to SARS-CoV-2 were reported [38]. Seroconversion was not detected for big brown bats, and was inconsistently observed among pigs and ferrets (Table 3).

### SeqAPASS cross-species comparisons

**FIGURE 1 |:**
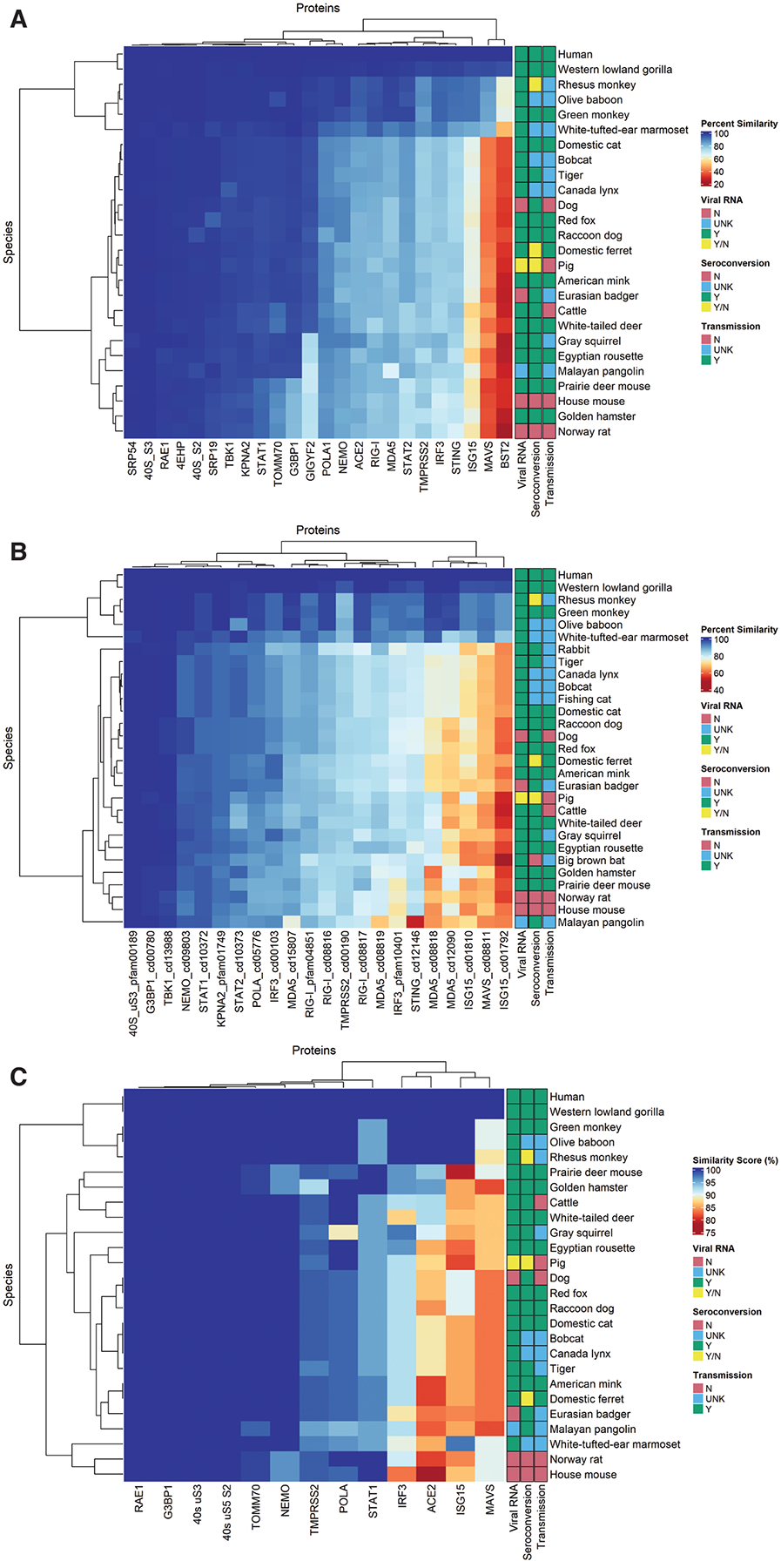
Clustered heatmaps for SeqAPASS results, based on sequence similarity (%) for Level 1 primary sequence (A), Level 2 conserved domain sequence (B), and Level 3 similarity score (C). Right sidebars indicate viral RNA detection, seroconversion, and transmission of SARS-CoV-2 infection according to literature data (see text and Table 3). Green (Y) indicates a positive test, red (N) indicates that the parameter was not detected, yellow (Y/N) indicates equivocal results, and blue (UNK) indicates unknown status (the parameter was not tested or investigated). The dendrogram at the left shows hierarchical clustering of species, whereas the dendrogram across the top shows the hierarchical relationships of the proteins, according to the level of sequence conservation.

Primary amino acid sequences for the 24 investigated proteins were aligned in Level 1 of SeqAPASS, through comparison of all species with sequences in the NCBI database (S1 File). Twenty-six mammalian species with empirically determined SARS-CoV-2 exposure outcomes had primary amino acid sequences for the 24 proteins. Conserved domains were not found in the literature or the NCBI CDD for 10 of the 24 proteins. Conserved domains were obtained for the potential binding regions of 14 proteins, some of which contained more than one conserved domain, for a total of 22 (S2 File). Twenty-nine species had available sequences for these 14 proteins. Most of the conserved domains were specific hits in the CDD; however, some were non-specific but had high e-values and were specific to the regions encompassing interacting amino acids, as indicated in the literature and determined through molecular structural analyses (Table 1). Critical amino acid data used in SeqAPASS Level 3 were obtained for the species of interest for 18 of the 24 proteins, for which 26 species had sequences (S3 File). The SeqAPASS Level 1, 2, and 3 results (compiled in S2–S25 Figs) indicated that sequence similarity was markedly lower in the non-mammalian than in the mammalian vertebrates, except for the conserved 40S ribosomal subunits and the signal recognition protein SRP54; moreover, 4EHP, RAE1, and TBK1 were also well conserved. The SeqAPASS results were curated to replace partial with complete sequences, or predicted with empirically determined sequences, when available (S4 File). Clustered heat maps were generated from the curated data to compare the sequence similarity to the human query sequence for Level 1 (Fig 1A) and Level 2 (Fig 1B), and a similarity score (Methods Section 2.3.1) was determined for the 13 proteins with available structures for Level 3 (Fig 1C) among species with published COVID-19 susceptibility information. A cluster of 11 proteins in Level 1 were conserved across species, but showed visually apparent variations in percentage similarity in the rodent compared to the human query sequences for STAT1, TOMM70, and G3BP1 (Fig 1A). The two least conserved proteins were MAVS and BST2. The Level 1 scores ranged from approximately 20% similarity to the human query sequence for BST2 in several species including the Norway rat, to 100% similarity for all species’ 40S_S3 sequences. The species clustered according to taxonomic clades, as might be expected for full sequences (Fig 1A).

Conserved domains are regions of interest likely to contain binding sites in the target protein. The sequence lengths in the hit proteins ranged from 74 (ISG15 cd01102) to 316 amino acid residues (TBK1 cd13988) (Table 1, S2 File). The domain encompasses the contact residues that had been determined from the respective crystal structures for the 40S ribosomal subunit uS3 pfam0189, G3BP1 cd00780, IRF3 pfam10401, ISG15 cd01792 and cd01810, MDA5 cd08818 and cd08819, POLA1 cd05776, STAT1 cd10372, and TMPRSS2 cd00190. In cases with no viral protein-bound structures, domains were selected based on specific hits in the CDD in protein-protein binding domains. These proteins included KPNA2, MAVS, MDA5, NEMO (cd09803 for potential binding with Orf9b), RIG-I, STAT2, and STING. The TBK1 cd13988 encompassed the in silico predicted binding residues (Table 1). The most variable cluster among the 22 domains included ISG15 cd01792 and cd01810, MAVS cd08811, and MDA5 cd12090 (Fig 1B). The most conserved cluster mirrored that in Level 1 (KPNA2, STAT1, NEMO, 40S subunit uS3, TBK1, and G3BP1). As in Level 1, the most variable to most conserved proteins across species tended to be ordered from immune-specialized proteins to general cellular maintenance proteins. The conserved domain sequences also showed relatively high sequence similarity, with scores ranging from approximately 40% to 100% similarity to the human sequences. Nonetheless, taxonomic clades were strongly clustered, as observed for the hominids and other primates; the felid, canid, and mustelid families within the carnivores; the artiodactyls; and the rodents. Of note, the Malayan pangolin was included as a species of interest; however, the MDA5 and STING sequences are currently annotated in the NCBI database as low-quality proteins (S2 File).

Furthermore, narrowing the number of residues to the specific amino acids involved in PPI in the crystal structures, the Level 3 clustered heatmap had a score range of approximately 75–100% similarity, and also clustered the immune-specialized MAVS, ISG15, and IRF3 together with ACE2 (Fig 1C). Differences in these proteins were the main factor dividing the cricetid and murid rodents. However, taxonomic cohesiveness was maintained by each group to different degrees for each of these proteins. Of note, differences in amino acid identity in the contact residues in each protein were found between the human and certain species, but these differences did not coincide with the known measures of infection, seroconversion, and/or transmission for those species. That is, on the basis of the average residue contacts per protein at the positions interacting with the viral protein stressors, no single protein, including ACE2, accounted for a notable proportion of positive viral RNA or seroconversion tests.

## DISCUSSION

The multi-level SeqAPASS alignments (Fig 1) aided in identifying key amino acid substitutions that might alter the PPIs in specific proteins of resistant species, as compared with sequences in humans and potential intermediate host species. The evaluation also identified data gaps for several proteins in the antiviral response pathway.

ACE2, the receptor that binds the viral S protein RBD enabling SARS-CoV-2 entry into host cells, has been the sole focus of prior cross-species susceptibility studies [3,9–11]. Damas et al. [9] ranked sequences of more than 400 vertebrate species to predict their susceptibility to SARS-CoV-2 host cell entry, by scoring the number and type of differences in the 22 interacting amino acids in the human SARS-CoV and SARS-CoV-2 crystal structures. The researchers focused on the binding sites K31, E35, M82, and K353, and the glycosylation sites N53, N90, and N322, based on the effects of amino acid substitution on binding of SARS-CoV S protein [8]. Luan et al. [11] compared binding contacts across the sequences of 42 mammals, with a focus on the five residues K31, E35, D38, M82, and K353, and have also conducted homology modeling of the interaction interfaces between cat, dog, pangolin, or Chinese hamster ACE2 and the SARS-CoV-2 RBD. Luan et al. [10] also modeled bovine and cricetid ACE2 structures and reported permissiveness to entry, whereas reptiles and birds showed changes in nearly half the interacting amino acid residues. At that time, few non-human species had been tested, and little empirical data on infections in other species existed. Susceptibility data are now available for more than 40 species [4] (Table 3).

Additionally, at the time only human ACE2-SARS-CoV-2 RBD crystal structures had been solved. More recently, several crystal or cryo-EM structures have been solved for other mammals, including the dog, cat, sea lion, pangolin, intermediate horseshoe bat, horse, and minke whale (Table 2). These ACE2-RBD structures represent several species beyond the human and house mouse model structures typically available for other bound proteins, presenting a rare opportunity for cross-species comparison of contact amino acids. Analysis of the contacts showed that the position, and not the identity, of the amino acid in the ACE2 dictates the amino acid contact pairing in the RBD. For example, a glutamine occupies position 24 in humans, whereas leucine, glutamic acid, or arginine occupies position 24 in the other species, but each remains paired with A475, G476, and N487 in the S protein RBD. This finding was observed at most positions, regardless of whether the ACE2 amino acid in each species was a match, partial match, or not a match to that in the human ACE2, according to the SeqAPASS results (Table 2). Despite viral binding regardless of amino acid differences at the contact positions, most of these species (dog, cat, pangolin, and horse) have been found to allow viral entry (Table 3), whereas the sea lion and minke whale have not been tested. The greatest differences in ACE2-RBD contact residue pairs with respect to those in humans were observed in horseshoe bats and cats. The low binding affinity (high K_d_) determined for the horseshoe bat provides an additional indication of the improbable binding of the bat ACE2 to the RBD. The horse had the lowest K_d_, which was most similar to the human K_d_ [33], but has shown little propensity for disease and no transmission [38] (Table 2). Beyond binding information, N-glycosylation site comparisons showed that many species had substitutions at either N90 or N322, but only mice had substitutions at both positions (S5B Fig). Although the amenability of the ACE2 to binding the SARS-CoV-2 S protein has demonstrated that correlations with infection and the positions of contact amino acids are important, their identity may be less important than previously thought, and more attention should be paid to post-translational modification sites. Additionally, cell entry assays conducted by Conceicao et al. [3], along with contact data from the solved mammalian structures (Table 2), suggest that, although cell entry is permitted, other mechanisms within cells may provide resistance to viral replication and transmission in species that do not transmit the virus.

Several proteins investigated in this study may be important targets for human therapeutics but were identified as highly conserved, and therefore were considered unlikely to be drivers in determining species susceptibility to SARS-CoV-2 infection or transmission. These proteins include EIF4E2 (4EHP); the 40S ribosome subunits uS3 and uS5; G3BP1; GIGYF2; KPNA2; RAE1; SRP19; SRP54; STAT1; STAT2; TBK1; and TOMM70 (S2–S4, S7–S9, S16, S18–S21, S23, and S25 Figs). Five proteins lacked solved structures or data sufficient to determine critical amino acids: MAVS, POLA1, RIG-I, STING, and TMPRSS2 (S10, S15, S17, S22, and S24 Figs; S1 Table).

Tethrin (BST-2) was investigated because it is a protein found only in mammals that functions by tethering nascent mammalian enveloped virions to each other and to the membranes of infected cells, thus preventing their spread to other cells. BST-2 is IFN-induced (i.e., an ISG) [39]. Our analysis of the unbound structure (PDB 3NWH) with the MOE Site Finder tool indicated that residues in the pockets with the top two propensity for ligand binding scores matched those identified in an in silico analysis by Bisht et al. [23] (S2 Table, Table 1). Some evidence indicates that BST-2 might be a stronger barrier in restricting viral replication in bats than in humans [40]. However, with respect to the specific residues studied herein, no substitutions were conserved across bats that did not have similar substitutions in other mammals.

Information found for the remaining proteins (MDA5, NEMO, IRF3, and ISG15) revealed potential domains or specific amino acids for which substitutions may result in disruption of the PPI observed in humans, as discussed below.

Within the MDA5 N-terminus are NCBI domains cd08818 and cd08819, representing two caspase activation and recruitment domains (CARD), first and second repeats. The cd08818 domain contains sites that bind ISG15 (ISGylation; K23, K43, and K68), CARD oligomerization sites (G74 and W75), and serine phosphorylation sites (S88 and S104). According to the SeqAPASS Level 3 results, the ISGylation sites (crucial to antiviral responses [41,42]), and S88 had partial match substitutions in various species, with substitutions for a combination of one of the ISGylation lysines and S88 substituted by asparagine (S88N) in the Eastern cottontail rabbit, big and little brown bats, cow, house mouse, and Norway rat. The gray squirrel, Chinese tree shrew, pig, and Jamaican fruit bat also have the single S88N substitution (S11C Fig). Because serine can be phosphorylated and asparagine cannot, the S88N substitution might potentially affect interactions with SARS-CoV-2 PLpro. Additionally, ISGylation requires lysine, and substitutions were found to result in attenuated signaling in humans [42]. Furthermore, the mammals with a combination of lysine and S88N substitutions have generally shown COVID-19 resistance [4] (Table 3); therefore, ISGylation and phosphorylation in MDA5 may lead to differences in interactions compared to those in humans. Evidence of this possibility has been described in the literature [43,44]; therefore, the low conservation of MDA5 and ISG15 (Fig 1B) and the complexity of the ISGylation pathway contribute to the potential for variation in antiviral responses across species.

The interaction of the N-terminus of ORF9b with NEMO after viral infection interrupts its K63-linked polyubiquitination, thereby inhibiting IFNβ1 expression in HEK293T cells in an ORF9b-dose-dependent manner, however, binding residues were not determined [45]. For Level 2, domain cd09803 contains the polyubiquitin binding site that would be blocked by SARS-CoV-2 ORF9b [45]. This sequence was more than 90% conserved in mammalian orders Chiroptera, Rodentia, Artiodactyla, Carnivora, and Lagomorpha (S14B Fig). Hameedi et al. [46] solved the X-ray crystal structure of 3CLpro bound to NEMO and characterized 3CLpro cleavage of NEMO. An alanine substitution in the house mouse at the predicted binding hotspot V232 was considered to increase the propensity for helix-formation, thus potentially destabilizing house mouse NEMO, relative to the stronger interaction between human NEMO and the 3CLpro catalytic site [46]. This substitution exists in the resistant big and little brown bats, and the Norway rat, but is also found in the prairie deer mouse and golden hamster, both of which have tested positive for SARS-CoV-2 infection (S14C Fig; Table 3). Further investigation of the 3CLpro binding propensities to NEMO in other species is warranted.

Interferon regulatory factor 3 (IRF3) is the crucial factor in the pathway that, when activated and transported to the nucleus, binds DNA and induces expression of IFN-I. Crystal structures show interaction sites with the adaptor proteins STING (5JEJ), MAVS (5JEK), TRIF (5JEL), and a rotavirus protein (5JEO) that interferes with IRF3 activation by binding at the host adaptor protein sites (S2 Table). The adaptor protein binding site on IRF3 surrounds the SARS-CoV-2 Plpro cleavage location; cleavage would thwart activation by disrupting the adaptor protein binding site. NSP3 (Plpro) cleaves IRF3 at residues 267–273 (CLGGGLA), thereby decreasing the IRF3 available for induction of IFN-I expression [26]. Although variability is found across species throughout the protein (Fig 1; S12A Fig), as well as specifically in the adaptor binding site residues (S12C Fig) and the nearby conserved domain pfam10401 (S12B Fig), the most consequential residue according to Moustaqil et al. [26] is the cleavage site G270. At this position, the domestic ferret (R) and rodents (K, N) have substitutions with respect to the human protein. Strikingly, the queried bats showed conservation of the glycine as well as other residues in the cleavage motif except C267, a residue for which many species including primates have substitutions (S12C Fig). Further cellular experimentation is needed to determine the effects of IRF3 cleavage on SARS-CoV-2 infection in other species.

The function of ISG15 in antiviral immunity is direct inhibition of viral replication [47]. SARS-CoV-2 NSP3 (Plpro) preferentially cleaves ISG15 [48]. A house mouse crystal structure (PDB 6YVA) has been solved with Plpro bound to ISG15 [48]. In this study, we used an unbound human x-ray crystal structure (PDB 1Z2M) [49] as a template to which NSP3 was virtually docked using MOE and compared with the house mouse structure. Klemm et al. [50] generated a crystal structure of the C-terminal end of ISG15 bound to NSP3 (6XA9), and indicated that W123, P130, and E132 are important interacting residues. These residues were also determined by MOE to be interacting residues, but hydrogen bonding was not indicated at E132 (S2 Table). Variability in sequence and bonding was observed between the house mouse and human structures. Ionic and hydrogen bonds at K35 and E87 in mice were not found to be interacting in the human structures, but these bonds were present in both species at R153, and the hydrogen bonds matched between the two species in the conserved C-terminal motif, L154, R155, and G156. The conserved domain ISG15 cd01810 included these residues. Given the low ISG15 sequence conservation (Fig 1; S13 Fig), the differences between the house mouse and human structures’ bonding interactions, and the involvement of ISG15 in the ISGylation regulating several proteins in the IFN-I pathway (including MDA5, IRF3, STAT1, and RIG-I) [44], ISG15 appears to be critically important in determining cross-species variability in antiviral response.

As observed in the clustered heatmaps (Fig 1), conserved domains and specific interacting amino acids clustered according to taxonomic groups rather than empirical measures of infection. Importantly, small numbers of individuals have been tested for infection or sero-conversion within a species in some studies, and some results (pigs, ferrets, and rhesus monkeys) were inconsistent in interpretation among studies [4] (Table 3). Caveats in the empirical infection data include false positive or negative results, differences in testing strategies, and that some animals testing positive (“yes”) for viral RNA or seroconversion were described in reports in which other animals did not test positive; for example, in one study testing ten Malayan pangolins, only one tested positive [4]. Since the COVID-19 pandemic began, humans have shown variations in susceptibility, particularly with age and underlying conditions. Genetic factors such as gene regulation and splicing (e.g., in genes encoding antiviral 2′,5′-oligoadenylate synthetase [OAS] enzymes) have been found to affect human susceptibility [51]. Sequence variation in major histocompatibility complex (MHC) proteins has also been implicated in symptomatic versus asymptomatic cases [52]. Similar factors certainly exist in other mammalian species. In several studies in which animals were exposed to SARS-CoV-2, either naturally or experimentally, some individuals within a species were found to be infected, whereas others were not; moreover, some species appeared to be resistant, with none of the exposed individuals becoming infected [34]. Among the 43 species identified in Table 3 with data in the literature, 28 species were investigated for ability to transmit the virus, of which 13 showed no evidence of transmission or viral shedding. As discussed for the individual proteins, some evidence suggests that these differences might be explained by certain amino acid substitutions across species, in particular IFN-I pathway proteins investigated in this study.

Whereas other studies have attempted predictive ranking of species according to the numbers of critical amino acid substitutions, particularly in ACE2, the results of this study indicated that the numbers of substitutions did not directly correlate with species infection status. The compelling and novel finding of this study is the identification of specific amino acid substitutions associated with key structural or physiochemical features in certain proteins that may alter the interaction with the viral protein. Such substitutions potentially underlie the mechanism of resistance in the species that have tested negative for SARS-CoV-2 or have been found not to transmit the virus; however, the mechanisms might differ among resistant species. The substitutions of the ISGylation residues K23, K43, or K68, and serine phosphorylation S88N in MDA5 in several non-transmitting species (Eastern cottontail rabbit, big and little brown bats, cow, pig, house mouse, and Norway rat) include cattle and pigs, but not dogs or horses. The NEMO V232A substitution in the house mouse at the predicted binding hotspot [46], is substituted only in rodents, tree shrews, and big brown bats. The IRF3 cleavage site G270 substitution is found in rodents but not bats [26]. These and other IFN-I pathway proteins show potential for binding location differences; however, more experimental data are needed, at both the molecular and cellular levels, to define the infection and transmission capabilities of species of epidemiological concern with greater certainty. Additionally, several proteins had no PPI crystal or modeled structures—a general research gap hindering studies of protein or ligand interactions across non-human vertebrate species. Furthermore, missing and low-quality sequences identified through the sequence-based prediction approaches presented herein are limitations of the study, and highlight the need for more reliable sequences and genome coverage for protein interaction predictions across species.

## CONCLUSIONS

Our findings challenge previous assumptions regarding amino acid substitutions and pathogen-host protein interactions. This work demonstrated that susceptibility predictions cannot be based solely on the numbers and types of substitutions at the binding interfaces, in comparison to those in the most susceptible species. However, specific substitutions in NEMO, IRF3, and MDA5, in addition to ACE2, were found to potentially affect PPI binding affinity or protein modifications, such as ISGylation and phosphorylation, that do appear to correspond to species susceptibility. Our results also highlight the importance of evaluating both specific interaction-disrupting substitutions and overall structural similarity across species [53–56]. These findings will require further investigation as promising leads in the identification of intermediate hosts, thus providing important information for epidemiological monitoring of emerging zoonotic diseases.

## Supplementary Material

SupplementaryFile4

SupplementaryFigures

SupplementaryFile3

SupplementaryFile1

SupplementaryTables

SupplementaryFile2

## Figures and Tables

**TABLE 1 |:** Host protein target and protein-protein interaction information.

SARS-CoV-2 viral protein stressor	Host protein	Human protein length (amino acids)	National Center for Biotechnology Information (NCBI) Accession	UniProt Accession	Conserved domains^a^	Critical contact amino acids^b^ (numbered according to human host protein sequence)
Non-structural protein (NSP) 2	**4EHP (EIF4E2)**: Eukaryotic translation initiation factor 4E type 2	245	NP_004837.1	O60573	Not available (NA)	Not available (NA)
NSP1	**40S ribosomal subunit, uS3 (S3)**	243	NP_000996.2	P23396	**pfam00189:** Ribosomal_S3_C (105–188)	Bond: 12, 113, 114, 116, 117, 143, 148 Distance: 115, 142, 150, 177
	**40S ribosomal subunit, uS5 (S2)**	293	NP_002943.2	P15880	NA	Bond: 106, 146, 147 Distance: 105, 107, 109, 111, 113, 122, 124, 145, 148, 151
S (spike protein receptor binding domain [RBD])	**ACE2:** Angiotensin converting enzyme 2	805	NP_068576.1	Q9BYF1	NA	Bond: 19, 24, 30, 31, 35, 38, 41, 42, 83, 353 Distance:27, 28, 34, 37, 45, 79, 82, 330, 354, 355, 357, 393
Open reading frame (ORF) 7a	**BST-2**: Bone marrow stromal antigen 2 (tethrin)	180	NP_004326.1	Q10589	NA	Predicted pocket: 48, 49, 50, 51, 52, 53, 84, 85, 87, 88, 89, 91, 92, 105, 106, 108, 109, 110, 112, 113, 117
N (nucleocapsid)	**G3BP1**: ras GTPase-activating protein SH3 domain-binding protein	466	NP_005745.1	Q13283	**cd00780:** NTF2 (Nuclear transport factor domain 2) (7–135)	Bond: 15, 18, 32, 34, 117, 122, 123, 124 Distance: 6, 10, 11, 14, 33, 58, 121, 125
NSP2	**GIGYF2**: GRB10-interacting GYF protein 2	1299	NP_056390.2	Q6Y7W6	NA	Predicted binding region: 864, 877, 881–896
NSP3 Papain-like protease (Plpro)	**IRF3**: Interferon regulatory factor 3	427	NP_001562.1	Q14653	**cd00103:** IRF TF DNA recognition site (5–110) **pfam10401:** IRF-3 Superfamily res (202–380)	Bond: 211, 263, 285, 288, 290, 313, 349, 350, 351, 360 Distance: 260, 264, 287, 289, 292, 362 Cleavage site: 267, 268, 269, 270, 271, 272, 273
NSP3 Papain-like protease (Plpro)	**ISG15**: Ubiquitin-like IFN stimulated gene 15	165	NP_005092.1	P05161	**cd01792:** Ubl1_ISG15 (3–77) **cd01810:** Ubl2_ISG15 (82–155)	Bond: 22, 123, 127, 128, 130, 153, 154, 155, 156 Distance: 20, 23, 27, 30, 121, 129, 131, 132, 151, 152
ORF6	**KPNA2**: Karyopherin Subunit Alpha 2 (Importin subunit alpha-1)	529	NP_001307540.1	P52292	**pfam01749:** IBB importin beta binding domain (13–98)	NA
M (membrane)	**MAVS**: Mitochondrial antiviral signaling protein	540	NP_065797.2	Q7Z434	**cd08811:** CARD_IPS1 (3–93)	Bond: 436, 440, 441, 442, 456, 457, 458, 459 Distance: 438, 439,453,455
NSP3 Papain-like protease (Plpro)	**MDA5**: Melanoma differentiation-associated gene 5 (Interferon-induced helicase C domain-containing protein 1)	1025	NP_071451.2	Q9BYX4	**cd08818:** CARD_MDA5_ r1 (8–99) **cd08819:** CARD_MDA5_ r2 (111–201) **cd12090:** MDA5_ID (insert domain) (548–694) **cd15807:** MDA5_C (C-term) (900–1015)	ISGylation: 23, 43, 68 CARD oligomerization: 74, 75 Serine phosphorylation: 88, 104
ORF9b	**NEMO**: Nuclear factor kappa-B (NF-κB) essential modulator	419	NP_003630.1	Q9Y6K9	**cd09803: UBAN** (polyubiquitin binding domain of NEMO) (258–344)	NA
NSP5 3-chymotrypsin-like protease (3CLpro)	**NEMO**					Bond: 226, 228, 229, 230, 231, 232, 233, 234 Distance: 227, 235
NSP1	**POLA**1: DNA polymerase alpha 1, catalytic subunit	1462	NP_058633.2	P09884	**cd05776:** DNA_polB_alpha_exo (535–767)	Bond: 610, 613, 616, 617, 655 Distance: 594, 611, 614, 620, 621, 624, 625, 657
ORF6	**RAE1:** Ribonucleic acid export 1	368	NP_003601.1	P78406	NA	Bond: 239, 256, 257, 258, 305, 306, 307, 309, 310 Distance: 255, 300, 301, 302, 308, 365
N (nucleocapsid)	**RIG-I:** Retinoic acid-inducible gene I	925	NP_055129.2	O95786	**cd08816:** CARD_RIG-I_r1 (N-term) (2–92) **cd08817:** CARD_RIG-I_r2 (100–189) **pfam04851:** RES III (241–410)	NA
NSP9	**SRP19:** Signal recognition particle 19	144	NP_003126.1	P09132	NA	NA
NSP8	**SRP54**: Signal recognition particle	504	NP_003127.1	P61011	NA	NA
N (nucleocapsid), S (spike), NSP13 (helicase)	**STAT1:** Signal transducer and activator of transcription 1	750	NP_009330.1	P42224	**cd10372:** SH2_STAT1 (557–707)	Bond: 628, 629, 630, 631, 632, 651, 652 Distance: 584, 585, 588, 616, 627, 633, 634, 653 Phosphorylation sites: 701, 702, 703, 704, 705, 706, 707, 724, 727
N (nucleocapsid) Orf7a, Orf7b, NSP6, NSP13	**STAT2:** Signal transducer and activator of transcription 2	851	NP_005410.1	P52630	**cd10373:** SH2_STAT2 (556–706)	Phosphorylation sites: 583, 601, 627, 629, 690
NSP5 (3CLpro), ORF3a	**STING**: Stimulator of interferon genes	379	NP_938023.1	Q86WV6	**cd12146:** STING_C (C-terminal binding domain of STING) (155–337)	NA
NSP6	**TBK1**: TANK-binding kinase1	729	NP_037386.1	Q9UHD2	NA	NA
NSP13	**TBK1**				**cd13988:** STKc_TBK1 (15–330)	Predicted from model: 50, 51, 52, 54, 123, 127, 132, 133, 134, 135, 159, 177, 180, 181, 182, 183, 200, 201, 202, 203, 204, 206, 207, 232, 235, 289, 290, 294, 295
S (spike protein)	**TMPRSS2:** Transmembrane serine protease 2	492	NP_005647.3	O15393	**cd00190:** Tryp_SPc (Trypsin-like serine protease active/cleavage site) (256–487)	Bond: 296, 299, 338, 340, 341, 342, 389, 391, 392, 436, 438, 439, 441, 460, 462, 464 Distance: 280, 300, 419, 435, 437, 440, 459, 461, 463, 465, 470, 471, 472
ORF9b	**TOMM70**: Translocase of outer mitochondrial membrane	608	NP_055635.3	O94826	NA	Bond: 219, 225, 379, 381, 413, 447, 477, 484, 544, 545, 556, 580, 594 Distance: 215, 256, 259, 260, 340, 341, 375, 378, 409, 410, 412, 414, 443, 480, 481, 511, 515, 518, 521, 522, 546, 549, 550, 553, 557, 559, 561, 579, 583, 587, 590, 591, 598

a For conserved domains, amino acids within the domain are given in parentheses, numbered according to the human protein; see the Conserved Domain Database (ncbi.nlm.nih.gov/cdd).

b Critical amino acid interactions are identified by the type of contact as “bond” (hydrogen, metal, ionic, arene, or covalent) or “distance” (e.g., Van der Waals forces), or by function (see Materials and Methods).

**TABLE 2 |:** Structural basis for SARS-CoV-2 spike protein receptor-binding domain (RBD) amino acid interactions with specific angiotensin converting enzyme 2 (ACE2) receptor amino acids in several mammals.

Species	Human		Dog	Cat	Sea lion	Pangolin	Bat	Horse	Minke whale
Study	[27]	[28]	[29]	[12]	[30]	[31]	[32]	[33]	[30]
PDB	6VW1	6M0J	7E3J	7C8D	7WSH	7DHX	7XA7	7FC5	7WSE
**K**_**d**_ **mammal**			**123**	**85.70**	**170.69**	**34.97**	**448.3**	**11.04**	**175.98**
**(nM)** ± SE			NA	19.16	32.24	4.31	70	NA	40.81
**K**_**d**_ **human**			**18.8**	**21.73**	**25.68**	**10.19**	**22.4**	**4.39**	**25.68**
**(nM)** ± SE			NA	1.54	6.82	0.28	2.7	NA	6.82
**Spike RBD position**	**Host Angiotensin Converting Enzyme 2 (ACE2) amino acid position**								
Glu406							Arg34		
Lys417							Arg34		
Lys417	**Asp30**	**Asp30**	**Glu30**	**Glu30**	Glu30	**Glu30**	**Asp30**	**Glu30**	Gln30
Gly446	Gln42	Gln42	Gln42	Gln42	Gln42	Gln42	Gln42	Gln42	Arg42
Tyr449	Asp38	Asp38	Glu38	Glu38	Glu38	Glu38	Glu38	Glu38	Asp38
Tyr449	Gln42	Gln42	Gln42	Gln42	Gln42	Gln42	Gln42	Gln42	Arg42
Tyr453	His34	His34	Tyr34	His34φ	Ser34	Ser34	Arg34	Ser34	His34
Leu455	Asp30	Asp30	Glu30	Glu30	Glu30	Glu30	Asp30	Glu30	Gln30
Leu455	Lys31	Lys31		Lys31	Lys31	Lys31	Asn31	Lys31	Lys31
Leu455	His34	His34φ	Tyr34	His34	Ser34	Ser34	Arg34	Ser34	His34
Phe456	Asp30	Asp30	Glu30	Glu30	Glu30	Glu30	Asp30	Glu30	Gln30
Phe456	Lys31	Lys31	Lys31	Lys31	Lys31	Lys31	Asn31	Lys31	Lys31
Ala475	Ser19	Ser19	Ser19	Ser19	Ser19	Ser19	Ser19	Ser19	Ser19
Ala475	Gln24	Gln24	Leu24	Leu24	Leu24	Glu24	Arg24	Leu24	Gln24
Gly476	Ser19	Ser19		Ser19	Ser19	Ser19			
Gly476	Gln24	Gln24	Leu24	Leu24	Leu24	Glu24	Arg24	Leu24	Gln24
Ser477						Ser19			Ser19
Ser477	Gln24	Gln24							
Glu484	Lys31	Lys31	Lys31					Lys31	
Phe486	Met82	Met82	Thr82	Thr82		Asn82	Asn82	Thr82	Thr82
Phe486	Tyr83	Tyr83	Tyr83			Tyr83	Tyr83	Tyr83	Tyr83
Asn487	Gln24	Gln24	Leu24	Leu24	Leu24	Glu24	Arg24	Leu24	Gln24
Asn487	Tyr83	Tyr83	Tyr83	Tyr83	Tyr83	Tyr83	Tyr83	Tyr83	Tyr83
Tyr489	Gln24	Gln24	Leu24		Leu24	Glu24		Leu24	
Tyr489	Lys31	Lys31	Lys31	Lys31	Lys31	Lys31	Asn31	Lys31	Lys31
Tyr489	Tyr83	Tyr83	Tyr83	Tyr83	Tyr83	Tyr83	Tyr83	Tyr83	Tyr83
Gln493	Lys31	Lys31	Lys31	Lys31		Lys31	Asn31	Lys31	Lys31
Gln493	His34	His34		His34	Ser34	Ser34	Arg34	Ser34	His34
Gln493	Glu35	Glu35			Glu35	Glu35	Glu35	Glu35	Glu35
Ser494							Glu38		His34
Tyr495							Glu38		His34
Gly496	Asp38	Asp38	Glu38	Glu38	Glu38	Glu38	Glu38	Glu38	Asp38
Gly496	Lys353	Lys353	Lys353	Lys353	Lys353	Lys353	Lys353	Lys353	Lys353
Gln498	Asp38	Asp38	Glu38	Glu38	Glu38	Glu38		Glu38	Asp38
Gln498	Gln42	Gln42	Gln42	Gln42	Gln42	Gln42		Gln42	Arg42
Gln498	Lys353	Lys353	Lys353			Lys353	Lys353	Lys353	Lys353
Thr500	Asp355	Asp355	Asp355	Asp355	Asp355	Asp355	Asp355	Asp355	Asp355
Asn501	Lys353	Lys353	Lys353	Lys353	Lys353	Lys353	Lys353	Lys353	Lys353
Asn501				Asp355					Asp355
Gly502	Lys353	Lys353	Lys353	Lys353	Lys353	Lys353	Lys353	Lys353	Lys353
Gly502	Asp355	Asp355	Asp355	Asp355	Asp355		Asp355	Asp355	Asp355
Tyr505	Lys353φ	Lys353	Lys353	Lys353	Lys353	Lys353φ	Lys353φ	Lys353	Lys353φ

Interactions detected in the indicated structures from the Protein Data Bank (PDB) with the Molecular Operating Environment (MOE) software, contacts tool, limited to amino acids with hydrogen, ionic, or arene bonds (S1 File). Types of interactions are identified as follows: underline = hydrogen bond, **bold** = ionic bond, φ = arene/stacking bond, and all interacting pairs had distance bonds within 4.5 Angstroms (Å) (i.e., potential Van der Waals forces). Empty cells indicate no interaction detected. Blue = partial match to residue in human query sequence (conservative substitution). Yellow = not a match to human (non-conservative substitution), on the basis of SeqAPASS amino acid categorization. Green = indicated in literature as important contacts for binding (Damas et al. [9]; Luan et al. [10,11]). Binding affinity (K_d_) between the ACE2 and SARS-CoV-2 spike protein receptor-binding domain (RBD) was determined by the authors of each referenced study for humans and the respective species of interest; nM = nanomoles/L, ± standard error (SE); NA = no SE reported.

**TABLE 3 |:** Empirical data indicating confirmed measures of SARS-CoV-2 infection and transmission for known tested species.

Family	Genus/species	Common name	Viral RNA	Sero-conversion	Transmission or shedding	Sequences available
Class Mammalia, superorder Euarchontoglires						
**Order Primates**						
Hominidae	*Homo sapiens*	Human	Yes	Yes	Yes	Yes
	*Gorilla gorilla gorilla*	Western lowland gorilla	Yes	Yes	Yes	Yes
Cercopithecidae	*Chlorocebus sabaeus*	Green monkey	Yes	Yes	Yes	Yes
	*Papio anubis*	Olive baboon	Yes	ni	ni	Yes
	*Macaca mulatta*	Rhesus monkey	Yes	Y/N	ni	Yes
Cebidae	*Callithrix jacchus*	White-tufted-ear marmoset	Yes	ni	ni	Yes
**Order Scandentia**						
Tupaiidae	*Tupaia chinensis*	Chinese tree shrew	Yes	ni	ni	Y/N
**Order Lagomorpha**						
Leporidae	*Oryctolagus cuniculus*	Rabbit	Yes	Yes	ni	Y/N
	*Sylvilagus floridanis*	Eastern cottontail rabbit	No	No	No	No
**Order Rodentia**						
Sciuridae	*Neosciurus carolinensis*	Gray squirrel	Yes	Yes	ni	Yes
	*Urocitellus elegans*	Wyoming ground squirrel	No	No	No	No
	*Sciurus niger*	Fox squirrel	No	No	No	No
	*Cynomys ludovicianus*	Black-tailed prairie dog	No	No	No	No
Cricetidae	*Peromyscus maniculatus bairdii*	Prairie deer mouse	Yes	Yes	Yes	Yes
	*Mesocricetus auratus*	Golden hamster	Yes	Yes	Yes	Yes
	*Neotoma cinerea*	Bushy-tailed wood rat†	Yes	Yes	Yes	No
	*Myodes glareolus*	Bank vole	Yes	Yes	No	Y/N
Muridae	*Rattus norvegicus*	Norway rat^‡^	No	No	No	Yes
	*Mus musculus*	House mouse^¶^	No	No	No	Yes
Class Mammalia, superorder Laurasiatheria						
**Order Artiodactyla**						
Cervidae	*Odocoileus virginianus texanus*	White-tailed deer	Yes	Yes	Yes	Yes
Bovidae	*Bos taurus*	Cow	Yes	Yes	No	Yes
Equidae	*Equus caballus*	Horse^§^	No	Yes	No	Y/N
Suidae	*Sus scrofa*	Pig	Y/N	Y/N	No	Yes
**Order Carnivora**						
Canidae	*Nyctereutes procyonoides*	Raccoon dog	Yes	Yes	Yes	Yes
	*Canis lupus familiaris*	Dog	No	Yes	No	Yes
	*Vulpes vulpes*	Red fox†	Yes	Yes	Yes	Yes
	*Canis latrans*	Coyote	No	No	No	No
Felidae	*Lynx canadensis*	Canada lynx	Yes	ni	ni	Yes
	*Felis catus*	Domestic cat	Yes	Yes	Yes	Yes
	*Prionailurus viverrinus*	Fishing cat	Yes	ni	ni	Y/N
	*Panthera tigris*	Tiger	Yes	Yes	ni	Yes
	*Lynx rufus*	Bobcat	Yes	ni	ni	Yes
Mustelidae	*Mustela putorius furo*	Domestic ferret	Yes	Y/N	Yes	Yes
	*Neogale vison*	American mink	Yes	Yes	Yes	Yes
	*Meles meles*	Eurasian badger	No	Yes	ni	Yes
	*Aonyx cinereus*	Asian small-clawed otter	Yes	ni	ni	No
	*Mustela lutreola*	European mink	Yes	Yes	Yes	No
Procyonidae	*Procyon lotor*	Raccoon	No	Yes	No	Y/N
Viverridae	*Arctictis binturong*	Binturong (bearcat)	Yes	ni	ni	No
Mephitidae	*Mephitis mephitis*	Striped skunk†	Yes	Yes	Yes	No
Hyenidae	*Crocuta crocuta*	Spotted hyena	Yes	ni	ni	Y/N
**Order Pholidota**						
Manidae	*Manis javanica*	Malayan pangolin	ni	Yes	ni	Yes
**Order Chiroptera**						
Vespertilionidae	*Eptesicus fuscus*	Big brown bat	Yes	No	ni	Y/N
Pteropodidae	*Rousettus aegyptiacus*	Egyptian rousette	Yes	Yes	Yes	Yes
**Other classes**						
Class Aves	*Columba livia*	Rock pigeon	No	No	No	Y/N
Class Crocodylia	*Alligator sinensis*	Chinese alligator	No	No	No	Yes
Class Amphibia	*Xenopus laevis*	African clawed frog	No	No	No	Yes
Class Actinopteri	*Pimephales promelas*	Fathead minnow	No	No	No	Yes

All data were compiled in EFSA/Nielsen et al. [4] except where noted. Susceptibility indicators: yes = detected or transmitted; no = not detected (or no evidence of transmission); ni = not investigated; Y/N = equivocal results: studies had different findings. Sequence availability: yes = sequences available for all investigated proteins; no = no sequences were available for that species; Y/N = some complete sequences were available/some sequences were partial or rejected because of to low quality.

† Species in which oral/nasal viral shedding was found, but direct transmission was not investigated (woodrat, skunk [34] and red fox [35]).

‡ On the basis of Norway rats sampled in the sewer system in Antwerp, Belgium [37].

¶ The house mouse resists viral entry of the original Wuhan strain [36].

§ Racehorses tested in California through 2020 [38].
