# Supplementary material for: In Silico Analysis of Cross-Species Sequence Variability in Host Interferon Antiviral Pathway Proteins and SARS-CoV-2 Susceptibility
Source: Zoonoses. Author manuscript; Available in PMC 2026 Mar 11. (PMC12973230; doi:10.15212/ZOONOSES-2024-0028)
Supplement: SupplementaryFile4 [file NIHMS2123055-supplement-SupplementaryFile4.docx]

**Cross-species sequence variability in host interferon antiviral pathway proteins and SARS-CoV-2 susceptibility.**

**Supplementary File 4**

**Documentation for Sequence Curation (see Supplementary files 1, 2, and 3: SeqAPASS Level 1, 2, and 3 curated full reports, respectively).**

40S uS3 (S3) no change Level1. Big brown bat Level 2 change: record XP_00814205.1 obsolete; updated in NCBI.

Human [NP_000996.2](https://www.ncbi.nlm.nih.gov/protein/NP_000996.2?report=genbank&log$=protalign&blast_rank=0&RID=CJZ8AEGX016) Big brown bat [XP_054581605.1](https://www.ncbi.nlm.nih.gov/protein/XP_054581605.1?report=genbank&log$=protalign&blast_rank=1&RID=CJZ8AEGX016)

Human 1 MAVQISKKRKFVADGIFKAELNEFLTRELAEDGYSGVEVRVTPTRTEIIILATRTQNVLGEKGRRIRELTAVVQKRFGFP 80

BBBat 1 MAVQISKKRKFVADGIFKAELNEFLTRELAEDGYSGVEVRVTPNRTEIIILATRTQNVLGEKGRRIRELTAVVQKRFGFP 80

Human 81 EGSVELYAEKVATRGLCAIAQAESLRYKLLGGLAVRRACYGVLRFIMESGAKGCEVVVSGKLRGQRAKSMKFVDGLMIHS 160 BBBat 81 EGSVELYAEKVATRGLCAIAQAESLRYKLLGGLAVRRACYGVLRFIMESGAKGCEVVVSGKLRGQRAKSMKFVDGLMIHS 160

Human161 GDPVNYYVDTAVRHVLLRQGVLGIKVKIMLPWDPTGKIGPKKPLPDHVSIVEPKDEILPTTPISEQKGGKPEPPAMPQPV 240 BBBat161 GDPVNYYVDTAVRHVLLRQGVLGIKVKIMLPWDPSGKIGPKKPLPDHVSIVEPKDEILPTTPISEQKGGKPEPPAMPQPV 240

Human241 PTA 243

BBBat241 PTA 243

40S uS5(S2) Level 1 sequence correction for fox (same as dog). No Level 2 domains.

human [NP_002943.2](https://www.ncbi.nlm.nih.gov/protein/NP_002943.2?report=genbank&log$=protalign&blast_rank=0&RID=B5WSWAJW013) Dog [XP_022275969.1](https://www.ncbi.nlm.nih.gov/protein/XP_022275969.1?report=genbank&log$=protalign&blast_rank=1&RID=B5WSWAJW013) Racoondog [XP_055159820.1](https://www.ncbi.nlm.nih.gov/protein/XP_055159820.1?report=genbank&log$=protalign&blast_rank=2&RID=B5WSWAJW013) Fox [XP_025840693.1](https://www.ncbi.nlm.nih.gov/protein/XP_025840693.1?report=genbank&log$=protalign&blast_rank=3&RID=B5WSWAJW013)

Human 1 MADDAGAAGGPGGPGGPG---MGNRGGFRGGFGSGIRGRGRGRGRGRGRGRGARGGKAEDKEWMPVTKLGRLVKDMKIKS 77

Dog 1 MADDAGAAGGPGGPGGPG---MGGRGGFRGGFGSGIRGRGRGRGRGRGRGRGARGGKAEDKEWIPVTKLGRLVKDMKIKS 77

Rdog 1 MADDAGAAGGPGGPGGPGgpgMGGRGGFRGGFGSGIRGHGRGRGRGWGRGRGARGGKAEDKEWIPVTKLGRLVKDMKIKS 80

Fox 1 ---------------------MGGRGGFRGGFGSGIRGRGRGRGRGRGRGRGARGGKAEDKEWIPVTKLGRLVKDMKIKS 59

Human 78 LEEIYLFSLPIKESEIIDFFLGASLKDEVLKIMPVQKQTRAGQRTRFKAFVAIGDYNGHVGLGVKCSKEVATAIRGAIIL 157

Dog 78 LEEIYLFSLPIKESEIIDFFLGASLKDEVLKIMPVQKQTRAGQRTRFKAFVAIGDYNGHVGLGVKCSKEVATAIRGAIIL 157

Rdog 81 LEEIYLFSLPIKESEIIDFFLGASLKDEVLKIMPVQKQTRAGQRTRFKAFVAIGDYNGHVGLGVKCSKEVATAIHGAIIL 160

Fox 60 LEEIYLFSLPIKESEIIDFFLGASLKDEVLKIMPVQKQTRAGQRTRFKAFVAIGDYNGHVGLGVKCSKEVATAIRGAIIL 139

Human158 AKLSIVPVRRGYWGNKIGKPHTVPCKVTGRCGSVLVRLIPAPRGTGIVSAPVPKKLLMMAGIDDCYTSARGCTATLGNFA 237

Dog 158 AKLSIVPVRRGYWGNKIGKPHTVPCKVTGRCGSVLVRLIPAPRGTGIVSAPVPKKLLMMAGIDDCYTSARGCTATLGNFA 237

Rdog 161 AKLSIVPVRRGYWGNKIGKPHTVPCKVTDRCGSVLVRLIPAPRGTGIVSAPVPKKLLMMAGIDDCYTSARGCTATLGNFA 240

Fox 140 AKLSIVPVRRGYWGNKIGKPHTVPCKVTGRCGSVLVRLIPAPRGTGIVSAPVPKKLLMMAGIDDCYTSARGCTATLGNFA 219

Human238 KATFDAISKTYSYLTPDLWKETVFTKSPYQEFTDHLVKTHTRVSVQRTQAPAVATT 293

Dog 238 KATFDAISKTYSYLTPDLWKETVFTKSPYQEFTDHLVKTHTRVSVQRTQAPAVATT 293

Rdog 241 KATFDAISKTYSYLTPDLWKETVFTKSPYQEFTDHLVKTHTRVSMQRTQAPAVATT 296

Fox 220 KATFDAISKTYSYLTPDLWKETVFTKSPYQEFTDHLVKTHTRVSVQRTQAPAVATT 275

4EHP Level 1 raccoon dog same as dog (isoform differences). No Level 2 domains.

ACE2

Bobcat predicted sequence has alt start site. Assumed same as Canada lynx

Human [NP_001358344.1](https://www.ncbi.nlm.nih.gov/protein/NP_001358344.1?report=genbank&log$=protalign&blast_rank=0&RID=C0BZJT3W013) Canada lynx [XP_030160839.1](https://www.ncbi.nlm.nih.gov/protein/XP_030160839.1?report=genbank&log$=protalign&blast_rank=1&RID=C0BZJT3W013) Bobcat [XP_046931882.1](https://www.ncbi.nlm.nih.gov/protein/XP_046931882.1?report=genbank&log$=protalign&blast_rank=2&RID=C0BZJT3W013)

Human 1 MSSSSWLLLSLVAVTAAQSTIEEQAKTFLDKFNHEAEDLFYQSSLASWNYNTNITEENVQNMNNAGDKWSAFLKEQSTLA 80

CLynx 1 MSGSFWLLLSFAALAAAQSTTEELAKTFLEKFNHEAEELSYQSSLASWNYNTNITDENVQKMNEAGAKWSAFYEEQSKLA 80

Bcat --------------------------------------------------------------------------------

Human 81 QMYPLQEIQNLTVKLQLQALQQNGSSVLSEDKSKRLNTILNTMSTIYSTGKVCNPDNPQECLLLEPGLNEIMANSLDYNE 160

Clynx 81 KTYPLAEIHNTTIKRQLQALQQSGSSVLSADKSQRLNTILNAMSTIYSTGKACNPNNPQECLLLEPGLDDIMENSKDYNE 160

Bcat 1 ------------------------------------------MSTIYSTGKACNPNNPQECLLLEPGLDDIMENSKDYNE 38

Human161 RLWAWESWRSEVGKQLRPLYEEYVVLKNEMARANHYEDYGDYWRGDYEVNGVDGYDYSRGQLIEDVEHTFEEIKPLYEHL 240

Clynx161 RLWAWEGWRAEVGKQLRPLYEEYVALKNEMARANNYEDYGDYWRGDYEEEWTDGYNYSRSQLIKDVEHTFTQIKPLYQHL 240

Bcat 39 RLWAWEGWRAEVGKQLRPLYEEYVALKNEMARANNYEDYGDYWRGDYEEEWTDGYNYSRSQLIKDVEHTFTQIKPLYQHL 118

Human241 HAYVRAKLMNAYPSYISPIGCLPAHLLGDMWGRFWTNLYSLTVPFGQKPNIDVTDAMVDQAWDAQRIFKEAEKFFVSVGL 320

CLynx241 HAYVRAKLMDTYPSRISPTGCLPAHLLGDMWGRFWTNLYPLTVPFGQKPNIDVTDAMVNQSWDARRIFKEAEKFFVSVGL 320

Bcat 119 HAYVRAKLMDTYPSRISPTGCLPAHLLGDMWGRFWTNLYPLTVPFGQKPNIDVTDAMVNQSWDARRIFKEAEKFFVSVGL 198

Human321 PNMTQGFWENSMLTDPGNVQKAVCHPTAWDLGKGDFRILMCTKVTMDDFLTAHHEMGHIQYDMAYAAQPFLLRNGANEGF 400

CLynx321 PNMTQGFWENSMLTEPGDSRKVVCHPTAWDLGKGDFRIKMCTKVTMDDFLTAHHEMGHIQYDMAYAVQPFLLRNGANEGF 400

Bcat 199 PNMTQGFWENSMLTEPGNSRKVVCHPTAWDLGKGDFRIKMCTKVTMDDFLTAHHEMGHIQYDMAYAVQPFLLRNGANEGF 278

ACE2 missing C-term end of white-tailed deer sequence:

>XP_020730366.1 angiotensin-converting enzyme 2-like [Odocoileus virginianus texanus]

MYLFRSSVAYAMRKYFLKERNETIPFGEENVWVSDKKPRISFKFFVTSPNNVSDIIPRTEVENAIRLSRS

RINDAFQLDDNSLEFLGIQPTLGPPYEPPVTIWLIIFGVVMGVVVIGIVVLIFTGIRDRRKKNQASSEEN

PYGSVDLNKGENNSGFQNTDDVQTSL

Human561 GKSEPWTLALENVVGAKNMNVRPLLNYFEPLFTWLKDQNKNSFVGWSTDWSPYADQSIKVRISLKSALGDKAYEWNDNEM 640

Cattl560 GKSEPWTLALENIVGIKTMDVKPLLNYFEPLFTWLKEQNRNSFVGWSTEWTPYSDQSIKVRISLKSALGENAYEWNDNEM 639

Deer 560 GKSEPWTLALESIVGIKTMDVKPLLNYFEPLFTWLKEQNRNSFVGWSTEWTPYSDQSIKVRISLKSALGKNA--WNDNEM 1

Human641 YLFRSSVAYAMRQYFLKVKNQMILFGEEDVRVANLKPRISFNFFVTAPKNVSDIIPRTEVEKAIRMSRSRINDAFRLNDN 720

Cattl640 YLFQSSVAYAMRKYFSEARNETVLFGEDNVWVSDKKPRISFKFFVTSPNNVSDIIPRTEVENAIRLSRDRINDVFQLDDN 719

Deer YLFRSSVAYAMRKYFLKERNETIPFGEENVWVSDKKPRISFKFFVTSPNNVSDIIPRTEVENAIRLSRSRINDAFQLDDN

Human721 SLEFLGIQPTLGPPNQPPVSIWLIVFGVVMGVIVVGIVILIFTGIRDRKKKNKARSGENPYASIDISKGENNPGFQNTDD 800

Cattl720 SLEFLGIQPTLGPPYEPPVTIWLIIFGVVMGVVVIGIVVLIFTGIRNRRKKNQASSEENPYGSVDLNKGENNSGFQNIDD 799

Deer SLEFLGIQPTLGPPYEPPVTIWLIIFGVVMGVVVIGIVVLIFTGIRDRRKKNQASSEENPYGSVDLNKGENNSGFQNTDD

Human801 VQTSF 805

Cattl800 VQTSL 804

Deer VQTSL 513

BST-2 no changes Level 1.

G3BP1 no changes Level 1, Level 2.

GIGYF2 Level 1 Cattle percent similarity adjusted based on NCBI BLAST and similar artiodactyl species (equivalent hit length/identity/positives). Level 3 860-919 NSP2 binding zone (see Xu 2022). 864,877,881-896 were the only residues with substitutions in the known Covid status species.

IRF3 no changes in mammals Level 1, Level 2. No IRF3 in birds, IRF7 in fathead similar function.

ISG-15

ISG15 hit for Gorilla Polyubiquitin-b replaced by PREDICTED ubiquitin-like protein ISG15

>XP_055237821.1 ubiquitin-like protein ISG15 [Gorilla gorilla gorilla]

Level 1 and Level 2 cd01792 (3-77)

CLUSTAL O(1.2.4) multiple sequence alignment

Human_NP_005092.1 MGWDLTVKMLAGNEFQVSLSSSMSVSELKAQITQKIGVHAFQQRLAVHPSGVALQDRVPL 60

Gorilla_XP_055237821.1 MGWDLTVKMLAGNEFQVSLSNSMSVSELKAQITQKIGVHAFQQRLAVHPSGVALQDGVPL 60

********************.*********************************** ***

Human_NP_005092.1 ASQGLGPGSTVLLVVDKCDEPLSILVRNNKGRSSTYEVRLTQTVAHLKQQVSGLEGVQDD 120

Gorilla_XP_055237821.1 ASQGLGPGSTVLLVVDKCDEPLNILVRNNKGRSSTYEVRLTQTVAHLKQQVSGLEDVQDD 120

**********************.********************************.****

Human_NP_005092.1 LFWLTFEGKPLEDQLPLGEYGLKPLSTVFMNLRLRGGGTEPGGRS 165

Gorilla_XP_055237821.1 LFWLTFEGKPLEDQLPLGEYGLKPLSTVFMNLRLRGGGAEPGGRS 165

**************************************:******

ISG15 Level 2 cd01792 green monkey corrected to same as other monkeys (equivalent identity/positives).

ISG15 Level 2 cd01810 aa’s 82-155 hits

[NP_005092.1](https://www.ncbi.nlm.nih.gov/protein/NP_005092.1?report=genbank&log$=protalign&blast_rank=0&RID=ASN106EX013) [XP_055237821.1](https://www.ncbi.nlm.nih.gov/protein/XP_055237821.1?report=genbank&log$=prottop&blast_rank=1&RID=C7UGTR06013) [XP_003918909.1](https://www.ncbi.nlm.nih.gov/protein/XP_003918909.1?report=genbank&log$=protalign&blast_rank=1&RID=ASN106EX013) [NP_001253735.1](https://www.ncbi.nlm.nih.gov/protein/NP_001253735.1?report=genbank&log$=protalign&blast_rank=2&RID=ASN106EX013) [BAG50252.1](https://www.ncbi.nlm.nih.gov/protein/BAG50252.1?report=genbank&log$=protalign&blast_rank=3&RID=ASN106EX013) [XP_044092193.1](https://www.ncbi.nlm.nih.gov/protein/XP_044092193.1?report=genbank&log$=protalign&blast_rank=4&RID=ASN106EX013) [XP_004781339.1](https://www.ncbi.nlm.nih.gov/protein/XP_004781339.1?report=genbank&log$=protalign&blast_rank=5&RID=ASN106EX013)

Human 81 PLSILVRNNKGRSSTYEVRLTQTVAHLKQQVSGLEGVQDDLFWLTFEGKPLEDQLPLGEYGLKPLSTVFMNLRLRGG 157

Gorill81 PLNILVRNNKGRSSTYEVRLTQTVAHLKQQVSGLEDVQDDLFWLTFEGKPLEDQLPLGEYGLKPLSTVFMNLRLRGG 157

Baboon81 PLSILVRNDKGRSSTYEVQLTQTVAHLKQQVSQQEGVQDDLFWLTFEGKPLENQLPLGEYGLKPLSTVFMNLRLRGG 157

Rhesus81 PLSILVRNDKGRSSTYEVQLTQTVAHLKQQVSRQEGVQDDLFWLTFEGKPLENQLPLGEYGLKPLSTVFMNLRLRGG 157

Cat 81 PLSILVRNHKGRTIAYEVRLTQTVAELKQQICQQEHVQADLFWLNFEGKPMEDQHRLGEYELTPQCTLIMNLRLRGG 157

Ferret81 PLSILVRNDKGRSTAYEVRLTQTVAELKQQVCGQEHVQADLFWLSFQGKPMEDPHRLGDYGLTPQCTVFMNLRLRGG[4] 161

Mink 81 RLSILVRNDKGRSTAYEVRLTQTVAELKQQVCRQEHVQADLFWLSFQGKPMEDPHRLGDYGVTPQCTVFMNLRLRGG[2] 159

KPNA2 Level 1 correction for white-tailed deer percent similarity by extrapolation, similar to cattle. Correction for raccoon dog based on combined sequences CAD7668147.1 and XP_055193428.1 (same as dog). Malayan pangolin based on sequence KAI5929135.1 (replace XP_036852809.1). Level 2 change:

KPNA2 Importin subunit alpha-1, Level 2 pfam01749 13-98 (86) anomalous deer and pangolin

Goril same human[XP_030867589.1](https://www.ncbi.nlm.nih.gov/protein/XP_030867589.1?report=genbank&log$=protalign&blast_rank=0&RID=AV081K9K016) MjavPang[KAI5929135.1](https://www.ncbi.nlm.nih.gov/protein/KAI5929135.1?report=genbank&log$=protalign&blast_rank=2&RID=AV081K9K016) Cattle [NP_001029621.1](https://www.ncbi.nlm.nih.gov/protein/NP_001029621.1?report=genbank&log$=protalign&blast_rank=2&RID=AV081K9K016) Deer [XP_020757406.1](https://www.ncbi.nlm.nih.gov/protein/XP_020757406.1?report=genbank&log$=protalign&blast_rank=3&RID=AV081K9K016)

Human 1 MSTNENANTPAARLHRFKNKGKDSTEMRRRRIEVNVELRKAKKDDQMLKRRNVSSFPDDATSPLQENRNNQGTVN 75 Pang 1 [124]MSTNENANSPAARLNRFKNKGKDTTEMRRRRIEVNVELRKAKKDDQMLKRRNVSSFPDDATSPLQ-NRNNQGTVN 198 Cattle1 MSTNENANSPAPRLNRFKNKGKDSTEMRRRRIEVNVELRKAKKDDQMLKRRNVSSFPDDATSPLQENRNNQGTVN 75 Deer 1 --------------------------MRRCRIEVSVELRKAKKDDQMLKRRNVSSFPDDATSPLQENRNNQGTVN 49

Human 76 WSVDDIVKGINSSNVENQLQATQAARKLLSREKQPPIDNIIRAGLIPKFVSFLGRTDCSPIQFESAWALTNIASGTSEQT 155

Pang 199 WTVDDIVKGINGSSLESQLQATQAARKLLSREKQPPIDNIIRAGLIPKFVSFLGRTDCSPIQFESAWALTNIASGTSEQT 278

Cattle76 WSVDDIVKGINSNNLESQLQATQAARKLLSREKQPPIDNIIRAGLIPKFVSFLGRTDCSPIQFESAWALTNIASGTSEQT 155

Deer 50 WSVDDIVKGINSNNLESQLQAT

MAVS no changes Level 1, Level 2.

MDA5 Level 1:

PREDICTED: Odocoileus virginianus texanus interferon-induced helicase C domain-containing protein 1-like (LOC110129611), partial mRNASequence ID: XM_020881079.1, protein sequence [XP_020736738.1](https://www.ncbi.nlm.nih.gov/protein/XP_020736738.1?report=genbank&log$=protalign&blast_rank=2&RID=C83FXTPP01N) for the N-terminal to ~360 and [XP_020737513.1](https://www.ncbi.nlm.nih.gov/protein/XP_020737513.1?report=genbank&log$=protalign&blast_rank=2&RID=C83FXTPP01N) (hit from SeqAPASS) from there to the C-terminal end. Equivalent to Cattle. Level 2 cd08818 8-99 and cd08819 111-201 are highlighted.

Human_NP_071451.2 -MSNGYSTDENFRYLISCFRARVKMYIQVEPVLDYLTFLPAEVKEQIQRTVATSGNMQAV 59

Deer_1 MSSEESFADKIFCYLISCFRARVKRYIQVEPVLDYLTFLSPEVKEHIQRTAATSGDIQAA 60

Cattle_AYD78163.1 MSSDGCSTDKNFCYLISCFRARVKRYIQVEPVLDYLTFLPPEVKEHIQRTAATTGDIQAA 60

*: :*: * *********** ************** ****:****.**:*::**.

Human_NP_071451.2 ELLLSTLEKGVWHLGWTREFVEALRRTGSPLAARYMNPELTDLPSPSFENAHDEYLQLLN 119

Deer_1 DLLLNTLERGSWPLGWARMFVEALRQAGNPLAARYVNPELTDLPSPSSENAHDECLQLLN 120

Cattle_AYD78163.1 DLLLNTLERGNWPLGWARMFVEALRQAGNPLAARYVNPELTDLPSPSSENAHDECLQLLN 120

:***.***:* * ***:* ******::*.******:*********** ****** *****

Human_NP_071451.2 LLQPTLVDKLLVRDVLDKCMEEELLTIEDRNRIAAAENNGNESGVRELLKRIVQKENWFS 179

Deer_1 LLQPTLVDKLLVADVLDKCVEENLLTIEDRNRVSAAENNGNEAGVRELLKRIVQKENWFS 180

Cattle_AYD78163.1 LLQPTLVDKLLVADVLDKCVEEKLLTIEDRNRVSAAENNGNEAGVRELLKRIVQKENWFS 180

************ ******:**:*********::********:*****************

Human_NP_071451.2 AFLNVLRQTGNNELVQELTGSDCSESNAEIENLSQVDGPQVEEQLLSTTVQPNLEKEVWG 239

Deer_1 TFLTILRQTGNDELARELTGTDCYEGNTDSENLSQEDGLEVKEPLLLATDQPAL--EVWD 238

Cattle_AYD78163.1 TFLTILRQTGNDALAREFTGTDCCEGSTESENLSQEDGLEVKEPLLLATDQPNL--EVLD 238

:**.:******: *.:*:**:** *..:: ***** ** :*:* ** :* ** * ** .

Human_NP_071451.2 MENNSSESSFADSSVVSESDTSLAEGSVSCLDESLGHNSNMGSDSGTMGSDSDEENVAAR 299

Deer_1 IENSSLESSFADSSIVSESDTSLAEGSVSCLDESLGHNSNMGSDSGTMGSDSDDENVAQR 298

Cattle_AYD78163.1 IENSSLESSFADSSIVSESDTSLAEGSVSCLDESLGHNSNMGSDSGTMGSDSDDENVAQR 298

:**.* ********:**************************************:**** *

Human_NP_071451.2 ASPEPELQLRPYQMEVAQPALEGKNIIICLPTGSGKTRVAVYIAKDHLDKKKKASEPGKV 359

Deer_1 ASPEPELNLRPYQLEVAQPALEGKNIIICLPTGSGKTRVAVYIAKDHLDKKKKASEPGKV 358

Cattle_AYD78163.1 ASPEPELNLRPYQLEVAQPALEGKNIIICLPTGSGKTRVAVYIAKDHLDKK-KASEHGKV 357

*******:*****:************************************* **** ***

MDA5 Level 2 cd08818 8-99

Human [NP_071451.2](https://www.ncbi.nlm.nih.gov/protein/NP_071451.2?report=genbank&log$=protalign&blast_rank=0&RID=BDRY8GNB013) EuroBadger [XP_045874119.1](https://www.ncbi.nlm.nih.gov/protein/XP_045874119.1?report=genbank&log$=protalign&blast_rank=1&RID=BDRY8GNB013) Chinese pangolin [XP_057361470.1](https://www.ncbi.nlm.nih.gov/protein/XP_057361470.1?report=genbank&log$=protalign&blast_rank=2&RID=BDRY8GNB013) Malayan pangolin [XP_036851932.1](https://www.ncbi.nlm.nih.gov/protein/XP_036851932.1?report=genbank&log$=protalign&blast_rank=3&RID=BDRY8GNB013)

Human 1 MSNGYSTDENFRYLISCFRARVKMYIQVEPV-LDYLTFLPAEVKEQIQRTVATSGNMQAVELLLSTLEKGVWHLGWTREF 79 Badgr 1 MSNGYSADKSFCYLISCFRARVKTYIQVEPV-LDYLDFLPNDLKEQIQRTAVNAGNMQAADELLNALEKGGWPPGWTRQF 79

Cpang 1 MLNAYSADKXFRSPISCFTPGGNRDIQAEPV-LDFLGFLPAEMREXIQRPAAPAGNLCAVEQLLSTLENGVWPLGWTRKF 79

Mpang 1 MSNAYSADKSFRSPISASHPEGTGTSRRRLYwTDYLGFLPAEMREQIQRPAAPAGNLCAVEQLLSTLENGVWPPGWTRKF 80

Human 80 VEALRRTGSPLAARYMNPELTDLPSPSFENAHDEYLQLLNLLQPTLVDKLLVRDVLDKCMEEELLTIEDRNRIAAAENNG 159

Badgr 80 LVALQRAGSVLAARYLNPELTDLPSPSSENAHDECLQLLNLLQPTLVDRLLVRDVLDKCVEKKLLTNEDRNRISAAENNG 159

Cpang 80 VEALERTGSRLAARCVNPELX---SPSFGNTHDECPQLMSRLQPTLVDTLEVRDVVDKRAEEDLXATEDRNRIVAAENIG 156

Mpang 81 VEALERTGSRSAARCVNPEL

MDA5 Level 2 cd08819 111-201

Human [NP_071451.2](https://www.ncbi.nlm.nih.gov/protein/NP_071451.2?report=genbank&log$=protalign&blast_rank=0&RID=BDW16TM1016) Big brown bat [XP_008136853.2](https://www.ncbi.nlm.nih.gov/protein/XP_008136853.2?report=genbank&log$=protalign&blast_rank=1&RID=BDW16TM1016) Little brown bat [XP_006083271.1](https://www.ncbi.nlm.nih.gov/protein/XP_006083271.1?report=genbank&log$=protalign&blast_rank=2&RID=BDW16TM1016) Dog [XP_038302827.1](https://www.ncbi.nlm.nih.gov/protein/XP_038302827.1?report=genbank&log$=protalign&blast_rank=3&RID=BDW16TM1016) Red fox [XP_025849933.1](https://www.ncbi.nlm.nih.gov/protein/XP_025849933.1?report=genbank&log$=protalign&blast_rank=4&RID=BDW16TM1016) Raccoon dog [XP_055163859.1](https://www.ncbi.nlm.nih.gov/protein/XP_055163859.1?report=genbank&log$=protalign&blast_rank=5&RID=BDW16TM1016)

Human 80 VEALRRTGSPLAARYMNPELTDLPSPSFENAHDEYLQLLNLLQPTLVDKLLVRDVLDKCMEEELLTIEDRNRIAAAENNG 159

Bbat 80 VEALRRAGNPLAARYMDPELTDLPSPSSENANDECFQLLTLLQPTLVEKLLVRDVLDKCVAEELLTVEDRSRVSAAEKNG 159

Lbat 80 VEALRRAGNPLAARYMDPELRDLPSPSSENANDECFQLLTLLQPTLVERLLVRDVLDRCVAVELLTVEDRSRISAAEKNG 159

Dog 81 LVALQSAGSVLASRYLNPELADLPSPSAENAHDQCLQLLNLLQPTLVDRLLVKDVLDKCVEKKLLTDEDRDRISAAENNG 160

Fox 81 LVALERAGSVLASRYLNPELADLPSPSAENAHDQCLQLLNLLQPTLVDRLLVKDVLDKCVEKKLLTDEDRNRISAAENNG 160

Rdog 81 LVALESAGSVLASRYLNPELADLPSASAENAHDQCLQLLNLLQPTLVDRLLVKDVLDKCVEKKLLTDEDRNRISAAENNG 160

Cpang 80 VEALERTGSRLAARCVNPELX---SPSFGNTHDECPQLMSRLQPTLVDTLEVRDVVDKRAEEDLXATEDRNRIVAAENIG 156

Mpang 81 VEALERTGSRSAARCVNPELX---SPSFANTHDECPQLMSRLQPTLVDTLEVRDVVDRCVEEDLXATEDRNRIVAAENIG 157

Human160 NESGVRELLKRIVQKENWFSAFLNVLRQTGNNELVQELTGSDCSESNAEIENLSQVDGPQVEEQLLSTTVQPNLEKEVWG 239

Bbat 160 NEAGVRELLRRIVQKENWFSAFVTVLRQTENEALAQELTGTD---SNAENENLSQEDGPEDKEPPLLATDQSSPEKDAWD 236

Lbat 160 NEAGVRELLRRIVQKENWFSAFVTVLRQTENEALAQELTGTD---SNAGSENSSQEDSPEEKEPLLLATDQSSPEKDAWD 236

Dog 161 NQSGVRELLKRIVQKENWFSLFLTVLNQTENYALVQELTGTTCFESKEETENLSQEDGPEVKAAALLAMSQPSPEKEGWD 240

Fox 161 NQSGVRELLKRIVQKENWFSLFLTVLNQTENYALVQELTGTTCFESKEGTENLSQEDGPEVKAAALLAMSQPSPDKEGWD 240

Rdog 161 NQSGVRELLKRIVQKENWFSLFLTVLNQTENYALVQELTGTTCFESKEGTENLSQEDGPEVKAAALLAVSQPSPEKEGWD 240

Cpang157 NESGIRELLKRIMEKQNWFSTFLTILHfXTGYDGLVQELTGPSCFESNETENLSQ-DGPEVKESHLLATVQPSPEKEAW 235

Mpang158 NESGIRELLKRIMEKQNWFSTFLTILH-STGYDGLVQELTAPVALK--ETENLTQ-DGPEVKESHLLAAVQPSPEKEAW 232

MDA5 Level 2 cd15807 900-1015

Human875 ELQMQSIMEKKMKTKRNIAKHYKNNPSLITFLCKNCSVLACSGEDIHVIEKMHHVNMTPEFKELYIVRENKALQKKCADY 954

Badgr875 ELQMQSIMEKKMKIKRSIAKCYKENPSLINFLCKNCGELACSGEDIHVIEKMHHVNMTPVFKELYTVRENKALQKKFVDY 954

Cpang863 EVQMRSIMEKKIKIKRSMAKQYRDKPSLISCLCKICSVLTXSSEGIHIIERMHQSHMTAEFKELYVVRENKALKKKFAHC 942

Mpang856 EVYMQSIMEKKIKIKRSRAKQYRDKPSLISRLCKLCSVLACSSEXYHVIERMRQGHVTAEFKELCLVRENKALIKKFAHY 935

Human955 QINGEIIC-KCGQAWGTMMVHKGLDLPCLKIRNFVVVFKNNSTKKQYKKWVELPITFPNLDYSECCLFSDED 1025

Badgr955 QTNGEIICKKCGQAWGTMMVHKGLDLPCLKIKNFVVVFKNNMLKKQYKKWVELPITFPDLDYTQYCLFSDED 1026

Cpang943 QANGEIICTKCRRAWGTMMVHKGLDLPFLKXKNFVMVFKNNKPKKQYKKRVELPVIFPGLEYSLYCLLSDED 1014

Mpang936 QANGKIICTKCGQAWGTMMVHKGLDLPCLKXKNFVMVFKNNKPKKQYKKWVEFPVTFPGLEYSLYCLLSDED 1007

NEMO no changes Level 1. Level 2 changes in cd09803:

NEMO cd09803 258-344. Prairie deer mouse, golden hamster, and Norway rat extrapolated to similar identity/positives.

Mustelids corrected:

Human [NP_001093327.1](https://www.ncbi.nlm.nih.gov/protein/NP_001093327.1?report=genbank&log$=protalign&blast_rank=0&RID=BDGFJ7TV016) Ferret [XP_012905560.1](https://www.ncbi.nlm.nih.gov/protein/XP_012905560.1?report=genbank&log$=protalign&blast_rank=1&RID=BDGFJ7TV016) Mink [XP_044091753.1](https://www.ncbi.nlm.nih.gov/protein/XP_044091753.1?report=genbank&log$=protalign&blast_rank=2&RID=BDGFJ7TV016) Eurobadger [XP_045850813.1](https://www.ncbi.nlm.nih.gov/protein/XP_045850813.1?report=genbank&log$=protalign&blast_rank=3&RID=BDGFJ7TV016)

Human241 YDNHIKSSV[7]GMQLEDLKQQLQQAEEALVAKQEVIDKLKEEAEQHKIVMETVPVLKAQADIYKADFQAERQAREKLAE 324 Feret241 YDSHVKSST GMQLEDLKQQLQQAEEALAAKQEVIDKLKEEAEQHKAVMETVPVLKAQADIYKADFQAERQAREKLAE 317

Mink 241 YDSHVKSST GMQLEDLKQQLQQAEEALAAKQEVIDKLKEEAEQHKAVMETVPVLKAQADIYKADFQAERQAREKLAE 317

Badgr241 YDSHVKSST GMQLEDLKQQLQQAEEALAAKQEVIDKLKEEAEQHKAVMETVPVLKAQADIYKADFQAERQAREKLAE 317

Mouse 251 MQLEDLRQQLQQAEEALVAKQELIDKLKEEAEQHKIVMETVPVLKAQADIYKADFQAERHAREKLVE

Human325 KKELLQEQLEQLQREYSKLK 344

Feret318 RKELLQEQLEQLQREYSRLK

Mink 318 RKELLQEQLEQLQREYGRLK

Badgr318 RKELLQEQLEQLQREYGRLK

Mouse KKEYLQEQLEQLQREFNKLK 337

POLA Level 1 and 2

White-tailed deer predicted in two parts; SeqAPASS hit was the C-terminus. Corrections in Level 1 and Level 2 cd05776 (535-767) across the two parts. Result similar to cattle.

Human [NP_058633.2](https://www.ncbi.nlm.nih.gov/protein/NP_058633.2?report=genbank&log$=protalign&blast_rank=0&RID=C31FF517016) Cattle [NP_001192994.1](https://www.ncbi.nlm.nih.gov/protein/NP_001192994.1?report=genbank&log$=protalign&blast_rank=1&RID=C31FF517016) White-tailed deer N-terminus [XP_020758716.1](https://www.ncbi.nlm.nih.gov/protein/XP_020758716.1?report=genbank&log$=protalign&blast_rank=2&RID=C31FF517016)

Human 1 MAPVHGDDSLSDSGSFVSSRARREKKSKKGRQEALERLKKAKAGEKYKYEVEDFTGVYEEVDEEQYSKLVQARQDDDWIV 80

Cattl 1 MAPLHGEDSVSDSRSLVASRSRREKKSKKGRQQALERLKRAKAGEKYKYEVEDFTSVYEEVDEEQYSKLVQARQDDDWIV 80

DeerN 1 ---------MSDSRSFVASRSRREKKSKKGRQEALERLKRAKAGEKYKYEVEDFTSVYEEVDEEQYSKLVQARQDDDWIV 71

Human 81 DDDGIGYVEDGREIFDDDLEDDALDADEKGKDGKARNKDKRNVKKLAVTKPNNIKSMFIACAGKKTADKAVDLSKDGLLG 160

Cattl 81 DDDGIGYVEDGREIFDDDLEDDALDSHEKGKDNKACNKDKRTVKRAAVTKPNNIKSMFIASAGRKTVDKAVDLSKDDLLG 160

DeerN 72 DDDGIGYVEDGREIFDDDLEDDALDSHEKGKDDKACNKDKRTIKRAAVTKPNNIKSMFIASAGRKTVDKAVDLSKDDLLG 151

161 DILQDLNTETPQITPPPVMILKKKRSIGASPNPFSVHTATAVPSGKIASPVSRKEPPLTPVPLKRAEFAGDDVQVESTEE 240

161 DILQDLNTETPQITPPPVIIPKKKRSSGASLNPFSVHTPKAAPSGKTTPPASRREPSLTPAPLTCAEFAGEPGPPLCTDD 240

152 DILQDLNTETPQITPPPVIIPKKKRSSGASLNPFSVHTPKAAPSGKTAPPASTKEPSLTPVPLTCVEFAGEPRMPLCSDD 231

241 EQESGAMEFEDGDFDEPMEVEEVDLEPMAAKAWDKESEPAEEVKQEADSGKGTVSYLGSFLPDVSCWDIDQEGDSSFSVQ 320

241 KEESGAMEFEDGDFDEPMEAEEVDVEPVAAKTLCQEREPAEEAKHKADSGKGTTSSSASFLPDVSCWDMDQEDDSSFSAL 320

232 KEESGAMEFEDGDFDEPMEAEEVDMEPVAAKTLCQEREPAEEAKHEADSGKGTTSSSASFLPDISCWDMDQEDDSSFSAP 311

321 EVQVDSSHLPLVKGADEEQVFHFYWLDAYEDQYNQPGVVFLFGKVWIESAETHVSCCVMVKNIERTLYFLPREMKIDLNT 400

321 EVQVDSSHLPLVKGADEEQVFQFYWLDAYEDPYSQPGVVFLFGKVWIESAETHVSCCVMVKNIERSLCFLPREMKVDLNT 400

312 EVQVDSSHLPLVKGADEEQVFQFYWLDAYEDPYSQPGVVFLFGKVWIESADTHVSCCVMVKNIERTLCFLPREMKVDLNT 391

401 GKETGTPISMKDVYEEFDEKIATKYKIMKFKSKPVEKNYAFEIPDVPEKSEYLEVKYSAEMPQLPQDLKGETFSHVFGTN 480

401 GKESGTPVTMKDVYDEFDEKIAAKYKIMKFKSKVVEKNYAFEIPDVPEKSEYLEVRYSAEMPQLPQDLKGETFSHVFGTN 480

392 GKESGTPVTMKDVYDEFDEKIAAKYKIMKFKSKVVEKNYAFEIPDVPEKSEYLEVRYSAEMTQLPQDLKGETFSHVFGTN 471

481 TSSLELFLMNRKIKGPCWLEVKSPQLLNQPVSWCKVEAMALKPDLVNVIKDVSPPPLVVMAFSMKTMQNAKNHQNEIIAM 560

481 TSSLELFLMNRKIKGPCWLEVKNPQLLNQPISWCKVEAMVLKPDLVNVIKDVGPPPVVVMSLSMKTMQNAKTHENEIIAV 560

472 TSSLELFLMNRKIKGPCWLEVKNPQLLNQPISWCKVEAMVLKPDLVDVIKDVGPPPVVVMSLSMKTMQNAKTHENEIIAV 551

561 AALVHHSFALDKAAPKPPFQSHFCVVSKPKDCIFPYAFKEVIEKKNVKVEVAATERTLLGFFLAKVHKIDPDIIVGHNIY 640

561 SALVHHSFALDKAPPQPPFQSHFCVVSKPKDCIFPYDFKESIKEKNVKVEVAATERTLLGFFLAKVHKIDPDIIVGHNIY 640

552 SALVHHSFALDKAPPQPPFQSHFC-------------------------------------------------------- 575

**POLA**

Human [NP_058633.2](https://www.ncbi.nlm.nih.gov/protein/NP_058633.2?report=genbank&log$=protalign&blast_rank=0&RID=C31FF517016) Cattle [NP_001192994.1](https://www.ncbi.nlm.nih.gov/protein/NP_001192994.1?report=genbank&log$=protalign&blast_rank=1&RID=C31FF517016) White-tailed deer C-term [XP_020742025.1](https://www.ncbi.nlm.nih.gov/protein/XP_020742025.1?report=genbank&log$=protalign&blast_rank=2&RID=C31FF517016)

Human561 AALVHHSFALDKAAPKPPFQSHFCVVSKPKDCIFPYAFKEVIEKKNVKVEVAATERTLLGFFLAKVHKIDPDIIVGHNIY 640

Cattl561 SALVHHSFALDKAPPQPPFQSHFCVVSKPKDCIFPYDFKESIKEKNVKVEVAATERTLLGFFLAKVHKIDPDIIVGHNIY 640

DeerC 1 -------------------------VSKPKDCIFPYDFKESIKEKNVKVEVAATERTLLGFVLAKVHKVDPDIIVGHNIY 55

Human641 GFELEVLLQRINVCKAPHWSKIGRLKRSNMPKLGGRSGFGERNATCGRMICDVEISAKELIRCKSYHLSELVQQILKTER 720

Cattl641 GFELEVLLQRINVCKVPFWSKIGRLKRSNMPKLGGRSGFGEKNATCGRMICDVEISAKELIRCKSYHLSELVQQILKTER 720

DeerC 56 GFELEVLLQRINVCKVPFWSKIGRLKRSNMPKLGGRSGFGERNATCGRMICDVEISAKELIRCKSYHLSELVQQILKTER 135

Human721 VVIPMENIQNMYSESSQLLYLLEHTWKDAKFILQIMCELNVLPLALQITNIAGNIMSRTLMGGRSERNEFLLLHAFYENN 800

Cattl721 IVIPIETIRNMYSDSSHLLYLLEHTWKDARFILQIMCELNVLPLALQITNIAGNIMSRTLMGGRSERNEFLLLHAFYENN 800

DeerC136 IVIPIENIRNMYSDSSHLLYLLEHTWKDARFILQIMCELNVLPLALQITNIAGNIMSRTLMGGRSERNEFLLLHAFYENN 215

801 YIVPDKQIFRKPQQKLGDEDEEIDGDTNKYKKGRKKAAYAGGLVLDPKVGFYDKFILLLDFNSLYPSIIQEFNICFTTVQ 880

801 YIVPDKQTFRKPQQKLGDEDEDIDGDTNKYKKGRKKAAYSGGLVLDPKVGFYDKFILLLDFNSLYPSIIQEFNICFTTVQ 880

216 YIVPDKQTFRKPQQKLGDEDEDIDGDTNKYKKGRKKAAYSGGLVLDPKVGFYDKFILLLDFNSLYPSIIQEFNICFTTVQ 295

881 RVASEAQKVTEDGEQEQIPELPDPSLEMGILPREIRKLVERRKQVKQLMKQQDLNPDLILQYDIRQKALKLTANSMYGCL 960

881 RVASEAQKVVEDGEQEQIPELPDPSLEMGILPREIRKLVERRRQVKQLMKQQDLNPDLHLQYDIRQKALKLTANSMYGCL 960

296 RVASEAQRFAEDGEQEQIPELPDPSLEMGILPREIRKLVERRRHVKQLMKQQDLNPDLYLQYDIRQKALKLTANSMYGCL 375

961 GFSYSRFYAKPLAALVTYKGREILMHTKEMVQKMNLEVIYGDTDSIMINTNSTNLEEVFKLGNKVKSEVNKLYKLLEIDI 1040

961 GFSYSRFYAKPLAALVTYKGREILMHTKEMVQKMNLEVIYGDTDSIMINTNSTNLEEVFKLGNKVKSEVNKLYKLLEIDI 1040

376 GFSYSRFYAKPLAALVTYKGREILMHTKEMVQKMNLEVIYGDTDSIMINTNSTNLEEVFKLGNKVKSEVNKLYKLLEIDI 455

1041 DGVFKSLLLLKKKKYAALVVEPTSDGNYVTKQELKGLDIVRRDWCDLAKDTGNFVIGQILSDQSRDTIVENIQKRLIEIG 1120

1041 DGIFKSLLLLKKKKYAALIVEPTSDGNYVTKQEVKGLDIVRRDWCDLAKDTGNFVIGQILSDQNRDTIVENIQKRLIEIG 1120

456 DGIFKSLLLLKKKKYAALIVEPTSDGNYVTKQEVKGLDIVRRDWCDLAKDTGNFVIGQILSDQSRDTIVENIQKRLIEIG 535

1121 ENVLNGSVPVSQFEINKALTKDPQDYPDKKSLPHVHVALWINSQGGRKVKAGDTVSYVICQDGSNLTASQRAYAPEQLQK 1200

1121 ENVLNGSVPVSQFEINKALTKDPQDYPDKKSLPHVHVALWINSQGGRKVKAGDTVSYVICQDGSNLTAGQRAYAPEQLQK 1200

536 ENVLNGSVPVSQFEINKALTKDPQDYPDKKSLPHVHVALWINSQGGRKVKAGDTVSYIICQDGSNLTAGQRAYAPEQLQK 615

1201 QDNLTIDTQYYLAQQIHPVVARICEPIDGIDAVLIATWLGLDPTQFRVHHYHKDEENDALLGGPAQLTDEEKYRDCERFK 1280

1201 QDNLSIDTQYYLAQQIHPVVARICEPIDGIDAILIASWLGLDPTQFRVHHYHKDEENDALLGGPAHLTDEEKYKDCERFK 1280

616 QDNLNIDTQYYLAQQIHPVVARICEPIDGIDAILIASWLGLDPTQFRVHHYHKDEENDALLGGPAHLTDEEKYKDCERFK 695

1281 CPCPTCGTENIYDNVFDGSGTDMEPSLYRCSNIDCKASPLTFTVQLSNKLIMDIRRFIKKYYDGWLICEEPTCRNRTRHL 1360

1281 CPCPTCGTENIYDSVLDGSGTDMEPSLYRCSNINCKASPLTFMVQLSNKLIMDIRRCIKKYYEGWLICEEPTCRNRTRHL 1360

696 CPCPTCGTENIYDSVFDGSGTDMEPSLYRCSNINCKASPLTFMVQLSNKLIMDIRRCIKKYYEGWLICEEPTCRNRTRHL 775

1361 PLQFSRTGPLCPACMKATLQPEYSDKSLYTQLCFYRYIFDAECALEKLTTDHEKDKLKKQFFTPKVLQDYRKLKNTAEQF 1440

1361 PLQFSRNGPLCQVCMKATLRLEYSDKSLYTQLCFYRYIFDAECALEKLITDHEKDKLKKQFFTPKVLQDYRKLKNTAEQF 1440

776 PLQFSRNGPLCQVCMKATLRLEYSDKSLYTQLCFYRYIFDVECALEKLITDHEKDKLKRQFLTPKVLQDYRKLKNTAEQF 855

1441 LSRSGYSEVNLSKLFAGCAVKS 1462

1441 LSQSGYSEVNLSKLFADCAVWS 1462

856 LSQSGYSEVNLSKLFADCAVWS 877

RAE1 no changes Level 1.

RIG-I Level 2 cd08816 anomalous results for lynx, pig, and squirrel (residues 2-92)

Canada lynx [XP_030149878.1](https://www.ncbi.nlm.nih.gov/protein/XP_030149878.1?report=genbank&log$=protalign&blast_rank=0&RID=B34KSAHH016) Bobcat [XP_046921150.1](https://www.ncbi.nlm.nih.gov/protein/XP_046921150.1?report=genbank&log$=protalign&blast_rank=1&RID=B34KSAHH016) Cat [XP_006939261.1](https://www.ncbi.nlm.nih.gov/protein/XP_006939261.1?report=genbank&log$=protalign&blast_rank=2&RID=B34KSAHH016) Human [NP_055129.2](https://www.ncbi.nlm.nih.gov/protein/NP_055129.2?report=genbank&log$=protalign&blast_rank=3&RID=B34KSAHH016) Pig [AWH63111.1](https://www.ncbi.nlm.nih.gov/protein/AWH63111.1?report=genbank&log$=protalign&blast_rank=4&RID=B34KSAHH016) Gray squirrel [MBZ3879961.1](https://www.ncbi.nlm.nih.gov/protein/MBZ3879961.1?report=genbank&log$=protalign&blast_rank=5&RID=B34KSAHH016)

CLynx 1 MTAEERRNLRAFQDYVIKILDPTYILSYMSPWFKDDEVQYIRAEKNNKGTMEAASLFLKFLLELEEEGWFRGFLDALAHA 80 Bobcat 1 MTAEERRNLRAFQDYVIKILDPTYILSYMSPWFKDDEVQYIRAEKNNKGTMEAASLFLKFLLELQEEGWFRGFLDALAHA 80

Cat 1 MTAEERRNLRAFQDYVIKILDPTYILSYMSPWFKDDEVQYIWAEKNNKGTMEAASLFLKFLLELQEEGWFRGFLDALAHA 80

Human 1 MTTEQRRSLQAFQDYIRKTLDPTYILSYMAPWFREEEVQYIQAEKNNKGPMEAATLFLKFLLELQEEGWFRGFLDALDHA 80

Pig 1 MTAEQRRNLHAFGDYVRKTLDPTFILSYMAPWFRDDEVQHIQAEKNNKGPTEAASLFLQFLLELQEEGWFRGFLDALNQA 80 Squirrl1 MTAEQRRNLLVFRDYVRKILDPTYILGYMAPWFREDKMQHIQAEKNNRGPMEAASLFLEFLLELQEEGWFRGFLDALNQA 80

Clynx 81 GYSGLYEAIESWDFQTIERLEEYRSLLKRLQPEFKTTVNPKDILPEVSKCLISQECEEIMQVCSNKGLMAGAEKMVECFL 160

Bobcat81 GYSGLYEAIESWDFQTIERLEEYRSLLKRLQPEFKTTVNPKDILPEVSKCLISQECEEIMQVCSNKGLMAGAEKMVECFL 160

Cat 81 GYSGLYEAIESWDFQTIERLEEYRSLLKRLQPEFKTTVNPKDILPEVSKCLISQECEEIMQVCSNKGLMAGAEKMVECFL 160

Human 81 GYSGLYEAIESWDFKKIEKLEEYRLLLKRLQPEFKTRIIPTDIISDLSECLINQECEEILQICSTKGMMAGAEKLVECLL 160

Pig 81 GYSGLCEAIESWDFQKIEKLEEYRSLLRRLQPEFKTTINPKDILPEIAECLISQECEEILQICSSKGLMAGAEKMVECLL 160

Squirl81 GYSGLYEAIESW

RIG-I Level 2 cd08817 (100-189, 90 aa’s) Dog had the same sequence as raccoon dog. Dog [XP_005626758.1](https://www.ncbi.nlm.nih.gov/protein/XP_005626758.1?report=genbank&log$=protalign&blast_rank=1&RID=CDTBVKBH01N) Raccoon dog [XP_055157752.1](https://www.ncbi.nlm.nih.gov/protein/XP_055157752.1?report=genbank&log$=protalign&blast_rank=2&RID=CDTBVKBH01N)

Human 81 GYSGLYEAIESWDFKKIEKLEEYRLLLKRLQPEFKTRIIPTDIISDLSECLINQECEEILQICSTKGMMAGAEKLVECLL 160

Dog 81 GYSGLYEAIESWNFQKIESLEEYRLLLKRLQPEFKTTVNPNDILPKISECLISQECEEIIQICSNKGLMAGAEKMVECLL 160

Rdog 81 GYSGLYEAIESWNFQKIESLEEYRLLLKRLQPEFKTTVNPNDILPKISECLISQECEEIIQICSNKGLMAGAEKMVECLL 160

Human161 RSDKENWPKTLKLALEKERNKFSELWIVEkgiKDVETEDLED-KMETSDIQIFYQEDPECQNLSENSCPPSEVSDTnlyS 239

Dog 161 RSDKENWPKTLKLALETEESKFSQLWIVD-

Rdog 161 RSDKENWPKTLKLALETEESKFSQLWIVD-

RIG-I Level 2 pfam04851 241-410 anomalous cattle score in SeqAPASS: Hit length should be the same as other species (170) and positives should be 148. Bat 170, 131, 157 80.6% (NCBI score).

Gorilla [XP_018889279.1](https://www.ncbi.nlm.nih.gov/protein/XP_018889279.1?report=genbank&log$=protalign&blast_rank=0&RID=AUTT5XYC013) Cattle [AYD78266.1](https://www.ncbi.nlm.nih.gov/protein/AYD78266.1?report=genbank&log$=protalign&blast_rank=2&RID=AUTT5XYC013) Deer [XP_020734653.1](https://www.ncbi.nlm.nih.gov/protein/XP_020734653.1?report=genbank&log$=protalign&blast_rank=3&RID=AUTT5XYC013) Human [NP_055129.2](https://www.ncbi.nlm.nih.gov/protein/NP_055129.2?report=genbank&log$=protalign&blast_rank=0&RID=DWDGRHYF013) Big Brown Bat [XP_008140227.2](https://www.ncbi.nlm.nih.gov/protein/XP_008140227.2?report=genbank&log$=protalign&blast_rank=1&RID=DWDGRHYF013)

Gorill240PFKPRNYQLELALPAMKGKNTIICAPTGCGKTFVSLLICEHHLKKFPQGQKGKVVFFANQIPVYEQQKSVFSKYFERHGY 319 Baboon240PFKLRNYQLELALPAKKGKNTIICAPTGCGKTLVSVLICEHHLKKFPPGQKGKVVFFANQIPVYEQQESVFSKYFERLGY 319

Cattle238PLKPRNYQLELALPAQKGKNTIICAPTGCGKTFVSLLICEHHLKKFPQGRKGKVVVFAVQVPLYEQQKSVFSEYFERFGY 317 Deer 238PLKPRNYQLELALPAQKGKNTIICAPTGCGKTFVSLLICEHHLKKFPQGRKGKVVFFAVQLPVYEQQKSVFSKHFERLGY 317

Human 240PFKPRNYQLELALPAMKGKNTIICAPTGCGKTFVSLLICEHHLKKFPQGQKGKVVFFANQIPVYEQQKSVFSKYFERHGY 319

BBBat 238PMKPRNYQLELAGPALQGKNTVICAPTGCGKTFVALLICEHHLQKFPQGRKGKVVFFANQLPVYEQQKNVFLKYFERHGY 317

Gorill320RVTGISGATAENVPVEQIVENNDIIILTPQILVNNLKKGTIPSLSIFTLMIFDECHNTSKQHPYNMIMFNYLDQKLGGSS 399

Baboon320RVTGISGATAENVPVEQIVENNDIIILTPQILVNNLKKGTIPSLSVFTLMIFDECHNTSKQHPYNMIMFNYLDQKLGGSS 399

Cattle318KVSGISGETADNISVEQIVENNDIIILTPQILVNSLKDGTIPSLSIFTLMIFDECHNTNKHHPYNMIMFHYLDQKLGGSS 397

Deer 318KVAGISGATADNVSVEQIIENNDIIILTPQILVNSFKNGTVPSLSIFTLMIFDECHNTNKHHPYNMIMFNYLDQKLGGSS 397

Human 320RVTGISGATAENVPVEQIVENNDIIILTPQILVNNLKKGTIPSLSIFTLMIFDECHNTSKQHPYNMIMFNYLDQKLGGSS 399

BBBat 318KIAGVSGATVENISVEQIVENNDIIILTPQILVNSLKNGTIPSLSVFTLMIFDECHNTSKRHPYNVIMFHYLDQKLGGSS 397

Gorill400GPLPQVIGLTASVGVGDAKNTDEALDYICKLCASLDASVIATVKHNLEELEQVVYKPQKFFRKVESRISDKFKYIIAQLM 479

Baboon400GPLPQVIGLTASVGVGDAKDTDEALDYICKLCASLDASVIATVKDNLEELEQVVYKPQKFFRKVESRVSDKFKCIIAQLM 479

Cattle398DSLPQVIGLTASVGVGDAKNTAEATEYICKLCASLDTAVVTTVRDNLEELEEVVYKPQKFFRKVESRTTDRFKRIISQLM 477

Deer 398DSLPQVIGLTASVGVGDAKNTAEATEYICKLCASLDTSVVTTVRDNLEELEEVVYKPQKFFRKVESRTTDRFKCIISQLM 477

Human 400GPLPQVIGLTASVGVGDAKNTDEALDYICKLCASLDASVIATVKHNLEELEQVVYKPQKFFRKVESRISDKFKYIIAQLM 479

BBBat 398DPLPQVIGLTASVGVGDAKNTAEATEYICKLCASLDTSVVATVKDNLEELEEIVYKPQKFFRKVKSRTINRFKCIISELM 477

SRP19 Level 1 Corrections

Human [NP_003126.1](https://www.ncbi.nlm.nih.gov/protein/NP_003126.1?report=genbank&log$=protalign&blast_rank=0&RID=B5PVKGE2013) squirrel [XP_047411934.1](https://www.ncbi.nlm.nih.gov/protein/XP_047411934.1?report=genbank&log$=protalign&blast_rank=1&RID=B5PVKGE2013) (rat) [NP_001099627.1](https://www.ncbi.nlm.nih.gov/protein/NP_001099627.1?report=genbank&log$=protalign&blast_rank=2&RID=B5PVKGE2013) hamster [XP_040614000.1](https://www.ncbi.nlm.nih.gov/protein/XP_040614000.1?report=genbank&log$=protalign&blast_rank=3&RID=B5PVKGE2013) deer [XP_002721616.4](https://www.ncbi.nlm.nih.gov/protein/XP_002721616.4?report=genbank&log$=protalign&blast_rank=4&RID=B5PVKGE2013) Fox XP_025872553.1 (assume missing residues match all other species). No Level 2 domains.

Human 1 MACAAARSPADQDRFICIYPAYLNNKKTIAEGRRIPISKAVENPTATEIQDVCSAVGLNVFLEKNKMYSREWNRDVQYRG 80

Squirl 1 MACAAARSPADQDRFICIYPAYLNNKKTIAEGRRIPISKAVENPTATEIQDVCSAVGLNVFVEKNKMYSREWNRDAQYRG 80

Rat 1 MACAAARSPADQDRFICIYPAYLNNKKTIAEGRRIPINKAVENPTATEIQDVCSAVGLNAFLEKNKMYSREWNRDVQYRG 80

Hamstr 1 MACAAARSPADQDRFICIYPAYLNNKKTIAEGRRIPVSKAVENPTASEIQDVCSAVGLNAFLEKNKMYSREWNRDVQYRG 80

Deer 1 MACAAARSPAEQDRFICIYPAYLNNKKTIAEGRRIPVSKAVENPTATEIQDVCSAVGLNVLLEKNKMYSREWNRDVQYKG 80

Fox 1 MACAAARSPAKQDRFICIYPAYLNNKKTITKGRRIPISKX------TEIQDGCSAFGLNVFLEKNKMYSREWNRDVQYRG 74

Human 81 RVRVQLKQEDGSLCLVQFPSRKSVMLYAAEMIPKLKTRTQKTGGADQSLQQGEGSKKGKGKKKK 144

Squirl81 RVRVQLRQEDGSLCLVQFPSRKSVMLYAAEMIPKLKTRTQKTGGGDPSLQQGEGSKKGKGKKKK 144

Rat 81 RVRVQLKQEDGSLCLVQFPSRKSVMLYVAEMIPKLKTRTQKSGGADPSLQQGEGSKKGKGKKKK 144

Hamstr81 RVRVQLRQEDGSLCLVQFPSRKSVMLYVAEMIPKLKTRTQKTGGADPSLQQGEGSKKGKGKKKK 144

Deer 81 RVRVQLKQEDGSLCLVQFPSRKSVMLYVAEMIPKLKTRTQKAGGGDPSLQQGEGSKKGKGKKKK 144

Fox 75 RVRVQLKQENDSLCLVQFPSLKSVMMYAAQIIPKLKTRTQKTGGGDQSLQQGEGSEKGKGKK-- 136

SRP54 no changes Level 1. No Level 2 domains.

STAT1 Level 1 Raccoon dog is considered same as dog (sequences match except inaccurately predicted probable splice locations).

STAT2 Level 1 House mouse corrected to 72.5% similarity based on NCBI BLAST. Level 2 no changes.

STING no change, Level 1, Level 2.

TBK1 no change, Level 1, Level 2.

TMPRSS2 no change, Level 1, Level 2.

TOMM70 house mouse (mus musculus) listed as mKIAA0719 protein, partial, with accession number BAC65638.1 and is the same as mus musculus mitochondrial import subunit TOM70 reference sequence NP_613065.2, except for mKIAA0719 protein having 15 additional amino acids at the N-terminus. The mouse reference sequence matches that for the human TOMM70 query sequence, NP_055635.3. In Level 3, a probable sequencing error in the olive baboon predicted mRNA sequence, XM_003893859.5, resulted in a frame shift at the C-terminus of protein sequence, XP_ 003893908.2, which we presume to be the same as the other primates.
