# Supplementary material for: In Silico Analysis of Cross-Species Sequence Variability in Host Interferon Antiviral Pathway Proteins and SARS-CoV-2 Susceptibility
Source: Zoonoses. Author manuscript; Available in PMC 2026 Mar 11. (PMC12973230; doi:10.15212/ZOONOSES-2024-0028)
Supplement: SupplementaryFigures [file NIHMS2123055-supplement-SupplementaryFigures.pdf]

COVID-19 Intermediate Host Supplementary Figures

SeqAPASS Visualizations

Proteins investigated included the entry proteins ACE2 and TMPRSS2, exit protein BST-2, and 21 proteins in the interferon-I antiviral pathway with literature-demonstrated protein-protein interactions with SARS-CoV-2 viral proteins (Figure S1). Taxonomic comparisons for Levels 1 and 2 were conducted at the Order level, except where indicated. Primary (whole protein) sequences are aligned in Level 1 boxplots; conserved domains are aligned in Level 2 boxplots. Legends show relative percent similarity for selected species of interest (data for all individual species with available sequences can be found in Supplementary 2). Level 3 heatmaps indicate a total match, partial match (side chain classification match or molecular weight within 30 grams/mole), or not a match (neither sidechain nor molecular weight match) for specific amino acid positions determined as critical for binding or other factors such as viral enzyme cleavage that would disrupt host protein function (see Methods; full Level 3 results can be found in Supplementary File 3).

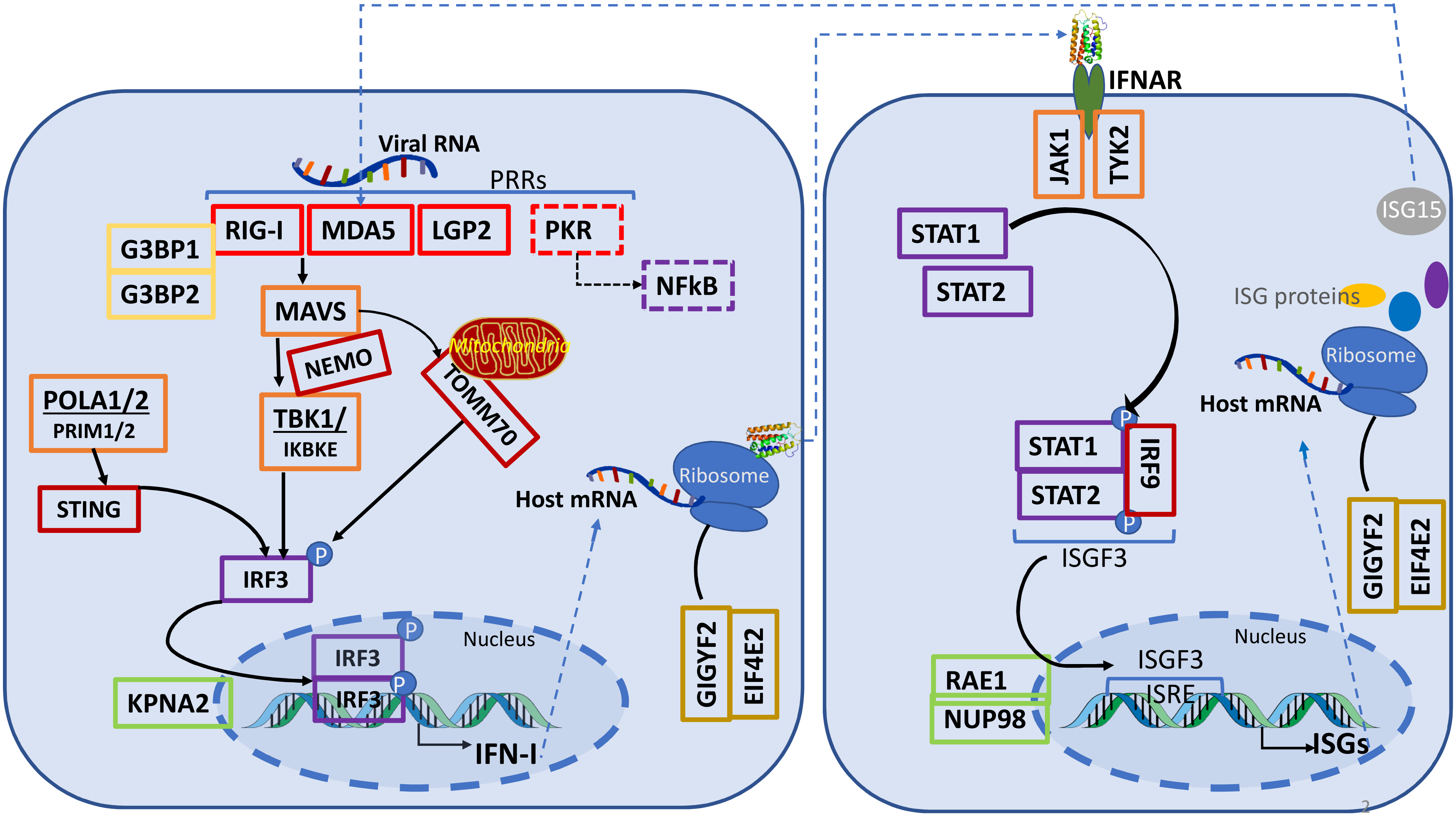

Figure S1a. Interferon-I antiviral response pathway.

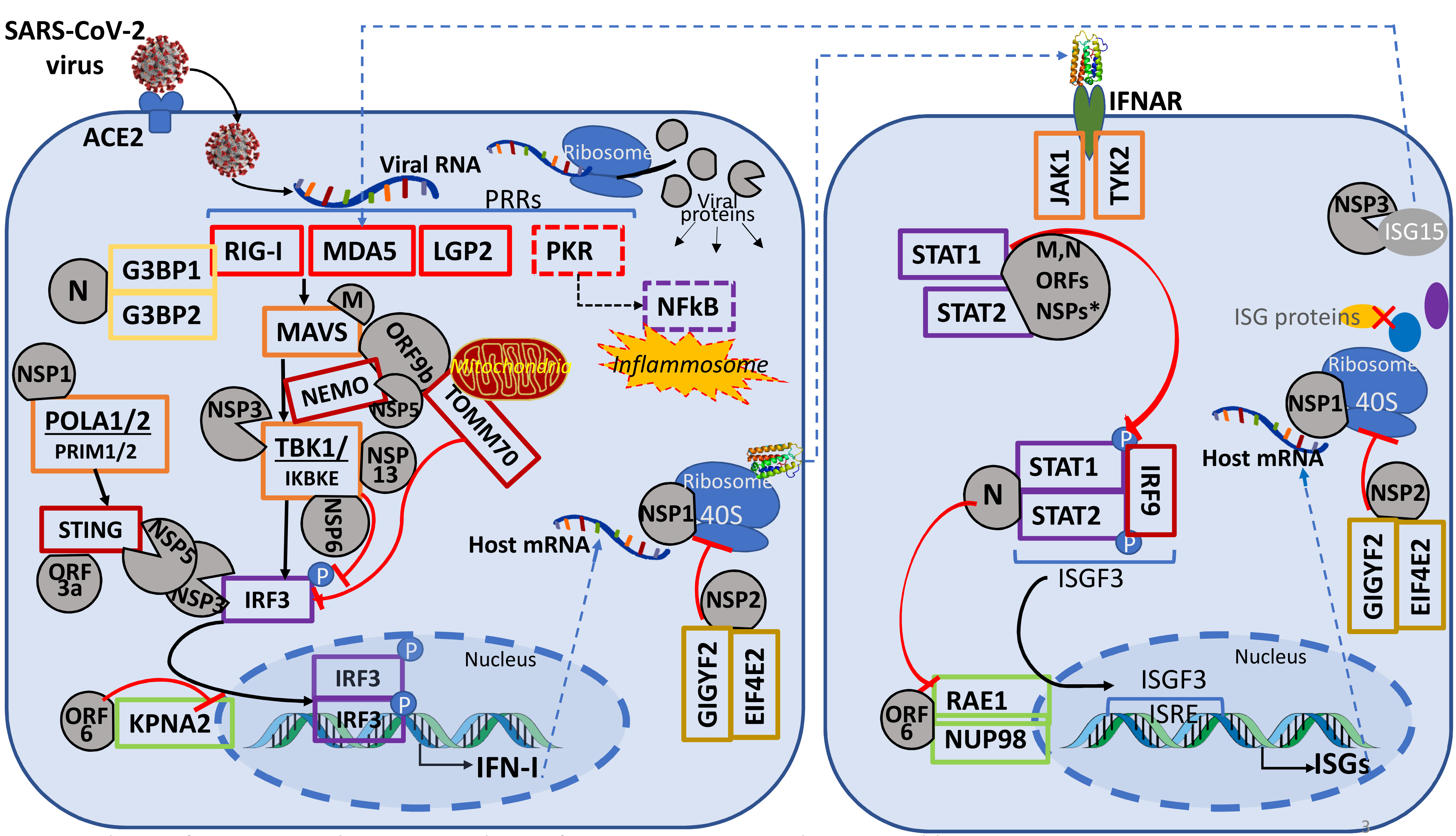

Figure S1b. Interferon-I antiviral response pathway after SARS-CoV-2 entry showing viral-host protein-protein interactions.

Figure S2.
Eukaryotic translation initiation factor 4E type 2 (EIF4E2 or 4EHP)
Query Accession: NP_004837.1
Level 1 only

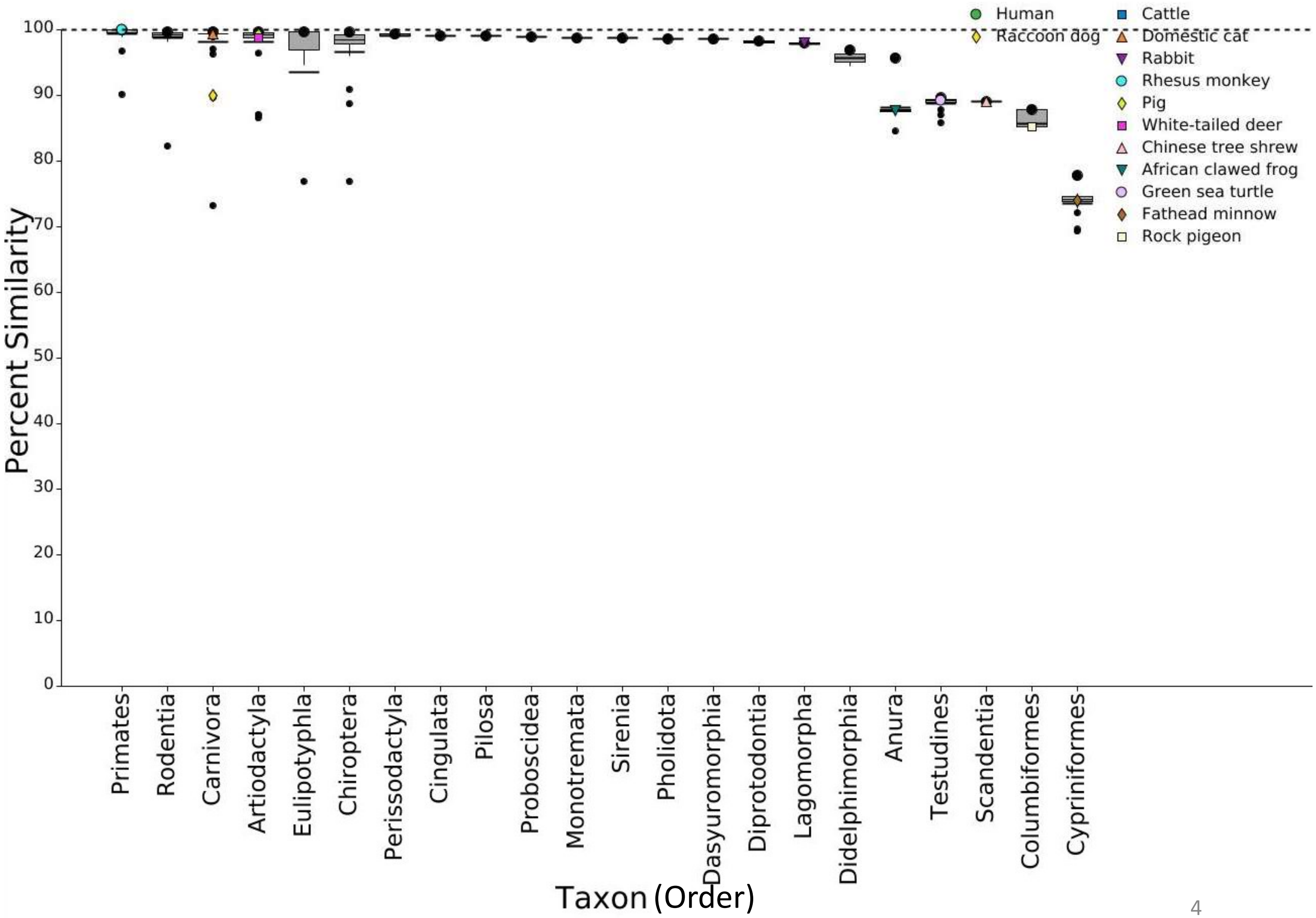

Figure S3a. 40S ribosome S2 (uS5) Query Accession: NP_002943.2 Level 1

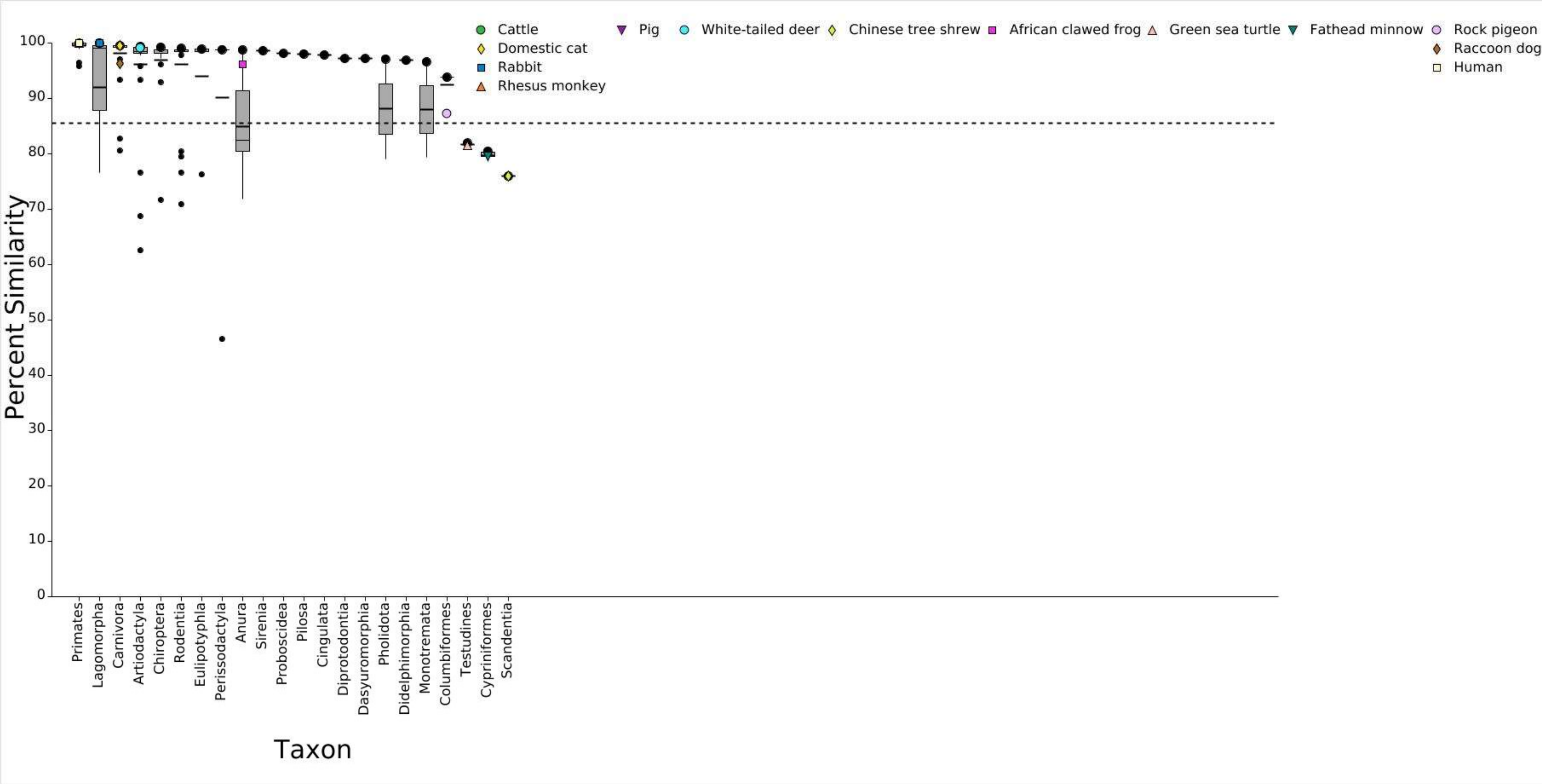

Figure S3b. 40S ribosome S2 (uS5) Query Accession: NP_002943.2. Level3: Bond (Left) and Distance (Right)

Total Match
Partial Match
Not a Match

Common Name	Amino Acid 1	Amino Acid 2	Amino Acid 3
Human	106V	146E	147V
Rhesus monkey	106V	146E	147V
Green monkey	106V	146E	147V
Western lowland gorilla	106V	146E	147V
Olive baboon	106V	146E	147V
Rabbit	106V	146E	147V
White-tufted-ear marmoset	106V	146E	147V
Tiger	106V	146E	147V
Lion	106V	146E	147V
Domestic cat	106V	146E	147V
Bobcat	106V	146E	147V
Canada lynx	106V	146E	147V
Fishing cat	106V	146E	147V
White-tailed deer	106V	146E	147V
Cattle	106V	146E	147V
Dog	106V	146E	147V
American mink	106V	146E	147V
Domestic ferret	106V	146E	147V
Eurasian badger	106V	146E	147V
Gray squirrel	106V	146E	147V
Pig	106V	146E	147V
Prairie deer mouse	106V	146E	147V
Norway rat	106V	146E	147V
Egyptian rousette	106V	146E	147V
House mouse	106V	146E	147V
Golden hamster	106V	146E	147V
Raccoon dog	109V	149E	150V
Red fox	88V	128E	129V
Puma	110V	150E	151V
Malayan pangolin	150V	188E	189V
Chinese tree shrew	72V	112E	113V
Big brown bat	106V	146E	147V
Chinese alligator	52V	92E	93V
Fathead minnow	92V	132E	133V

Total Match
Partial Match
Not a Match

Common Name	Amino Acid 1	Amino Acid 2	Amino Acid 3	Amino Acid 4	Amino Acid 5	Amino Acid 6	Amino Acid 7	Amino Acid 8	Amino Acid 9	Amino Acid 10
Human	105E	107L	109I	111P	113Q	122T	124F	145K	148A	151I
Rhesus monkey	105E	107L	109I	111P	113Q	122T	124F	145K	148A	151I
Green monkey	105E	107L	109I	111P	113Q	122T	124F	145K	148A	151I
Western lowland gorilla	105E	107L	109I	111P	113Q	122T	124F	145K	148A	151I
Olive baboon	105E	107L	109I	111P	113Q	122T	124F	145K	148A	151I
Rabbit	105E	107L	109I	111P	113Q	122T	124F	145K	148A	151I
White-tufted-ear marmoset	105E	107L	109I	111P	113Q	122T	124F	145K	148A	151I
Tiger	105E	107L	109I	111P	113Q	122T	124F	145K	148A	151I
Lion	105E	107L	109I	111P	113Q	122T	124F	145K	148A	151I
Domestic cat	105E	107L	109I	111P	113Q	122T	124F	145K	148A	151I
Bobcat	105E	107L	109I	111P	113Q	122T	124F	145K	148A	151I
Canada lynx	105E	107L	109I	111P	113Q	122T	124F	145K	148A	151I
Fishing cat	105E	107L	109I	111P	113Q	122T	124F	145K	148A	151I
White-tailed deer	105E	107L	109I	111P	113Q	122T	124F	145K	148A	151I
Cattle	105E	107L	109I	111P	113Q	122T	124F	145K	148A	151I
Dog	105E	107L	109I	111P	113Q	122T	124F	145K	148A	151I
American mink	105E	107L	109I	111P	113Q	122T	124F	145K	148A	151I
Domestic ferret	105E	107L	109I	111P	113Q	122T	124F	145K	148A	151I
Eurasian badger	105E	107L	109I	111P	113Q	122T	124F	145K	148A	151I
Gray squirrel	105E	107L	109I	111P	113Q	122T	124F	145K	148A	151I
Pig	105E	107L	109I	111P	113Q	122T	124F	145K	148A	151I
Prairie deer mouse	105E	107L	109I	111P	113Q	122T	124F	145K	148A	151I
Norway rat	105E	107L	109I	111P	113Q	122T	124F	145K	148A	151I
Egyptian rousette	105E	107L	109I	111P	113Q	122T	124F	145K	148A	151I
House mouse	105E	107L	109I	111P	113Q	122T	124F	145K	148A	151I
Golden hamster	105E	107L	109I	111P	113Q	122T	124F	145K	148A	151I
Raccoon dog	108E	110L	112I	114P	116Q	125T	127F	148K	151A	154I
Red fox	87E	89L	91I	93P	95Q	104T	106F	127K	130A	133I
Puma	109E	111L	113I	115P	117Q	126T	128F	149K	152A	155I
Malayan pangolin	149D	151L	153I	155P	157Q	166T	168F	187K	190A	193I
Chinese tree shrew	71E	73L	75T	77P	79Q	88T	90F	111K	114A	117I
Big brown bat	105E	107L	109I	111P	113Q	122T	124F	145K	148A	151I
Chinese alligator	51E	53L	55I	57P	59Q	68T	70F	91K	94A	97I
Fathead minnow	91E	93L	95I	97P	99Q	108T	110F	131K	134A	137I

Figure S4a.
40S ribosome S3 (uS3)
Query Accession: NP_000996.2
Level 1

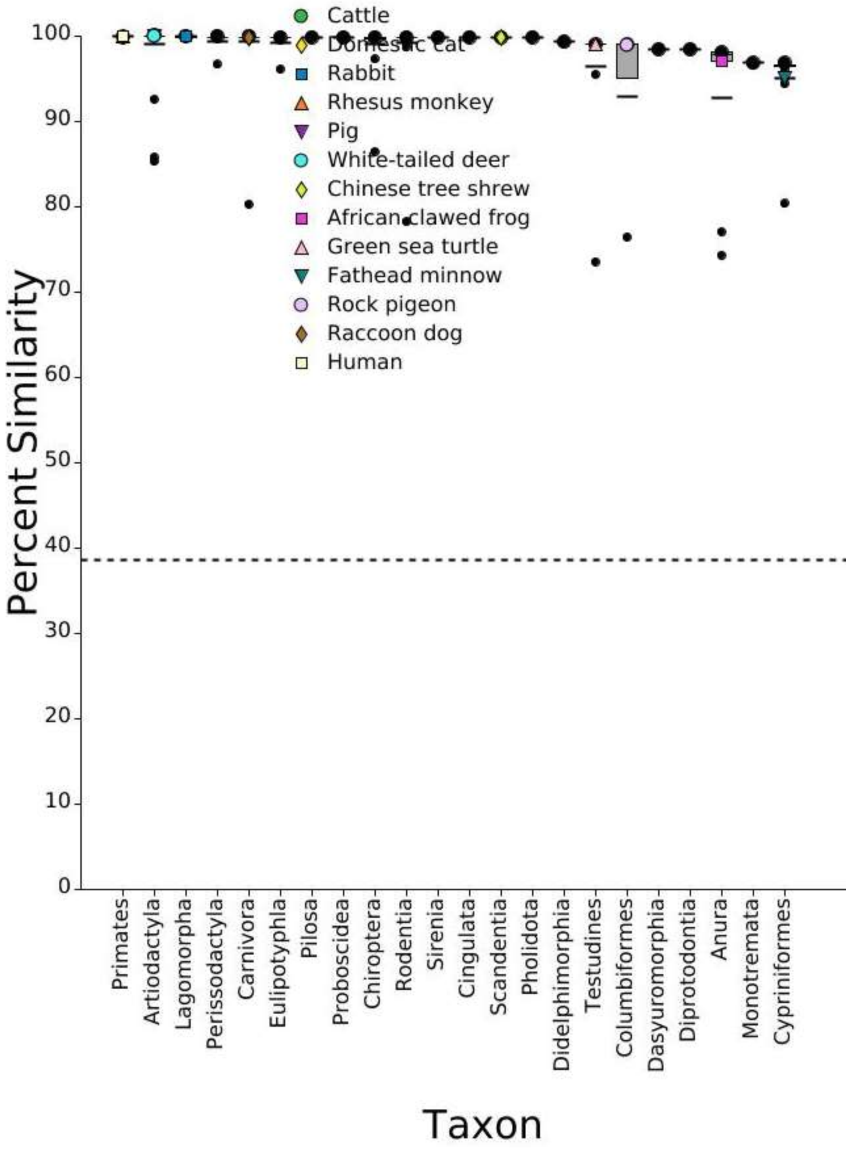

Figure S4b.
40S ribosome S3 (uS3)
Query Accession: NP_000996.2
Level 2: pfam00189 Ribosomal protein S3, C-terminal domain

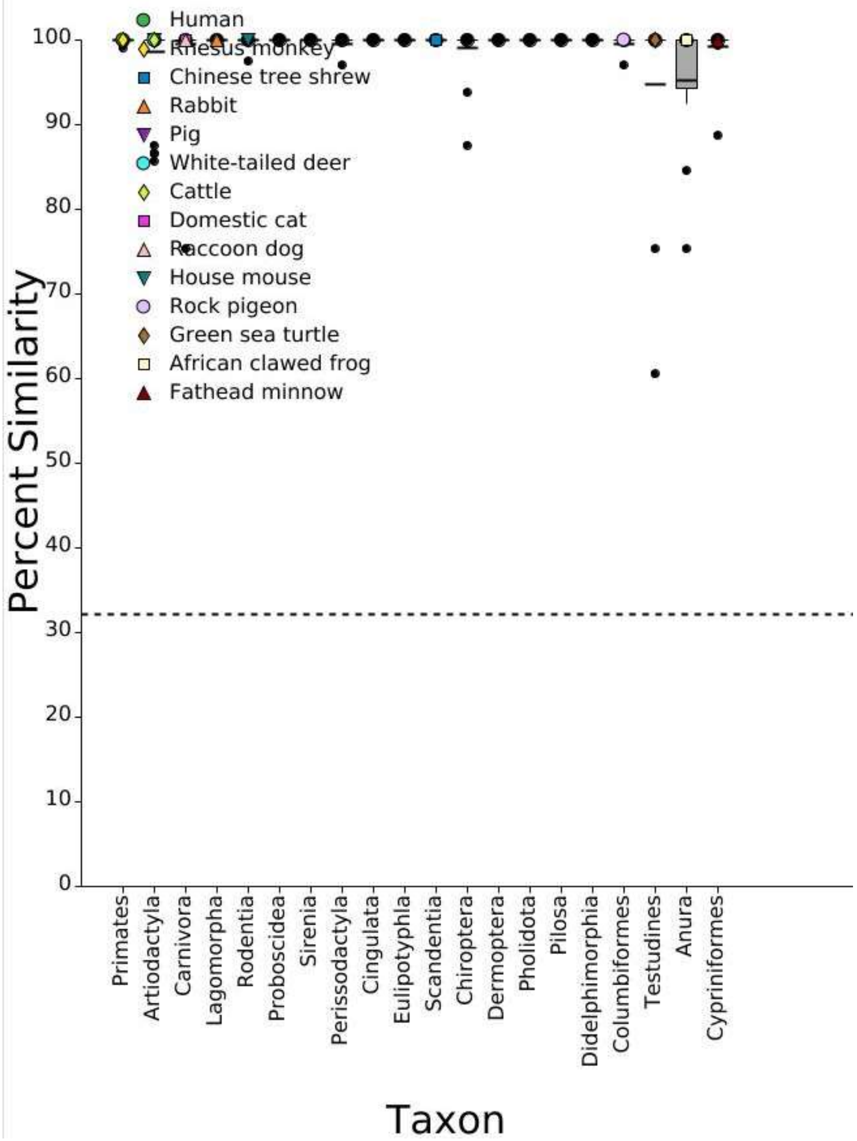

Total Match
Partial Match
Not a Match

Figure S4c. 40S ribosome S3 (uS3) Query Accession: NP_000996.2
Level 3: Bond (Left) and Distance (Right)

Common Name	Amino Acid 1	Amino Acid 2	Amino Acid 3	Amino Acid 4	Amino Acid 5	Amino Acid 6	Amino Acid 7
Human	112G	113L	114A	116R	117R	143R	148K
Rhesus monkey	112G	113L	114A	116R	117R	143R	148K
Green monkey	112G	113L	114A	116R	117R	143R	148K
Western lowland gorilla	112G	113L	114A	116R	117R	143R	148K
Olive baboon	112G	113L	114A	116R	117R	143R	148K
White-tailed deer	112G	113L	114A	116R	117R	143R	148K
Cattle	112G	113L	114A	116R	117R	143R	148K
Rabbit	112G	113L	114A	116R	117R	143R	148K
Dog	112G	113L	114A	116R	117R	143R	148K
Pig	112G	113L	114A	116R	117R	143R	148K
Domestic cat	112G	113L	114A	116R	117R	143R	148K
Golden hamster	112G	113L	114A	116R	117R	143R	148K
Prairie deer mouse	112G	113L	114A	116R	117R	143R	148K
Tiger	112G	113L	114A	116R	117R	143R	148K
Lion	112G	113L	114A	116R	117R	143R	148K
Puma	112G	113L	114A	116R	117R	143R	148K
Raccoon dog	112G	113L	114A	116R	117R	143R	148K
Bobcat	112G	113L	114A	116R	117R	143R	148K
Fishing cat	112G	113L	114A	116R	117R	143R	148K
Red fox	112G	113L	114A	116R	117R	143R	148K
American mink	112G	113L	114A	116R	117R	143R	148K
Chinese tree shrew	112G	113L	114A	116R	117R	143R	148K
Domestic ferret	112G	113L	114A	116R	117R	143R	148K
Eurasian badger	112G	113L	114A	116R	117R	143R	148K
Malayan pangolin	112G	113L	114A	116R	117R	143R	148K
Gray squirrel	112G	113L	114A	116R	117R	143R	148K
Norway rat	112G	113L	114A	116R	117R	143R	148K
Egyptian rousette	112G	113L	114A	116R	117R	143R	148K
House mouse	112G	113L	114A	116R	117R	143R	148K
Canada lynx	112G	113L	114A	116R	117R	143R	148K
White-tufted-ear marmoset	151G	152L	153A	155R	156R	182R	187K
Big brown bat	112G	113L	114A	116R	117R	143R	148K
Rock pigeon	112G	113L	114A	116R	117R	143R	148K
Chinese alligator	112G	113L	114A	116R	117R	143R	148K
African clawed frog	112G	113L	114A	116R	117R	143R	148K
Fathead minnow	112G	113L	114A	116R	117R	143R	148K

Total Match
Partial Match
Not a Match

Common Name	Amino Acid 1	Amino Acid 2	Amino Acid 3	Amino Acid 4
Human	115V	142L	150M	177L
Rhesus monkey	115V	142L	150M	177L
Green monkey	115V	142L	150M	177L
Western lowland gorilla	115V	142L	150M	177L
Olive baboon	115V	142L	150M	177L
White-tailed deer	115V	142L	150M	177L
Cattle	115V	142L	150M	177L
Rabbit	115V	142L	150M	177L
Dog	115V	142L	150M	177L
Pig	115V	142L	150M	177L
Domestic cat	115V	142L	150M	177L
Golden hamster	115V	142L	150M	177L
Prairie deer mouse	115V	142L	150M	177L
Tiger	115V	142L	150M	177L
Lion	115V	142L	150M	177L
Puma	115V	142L	150M	177L
Raccoon dog	115V	142L	150M	177L
Bobcat	115V	142L	150M	177L
Fishing cat	115V	142L	150M	177L
Red fox	115V	142L	150M	177L
American mink	115V	142L	150M	177L
Chinese tree shrew	115V	142L	150M	177L
Domestic ferret	115V	142L	150M	177L
Eurasian badger	115V	142L	150M	177L
Malayan pangolin	115V	142L	150M	177L
Gray squirrel	115V	142L	150M	177L
Norway rat	115V	142L	150M	177L
Egyptian rousette	115V	142L	150M	177L
House mouse	115V	142L	150M	177L
Canada lynx	115V	142L	150M	177L
White-tufted-ear marmoset	154V	181L	189M	216L
Big brown bat	115V	142L	150M	177L
Rock pigeon	115V	142L	150M	177L
Chinese alligator	115V	142L	150M	177L
African clawed frog	115V	142L	150M	177L
Fathead minnow	115V	142L	150M	177L

Figure S5a.
Angiotensin converting enzyme 2 (ACE2)
Query Accession: NP_068576.1
Level 1

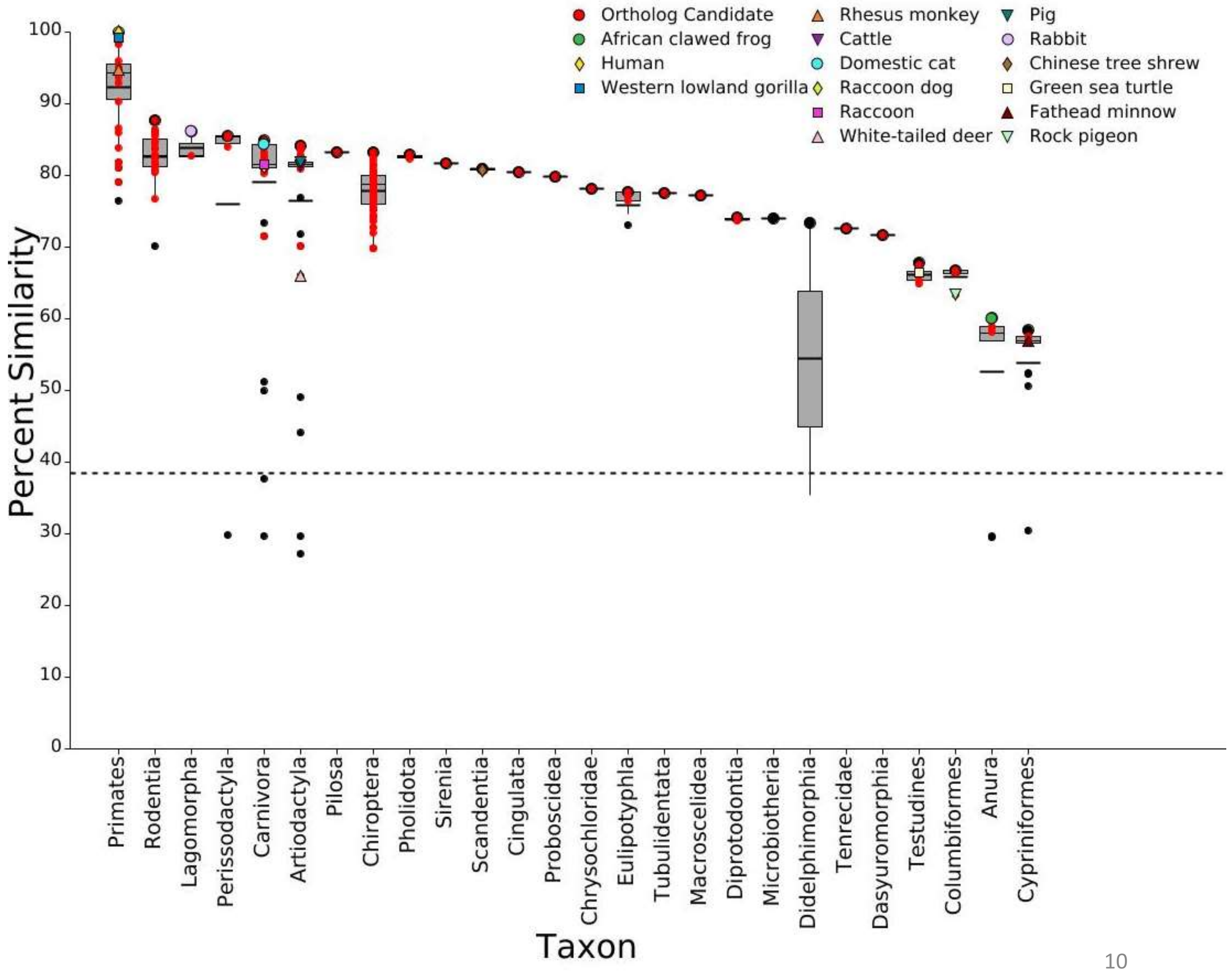

Figure S5b.
Angiotensin converting enzyme 2 (ACE2)
Query Accession: NP_068576.1
Level 3: Bond

(Note bobcat sequence correction, see Supplementary File 4.)

Total Match
Partial Match
Not a Match

Common Name	Amino Acid 1	Amino Acid 2	Amino Acid 3	Amino Acid 4	Amino Acid 5	Amino Acid 6	Amino Acid 7	Amino Acid 8	Amino Acid 9	Amino Acid 10
Human	19S	24Q	30D	31K	35E	38D	41Y	42Q	83Y	353K
Big brown bat	19S	24N	30E	31N	35E	38D	41H	42E	83Y	352K
Western lowland gorilla	19S	24Q	30D	31K	35E	38D	41Y	42Q	83Y	353K
Olive baboon	19S	24Q	30D	31K	35E	38D	41Y	42Q	83Y	353K
Rhesus monkey	19S	24Q	30D	31K	35E	38D	41Y	42Q	83Y	353K
Green monkey	19S	24Q	30D	31K	35E	38D	41Y	42Q	83Y	353K
White-tufted-ear marmoset	19S	24Q	30D	31K	35E	38D	41H	42E	83Y	353K
Gray squirrel	19S	24L	30D	31K	35E	38D	41Y	42Q	83Y	353K
Rabbit	19S	24L	30E	31K	35E	38D	41Y	42Q	83Y	353K
Puma	19S	24L	30E	31K	35E	38E	41Y	42Q	83Y	353K
Tiger	19S	24L	30E	31K	35E	38E	41Y	42Q	83Y	353K
Fishing cat	19S	24L	30E	31K	35E	38E	41Y	42Q	83Y	353K
Domestic cat	19S	24L	30E	31K	35E	38E	41Y	42Q	83Y	353K
Canada lynx	19S	24L	30E	31K	35E	38E	41Y	42Q	83Y	353K
Golden hamster	19S	24Q	30D	31K	35E	38D	41Y	42Q	83Y	353K
Dog	19S	23L	29E	30K	34E	37E	40Y	41Q	82Y	352K
Malayan pangolin	19S	24E	30E	31K	35E	38E	41Y	42Q	83Y	353K
Prairie deer mouse	19S	24Q	30D	31K	35E	38D	41Y	42Q	83Y	353K
Pig	19S	24L	30E	31K	35E	38D	41Y	42Q	83Y	353K
American mink	19S	24L	30E	31K	35E	38E	41Y	42Q	83Y	353K
Eurasian badger	19S	24L	30E	31K	35E	38E	41Y	42Q	83Y	353K
Raccoon	19S	24L	30E	31N	35E	38E	41Y	42Q	83Y	353K
Domestic ferret	19S	24L	30E	31K	35E	38E	41Y	42Q	83Y	353K
Cattle	19S	24Q	30E	31K	35E	38D	41Y	42Q	83Y	352K
House mouse	19S	24N	30N	31N	35E	38D	41Y	42Q	83F	353H
Raccoon dog	19S	23L	29E	30K	34E	37E	40Y	41Q	82Y	352R
Red fox	19S	23L	29E	30K	34E	37E	40Y	41Q	82Y	352K
Norway rat	19S	24K	30N	31K	35E	38D	41Y	42Q	83F	353H
Chinese tree shrew	19T	24E	30N	31K	35E	38E	41H	42Q	83Y	353K
Egyptian rousette	19S	24L	30E	31K	35E	38D	41Y	42Q	83Y	353K
Bobcat	--	--	--	--	--	--	--	--	--	231K
White-tailed deer	19S	24Q	30E	31K	35E	38D	41Y	42Q	83Y	352K
Lion	69V	74E	80E	81E	85R	88V	91N	92E	133F	392N
Chinese alligator	19D	--	25N	26Q	30N	33G	36Y	37E	78Y	348M
African clawed frog	20Q	25Q	31K	32Q	36E	39I	42H	43Q	84F	355M
Fathead minnow	33Q	38G	44K	45K	49D	52N	55Y	56Q	97F	366N

Figure S5b.
Angiotensin converting enzyme 2 (ACE2)
Query Accession: NP_068576.1
Level 3: Distance

(Note bobcat sequence correction, see Supplementary File 4.)

Total Match
Partial Match
Not a Match

Common Name	Amino Acid 1	Amino Acid 2	Amino Acid 3	Amino Acid 4	Amino Acid 5	Amino Acid 6	Amino Acid 7	Amino Acid 8	Amino Acid 9	Amino Acid 10	Amino Acid 11	Amino Acid 12
Human	27T	28F	34H	37E	45L	79L	82M	330N	354G	355D	357R	393R
Big brown bat	27I	28F	34S	37E	45L	79L	82T	329N	353N	354D	356R	392R
Western lowland gorilla	27T	28F	34H	37E	45L	79L	82M	330N	354G	355D	357R	393R
Olive baboon	27T	28F	34H	37E	45L	79L	82M	330N	354G	355D	357R	393R
Rhesus monkey	27T	28F	34H	37E	45L	79L	82M	330N	354G	355D	357R	393R
Green monkey	27T	28F	34H	37E	45L	79L	82M	330N	354G	355D	357R	393R
White-tufted-ear marmoset	27T	28F	34H	37E	45L	79L	82T	330N	354Q	355D	357R	393R
Gray squirrel	27T	28F	34Q	37E	45L	79L	82T	330N	354G	355D	357R	393R
Rabbit	27T	28F	34Q	37E	45L	79L	82T	330N	354G	355D	357R	393R
Puma	27T	28F	34H	37E	45L	79L	82T	330N	354G	355D	357R	393R
Tiger	27T	28F	34H	37E	45L	79L	82T	330N	354G	355D	357R	393R
Fishing cat	27T	28F	34H	37E	45L	79L	82T	330N	354G	355D	357R	393R
Domestic cat	27T	28F	34H	37E	45L	79L	82T	330N	354G	355D	357R	393R
Canada lynx	27T	28F	34H	37E	45L	79L	82T	330N	354G	355D	357R	393R
Golden hamster	27T	28F	34Q	37E	45L	79L	82N	330N	354G	355D	357R	393R
Dog	26T	27F	33Y	36E	44L	78L	81T	329N	353G	354D	356R	392R
Malayan pangolin	27T	28F	34S	37E	45L	79I	82N	330N	354H	355D	357R	393R
Prairie deer mouse	27I	28F	34Q	37E	45L	79L	82N	330N	354G	355D	357R	393R
Pig	27T	28F	34L	37E	45L	79I	82T	330N	354G	355D	357R	393R
American mink	27T	28F	34Y	37E	45L	79H	82T	330N	354H	355D	357R	393R
Eurasian badger	27T	28F	34H	37E	45L	79H	82T	330N	354R	355D	357R	393R
Raccoon	27T	28F	34N	37E	45L	79Q	82T	330N	354G	355D	357R	393R
Domestic ferret	27T	28F	34Y	37E	45L	79H	82T	330N	354R	355D	357R	393R
Cattle	27T	28F	34H	37E	45L	79M	82T	329N	353G	354D	356R	392R
House mouse	27T	28F	34Q	37E	45L	79T	82S	330N	354G	355D	357R	393R
Raccoon dog	26T	27F	33Y	36E	44L	78L	81T	329N	353G	354D	356R	392R
Red fox	26T	27F	33Y	36E	44L	78L	81T	329N	353G	354D	356R	392R
Norway rat	27S	28F	34Q	37E	45L	79I	82N	330N	354G	355D	357R	393R
Chinese tree shrew	27V	28F	34I	37E	45L	79Q	82R	330K	354N	355D	357R	393R
Egyptian rousette	27T	28F	34T	37E	45L	79L	82T	330K	354G	355D	357R	393R
Bobcat	--	--	--	--	--	--	--	208N	232G	233D	235R	271R
White-tailed deer	27T	28F	34H	37E	45L	79M	82T	329N	353G	354D	356R	392R
Lion	77K	78F	84R	87Q	95E	129W	132Q	369K	393G	395D	397R	433R
Chinese alligator	22T	23F	29Q	32E	40L	74N	77K	326N	349K	350D	352R	388R
Fathead minnow	41E	42F	48E	51S	59L	93E	96A	344N	367R	369D	371R	407R
African clawed frog	28D	29F	35L	38E	46L	80A	83A	332N	356N	357D	359R	395R

Figure S5b.
Angiotensin converting enzyme 2 (ACE2)
Query Accession: NP_068576.1
Level 3: N-glycosylation sites

■	Total Match
■	Partial Match
■	Not a Match

Common Name	Amino Acid 1	Amino Acid 2	Amino Acid 3
Human	53N	90N	322N
Western lowland gorilla	53N	90N	322N
Olive baboon	53N	90N	322N
Rhesus monkey	53N	90N	322N
Green monkey	53N	90N	322N
White-tufted-ear marmoset	53N	90N	322N
Gray squirrel	53N	90N	322N
Rabbit	53N	90N	322S
Horse	53N	90N	322N
Masked palm civet	53N	90D	322N
Puma	53N	90N	322N
Tiger	53N	90N	322N
Fishing cat	53N	90N	322N
Domestic cat	53N	90N	322N
Minke whale	53N	90N	321N
Canada lynx	53N	90N	322N
Golden hamster	53N	90N	322Y
Dog	52N	89D	321N
Malayan pangolin	53N	90N	322K
Prairie deer mouse	53N	90N	322H
Pig	53N	90T	322N
American mink	53N	90D	322N
Eurasian badger	53N	90D	322N
Raccoon	53N	90D	322N
Steller sea lion	53N	90D	322N
European mink	53N	90D	322N
Domestic ferret	53N	90D	322N
Cattle	53N	90N	321Y
Raccoon dog	52N	89D	321N
Red fox	52N	89D	321N
Norway rat	53N	90N	322Q
Chinese tree shrew	53N	90D	322N
Big brown bat	53N	90N	321S
Intermediate horseshoe bat	53N	90T	322N
Egyptian rousette	53N	90D	322N
White-tailed deer	53N	90N	321H
Lion	103N	140N	361P
Shrew mouse	53N	90N	322H
Ryukyu mouse	53N	90T	322H
House mouse	53N	90T	322H
Chinese alligator	48N	85D	318P
Rock pigeon	54N	86D	319E
African clawed frog	54N	91D	324A
Fathead minnow	67D	104D	336A

Figure S6a. BST-2 bone marrow stromal antigen 2 (Tethrin) is only found in mammals

Query Accession: NP_004326.1

Level 1

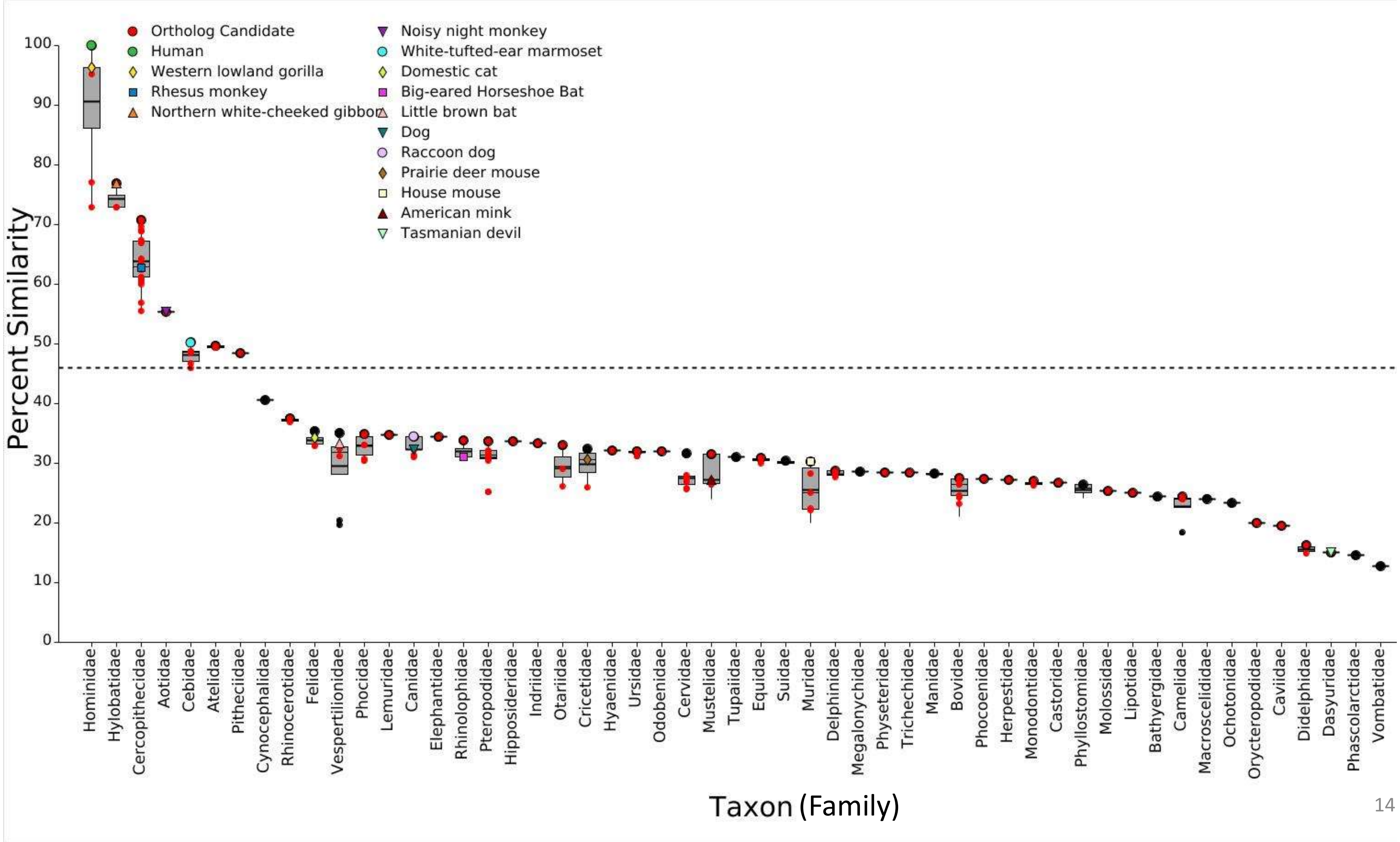

Figure S6b. BST-2 (Tethrin) (found only in mammals)
Template Accession: NP_004326.1
Level 3: predicted binding pocket

Total Match
Partial Match
Not a Match

Common Name	Amino Acid 1	Amino Acid 2	Amino Acid 3	Amino Acid 4	Amino Acid 5	Amino Acid 6	Amino Acid 7	Amino Acid 8	Amino Acid 9	Amino Acid 10	Amino Acid 11	Amino Acid 12	Amino Acid 13	Amino Acid 14	Amino Acid 15	Amino Acid 16	Amino Acid 17	Amino Acid 18	Amino Acid 19	Amino Acid 20	Amino Acid 21
Human	48A	49N	50S	51E	52A	53C	84V	85E	87Q	88A	89A	91C	92N	105E	106K	108Q	109G	110Q	112K	113V	117E
Western lowland gorilla	53A	54N	55S	56E	57A	58C	89V	90E	92Q	93A	94A	96C	97N	110E	111K	113Q	114G	115Q	117K	118V	122E
Green monkey	51A	52N	53S	54E	55A	56C	87A	88E	90Q	91A	92V	94C	95N	108E	109K	111Q	112G	113R	115K	116V	120E
Rhesus monkey	51A	52N	53S	54E	55A	56C	87A	88E	90Q	91A	92V	94C	95N	108E	109K	111Q	112G	113R	115K	116V	120E
Olive baboon	51A	52N	53S	54E	55A	56C	87A	88E	90Q	91A	92V	94C	95N	108E	109K	111Q	112G	113R	115K	116V	120E
White-tufted-ear marmoset	55A	56N	57S	58E	59A	60C	91A	92E	94Q	95I	96A	98Y	99N	112E	113K	115H	116S	120E	122K	123V	127E
Fishing cat	35A	36N	37S	38K	39A	40C	71T	72E	74Q	75A	76A	78C	79N	92E	93K	95Q	96S	100L	102K	103R	107R
Raccoon dog	54A	55N	56S	57K	58A	59C	90T	91M	93Q	94A	95T	97C	98N	111E	112K	114W	115G	119L	121R	122E	126Q
Domestic cat	54A	55N	56S	57K	58A	59C	90N	91E	93Q	94A	95A	97C	98N	111E	112K	114Q	115S	119L	121K	122G	126R
Bobcat	35A	36N	37S	38K	39A	40C	71N	72E	74Q	75A	76A	78C	79N	92E	93K	95Q	96S	100L	102K	103G	107R
Canada lynx	35A	36N	37S	38K	39A	40C	71N	72E	74Q	75A	76A	78C	79N	92E	93K	95Q	96S	100L	102K	103G	107R
Little brown bat	51A	52N	53S	54P	55A	56C	87T	88E	90Q	91A	92A	94C	95N	108E	109K	111A	112G	116Q	118L	119V	123Q
Tiger	35A	36N	37S	38K	39A	40C	71T	72E	74Q	75A	76A	78C	79N	92E	93K	95Q	96S	100L	102K	103R	107R
Dog	55T	56N	57S	58K	59A	60C	91T	92M	94Q	95A	96T	98C	99N	112E	113K	115W	116G	120L	122R	123G	127Q
White-tailed deer	53A	54N	55S	56E	57A	58C	89K	90E	92E	93A	94A	96C	97N	110E	111Q	113Q	--	--	--	114V	118Q
Domestic ferret	53A	54N	55S	56K	57A	58C	89A	90K	92E	93A	94A	96C	97N	110E	111K	113Q	114G	118L	120G	121R	125Q
Red fox	54A	55N	56S	57K	58A	59C	90T	91M	93Q	94A	95T	97C	98N	111E	112K	114W	115G	119L	121R	122G	126Q
Chinese tree shrew	53A	54N	55S	56E	57A	58C	89A	90E	92Q	93A	94A	96C	97N	107E	108K	110E	111K	115Q	117R	118E	122Q
Prairie deer mouse	53A	54N	55S	56E	57A	58C	89A	90E	92Q	93A	94N	96C	97N	110Q	111V	113Q	114V	118Q	120R	121I	--
House mouse	53A	54N	55S	56V	57A	58C	89A	90E	92Q	93A	94N	96C	97N	110K	111V	113Q	114A	118Q	120R	121I	--
Pig	51A	52N	53S	54K	55G	56C	87T	88K	90Q	91Y	92T	94C	95N	108E	109K	111Q	112S	113Q	115Q	116L	120Q
Malayan pangolin	53A	54N	55S	56E	57T	58C	89I	90K	92Q	93A	94A	96C	97N	110E	111K	113H	114S	118Q	120Q	121V	125Q
Cattle	57A	58N	59S	60K	61A	62C	93K	94E	96E	97A	98A	100C	101N	114E	115Q	117Q	--	--	--	118V	122Q
American mink	104A	105N	106S	107K	108A	109C	140A	141K	143E	144A	145A	147C	148N	161E	162K	164Q	165G	169L	171R	172R	176Q
Eurasian badger	53A	54N	55S	56K	57A	58C	89A	90M	92E	93A	94A	96C	97N	110E	111K	113R	114G	118L	120R	121R	125Q
Golden hamster	51A	52S	53S	54E	55A	56C	87A	88E	90Q	91A	92G	94C	95N	108K	109V	111Q	112I	116Q	118R	119I	123Q
Egyptian rousette	54A	55N	56S	57K	58A	59C	90A	91E	93Q	94A	95Y	97C	98N	111E	112K	114E	115S	119R	121L	122A	126Q
Norway rat	53A	54H	55S	56E	57A	58C	89T	90E	92Q	93A	94N	96C	97N	110K	111V	113Q	114T	118Q	120R	121I	--

Total Match
Partial Match
Not a Match

Level 3 binding pocket for bats

Common Name	Amino Acid 1	Amino Acid 2	Amino Acid 3	Amino Acid 4	Amino Acid 5	Amino Acid 6	Amino Acid 7	Amino Acid 8	Amino Acid 9	Amino Acid 10	Amino Acid 11	Amino Acid 12	Amino Acid 13	Amino Acid 14	Amino Acid 15	Amino Acid 16	Amino Acid 17	Amino Acid 18	Amino Acid 19	Amino Acid 20	Amino Acid 21
Human	48A	49N	50S	51E	52A	53C	84V	85E	87Q	88A	89A	91C	92N	105E	106K	108Q	109G	110Q	112K	113V	117E
Greater horseshoe bat	52A	53N	54R	55E	56S	57C	88M	89K	91Q	92T	93A	95C	96N	109E	110K	112Q	113G	114P	116Q	120L	124Q
Little brown bat	51A	52N	53S	54P	55A	56C	87T	88E	90Q	91A	92A	94C	95N	108E	109K	111A	112G	113H	115Q	119V	123Q
Big-eared Horseshoe Bat	52A	53N	54R	55E	56A	57C	88M	89K	91Q	92A	93A	95C	96N	109E	110K	112H	113G	114H	116Q	120V	124Q
Chinese rufous horseshoe bat	52A	53N	54R	55E	56A	57C	88M	89K	91Q	92A	93A	95C	96N	109E	110K	112H	113G	114H	116Q	120V	124Q
Common bats	54A	55N	56S	57P	58A	59C	90T	91E	93Q	94A	95A	97C	98N	111E	112K	114A	115G	116H	118Q	122V	126Q
Jamaican fruit-eating bat	56A	57H	58S	59L	60A	61C	92T	93E	95Q	96A	97A	99Y	100N	113E	114Q	116Q	117G	118Q	120L	124V	128Q
Big brown bat	54D	55N	56S	57P	58A	59C	90T	91E	93Q	94A	95A	97C	98N	111E	112K	114G	115S	116D	118Q	122V	126Q
Common bats	51L	52N	53S	54I	55T	56C	86T	87N	--	--	88A	90C	91N	104E	105Q	107Q	108G	109H	111Q	115V	119Q

Figure S7a.
ras GTPase-activating protein-binding protein 1 (G3BP1)
Query Accession: NP_005745.1
Level 1

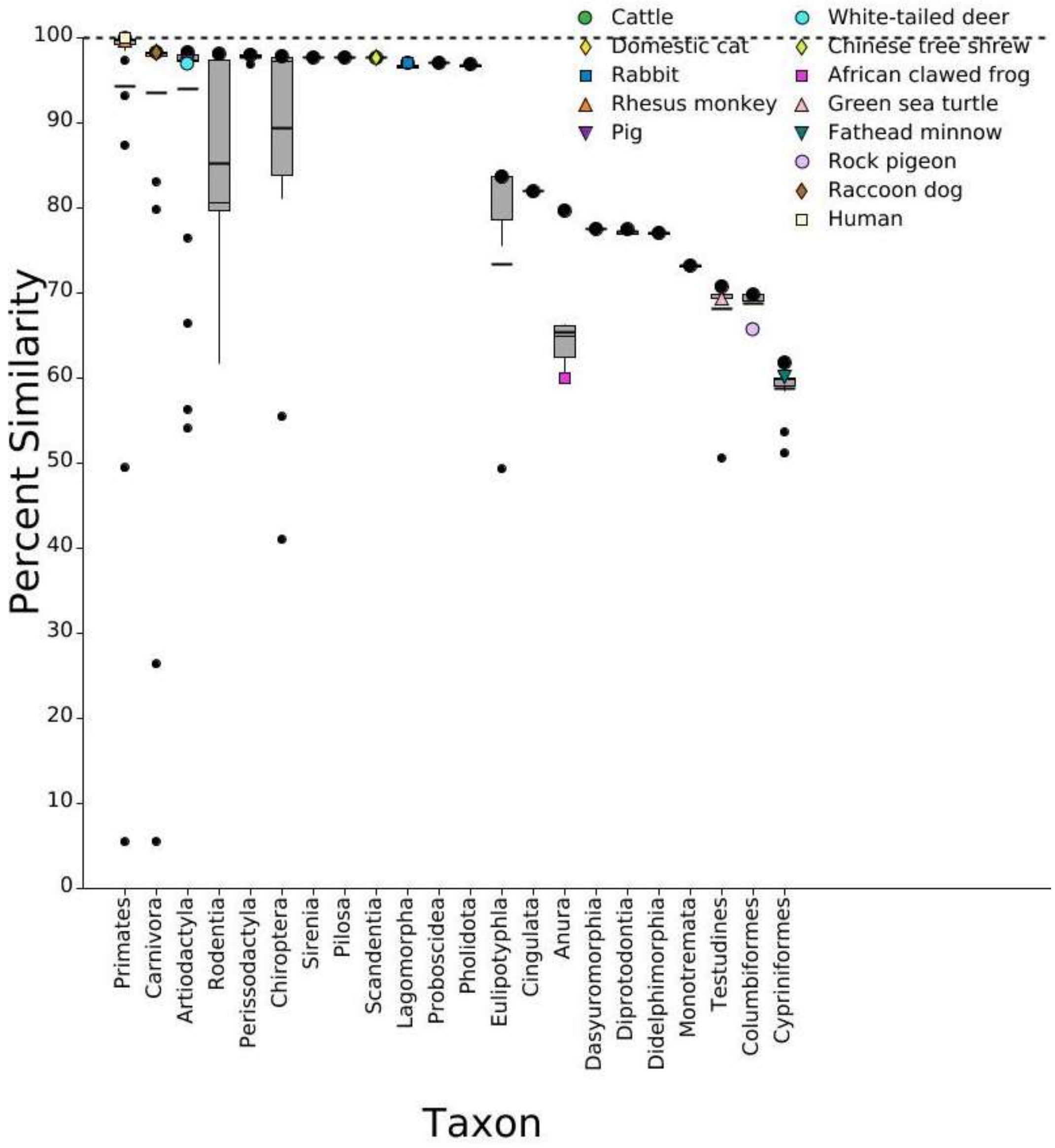

Figure S7b. ras GTPase-activating protein-binding protein 1 (G3BP1)
Query Accession: NP_005745.1
Level 2: cd00780 NTF2 (Nuclear transport factor domain 2)

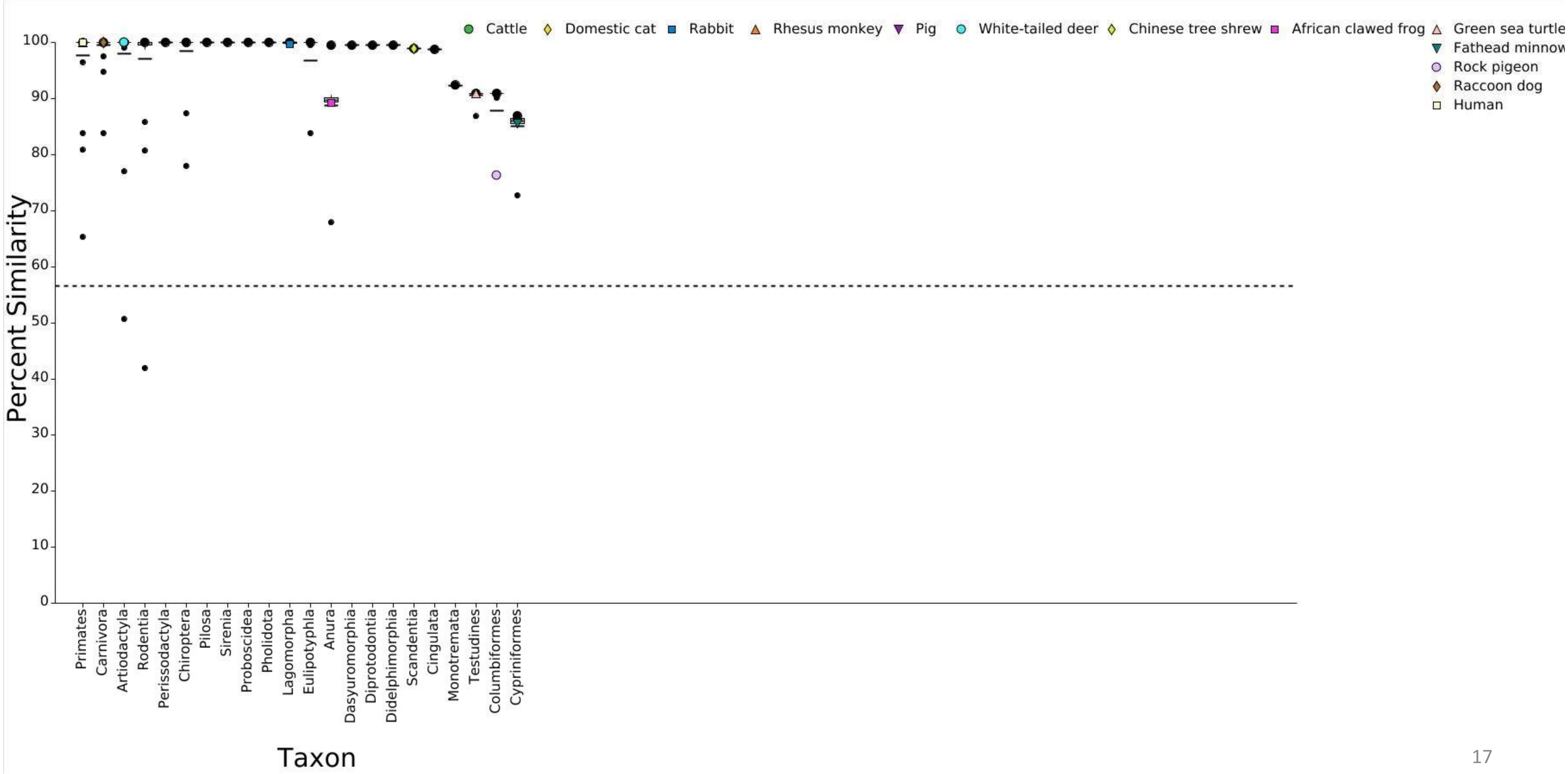

Figure S7c.
ras GTPase-activating protein-binding protein 1 (G3BP1)
Query Accession: NP_005745.1
Level 3: Bond

Total Match
Partial Match
Not a Match

Common Name	Amino Acid 1	Amino Acid 2	Amino Acid 3	Amino Acid 4	Amino Acid 5	Amino Acid 6	Amino Acid 7	Amino Acid 8
Human	15F	18Q	32R	34Y	117E	122N	123K	124F
Western lowland gorilla	15F	18Q	32R	34Y	117E	122N	123K	124F
White-tufted-ear marmoset	125F	128Q	142R	144Y	227E	232N	233K	234F
Green monkey	15F	18Q	32R	34Y	117E	122N	123K	124F
Rhesus monkey	15F	18Q	32R	34Y	117E	122N	123K	124F
Olive baboon	15F	18Q	32R	34Y	117E	122N	123K	124F
Fishing cat	15F	18Q	32R	34Y	117E	122N	123K	124F
Domestic cat	15F	18Q	32R	34Y	117E	122N	123K	124F
Tiger	15F	18Q	32R	34Y	117E	122N	123K	124F
Raccoon dog	15F	18Q	32R	34Y	117E	122N	123K	124F
Canada lynx	15F	18Q	32R	34Y	117E	122N	123K	124F
Bobcat	15F	18Q	32R	34Y	117E	122N	123K	124F
Red fox	15F	18Q	32R	34Y	117E	122N	123K	124F
Dog	15F	18Q	32R	34Y	117E	122N	123K	124F
Eurasian badger	15F	18Q	32R	34Y	117E	122N	123K	124F
Gray squirrel	15F	18Q	32R	34Y	117E	122N	123K	124F
Chinese tree shrew	27F	30Q	44R	46Y	129E	134N	135K	136F
American mink	15F	18Q	32R	34Y	117E	122N	123K	124F
Domestic ferret	15F	18Q	32R	34Y	117E	122N	123K	124F
Big brown bat	28F	31Q	45R	47Y	130E	135N	136K	137F
Cattle	54F	57Q	71R	73Y	156E	161N	162K	163F
Egyptian rousette	53F	56Q	70R	72Y	155E	160N	161K	162F
Pig	15F	18Q	32R	34Y	117E	122N	123K	124F
Rabbit	15F	18Q	32K	34Y	117E	122N	123K	124F
White-tailed deer	54F	57Q	71R	73Y	156E	161N	162K	163F
Malayan pangolin	28F	31Q	45R	47Y	130E	135N	136K	137F
House mouse	55F	58Q	72R	74Y	157E	162N	163K	164F
Golden hamster	15F	18Q	32R	34Y	117E	122N	123K	124F
Norway rat	15F	18Q	32R	34Y	117E	122N	123K	124F
Chinese alligator	15F	18Q	32R	34Y	117E	122N	123K	124F
Rock pigeon	15X	--	21R	23Y	106E	111N	112K	113F
Fathead minnow	15F	18Q	32R	34Y	117E	122N	123K	124F
African clawed frog	15F	18Q	32R	34Y	117E	122N	123K	124F

Figure S7c.
ras GTPase-activating protein-binding protein 1 (G3BP1)
Query Accession: NP_005745.1
Level 3: Distance

Total Match
Partial Match
Not a Match

Common Name	Amino Acid 1	Amino Acid 2	Amino Acid 3	Amino Acid 4	Amino Acid 5	Amino Acid 6	Amino Acid 7	Amino Acid 8
Human	6P	10L	11V	14E	33F	58Q	121A	125Y
Western lowland gorilla	6P	10L	11V	14E	33F	58Q	121A	125Y
White-tufted-ear marmoset	116P	120L	121V	124E	143F	168Q	231A	235Y
Green monkey	6P	10L	11V	14E	33F	58Q	121A	125Y
Rhesus monkey	6P	10L	11V	14E	33F	58Q	121A	125Y
Olive baboon	6P	10L	11V	14E	33F	58Q	121A	125Y
Fishing cat	6P	10L	11V	14E	33F	58Q	121A	125Y
Domestic cat	6P	10L	11V	14E	33F	58Q	121A	125Y
Tiger	6P	10L	11V	14E	33F	58Q	121A	125Y
Raccoon dog	6P	10L	11V	14E	33F	58Q	121A	125Y
Canada lynx	6P	10L	11V	14E	33F	58Q	121A	125Y
Bobcat	6P	10L	11V	14E	33F	58Q	121A	125Y
Red fox	6P	10L	11V	14E	33F	58Q	121A	125Y
Dog	6P	10L	11V	14E	33F	58Q	121A	125Y
Eurasian badger	6P	10L	11V	14E	33F	58Q	121A	125Y
Gray squirrel	6P	10L	11V	14E	33F	58Q	121A	125Y
Chinese tree shrew	18P	22L	23V	26E	45F	70Q	133A	137Y
American mink	6P	10L	11V	14E	33F	58Q	121A	125Y
Domestic ferret	6P	10L	11V	14E	33F	58Q	121A	125Y
Big brown bat	19P	23L	24V	27E	46F	71Q	134A	138Y
Cattle	45P	49L	50V	53E	72F	97Q	160A	164Y
Egyptian rousette	44P	48L	49V	52E	71F	96Q	159A	163Y
Pig	6P	10L	11V	14E	33F	58Q	121A	125Y
Rabbit	6P	10L	11V	14E	33F	58Q	121A	125Y
White-tailed deer	45P	49L	50V	53E	72F	97Q	160A	164Y
Malayan pangolin	19P	23L	24V	27E	46F	71Q	134A	138Y
House mouse	46P	50L	51V	54E	73F	98Q	161A	165Y
Golden hamster	6P	10L	11V	14E	33F	58Q	121A	125Y
Norway rat	6P	10L	11V	14E	33F	58Q	121A	125Y
Chinese alligator	6P	10L	11V	14E	33F	58Q	121A	125Y
Rock pigeon	6P	10L	11V	14E	22F	47Q	110A	114Y
Fathead minnow	6P	10L	11V	14E	33F	58Q	121A	125Y
African clawed frog	6P	10L	11V	14E	33F	58Q	121A	125Y

Figure S8a.
GRB10-interacting GYF protein 2 (GIGYF2)
Query accession: NP_056390.2.
Level 1

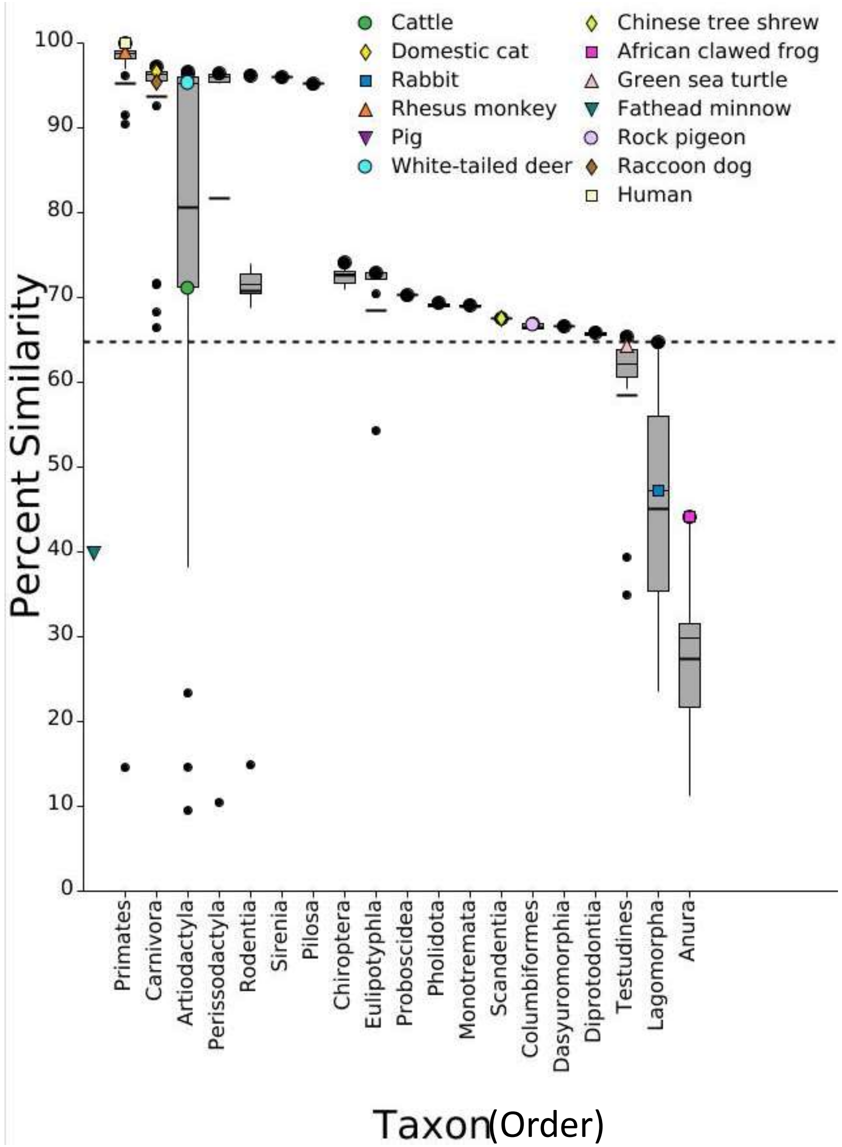

Total Match
Partial Match
Not a Match

Figure S8b. GRB10-interacting GYF protein 2 (GIGYF2) Template accession: NP_056390.2. Variable residues in predicted binding region (Xu et al. 2022) 860-919 (864, 877, 881-896)

Common Name	Amino Acid 1	Amino Acid 2	Amino Acid 3	Amino Acid 4	Amino Acid 5	Amino Acid 6	Amino Acid 7	Amino Acid 8	Amino Acid 9	Amino Acid 10	Amino Acid 11	Amino Acid 12	Amino Acid 13	Amino Acid 14	Amino Acid 15	Amino Acid 16	Amino Acid 17	Amino Acid 18
Human	864N	877H	881E	882R	883K	884R	885K	886E	887L	888E	889V	890Q	891R	892Q	893K	894E	895L	896M
Western lowland gorilla	864N	877H	881E	882R	883K	884R	885K	886E	887L	888E	889V	890Q	891R	892Q	893K	894E	895L	896M
Rhesus monkey	864N	877H	881E	882R	883K	884R	885K	886E	887L	888E	889V	890Q	891R	892Q	893K	894E	895L	896M
Green monkey	864N	877H	881E	882R	883K	884R	885K	886E	887L	888E	889V	890Q	891R	892Q	893K	894E	895L	896M
Olive baboon	885N	898Q	902E	903R	904K	905R	906K	907E	908L	909E	910V	911Q	912R	913Q	914K	915E	916L	917M
Dog	864N	877H	881E	882R	883K	884R	885K	886E	887L	888E	889L	890Q	891R	892Q	893K	894E	895L	896M
Lion	864N	877H	881E	882R	883K	884R	885K	886E	887L	888E	889L	890Q	891R	892Q	893K	894E	895L	896M
Canada lynx	864N	877H	881E	882R	883K	884R	885K	886E	887L	888E	889L	890Q	891R	892Q	893K	894E	895L	896M
Domestic cat	864N	877H	881E	882R	883K	884R	885K	886E	887L	888E	889L	890Q	891R	892Q	893K	894E	895L	896M
Fishing cat	864N	877H	881E	882R	883K	884R	885K	886E	887L	888E	889L	890Q	891R	892Q	893K	894E	895L	896M
Domestic ferret	864N	877H	881E	882R	883K	884R	885K	886E	887L	888E	889L	890Q	891R	892Q	893K	894E	895L	896L
Tiger	864N	877H	881E	882R	883K	884R	885K	886E	887L	888E	889L	890Q	891R	892Q	893K	894E	895L	896M
Red fox	863N	876H	880E	881R	882K	883R	884K	885E	886L	887E	888L	889Q	890R	891Q	892K	893E	894L	895M
American mink	864N	877H	881E	882R	883K	884R	885K	886E	887L	888E	889L	890Q	891R	892Q	893K	894E	895L	896L
Eurasian badger	864N	877H	881E	882R	883K	884R	885K	886E	887L	888E	889L	890Q	891R	892Q	893K	894E	895L	896L
Pig	864N	877H	881E	882R	883K	884R	885K	886E	887L	888E	889L	890Q	891R	892Q	893K	894E	895L	896M
Raccoon dog	867N	880H	884E	885R	886K	887R	888K	889E	890L	891E	892L	893Q	894R	895Q	896K	897E	898L	899M
White-tailed deer	863S	876H	880E	881R	882K	883R	884K	885E	886L	887E	888L	889Q	890R	891Q	892K	893E	894L	895L
Puma	1158N	1171H	1175E	1176R	1177K	1178R	1179K	1180E	1181L	1182E	1183L	1184Q	1185R	1186Q	1187K	1188E	1189L	1190M
Big brown bat	863N	876Q	880E	881R	882K	883R	884K	885E	886M	887E	888L	889Q	890R	891H	892K	893E	894L	895M
Gray squirrel	903N	916H	920E	921R	922K	923R	924K	925E	926L	927E	928L	929Q	930R	931Q	932K	933E	934L	935M
Egyptian rousette	864T	877H	881E	882R	883K	884R	885K	886E	887L	888E	889L	890Q	891R	892Q	893K	894E	895L	896M
Little brown bat	863N	876Q	880E	881R	882K	883R	884K	885E	886L	887E	888L	889Q	890R	891H	892K	893E	894L	895M
House mouse	865N	878H	882E	883R	884K	885R	886K	887E	888L	889E	890L	891Q	892R	893Q	894K	895D	896L	897M
Golden hamster	865N	878H	882D	883R	884K	885R	886K	887E	888L	889E	890L	891Q	892R	893Q	894K	895E	896L	897I
Norway rat	865N	878H	882E	883R	884K	885R	886K	887E	888L	889E	890L	891Q	892R	893Q	894K	895D	896L	897M
Cattle	863N	876H	880E	881R	882K	883R	884K	885E	886L	887E	888L	889Q	890R	891Q	892K	893E	894L	895L
Prairie deer mouse	867N	880H	884E	885R	886K	887R	888K	889E	890L	891E	892L	893Q	894R	895Q	896K	897E	898L	899M
Malayan pangolin	864N	877L	881E	882R	883K	884R	885K	886E	887L	888D	889L	890Q	891R	892Q	893K	894E	895L	896L
Rock pigeon	855S	868L	872E	873R	874K	875R	876K	877E	878L	879E	880L	881Q	882R	883Q	884K	885E	886I	887M
Chinese alligator	858N	871L	875E	876R	877K	878R	879K	880E	881L	882E	883L	884Q	885R	886Q	887K	888E	889I	890M
African clawed frog	899E	904A	908E	909R	910K	911K	912H	913E	--	914E	915A	916Q	917R	918Q	919K	920E	921L	922Q
Fathead minnow	890E	903Q	907E	908Q	909K	910R	911R	912E	913Q	914E	915A	916L	917R	918Q	919Q	920E	921L	922Q

Figure S9a.
Karyopherin subunit alpha2 KPNA2 (Importin)
Query Accession: NP_001307540.1
Level 1

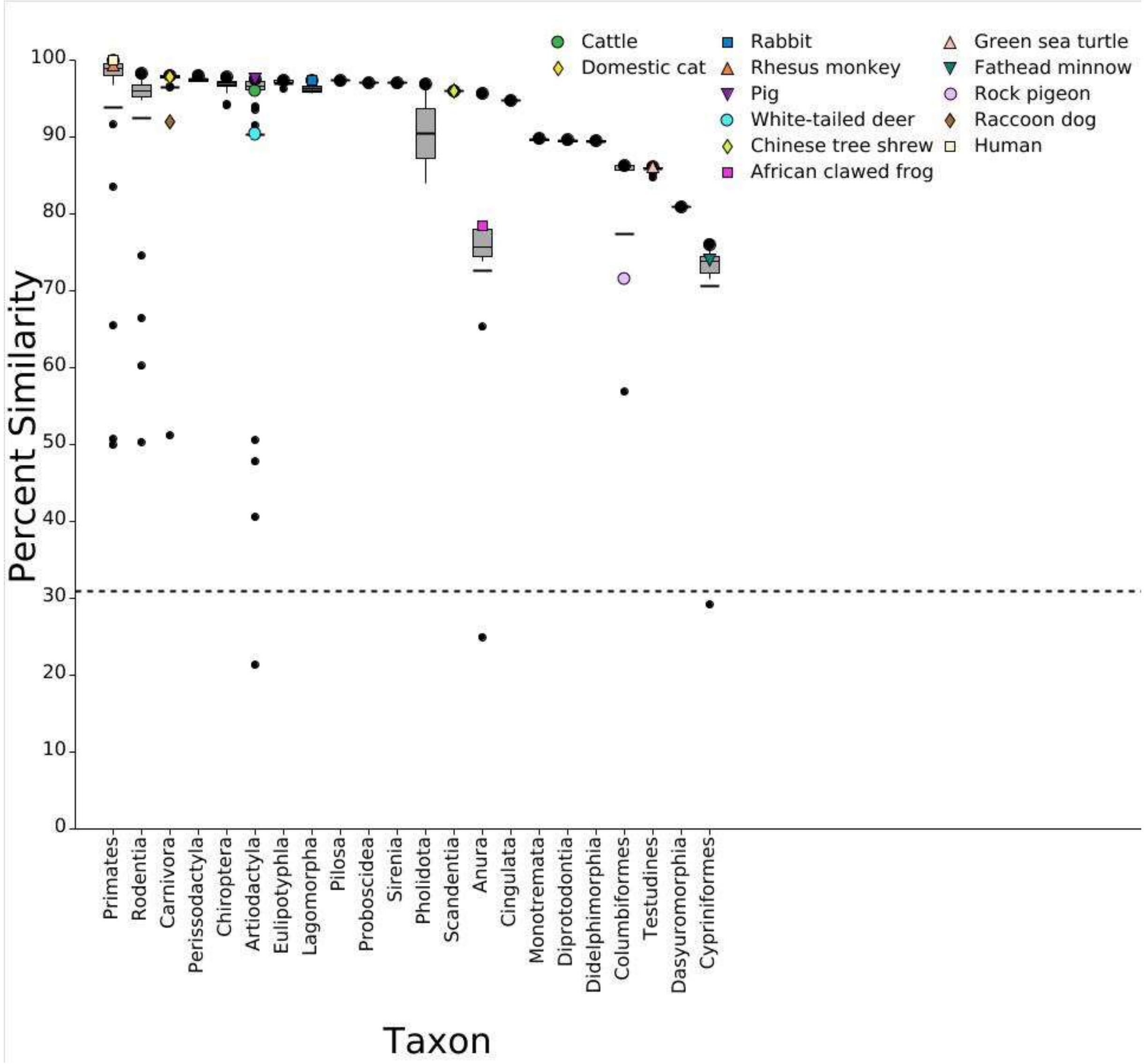

Figure S9b. Karyopherin subunit alpha2 (KPNA2) (Importin subunit alpha-1)
Query Accession: NP_001307540.1
Level 2: pfam01749 IBB, Importin beta binding domain

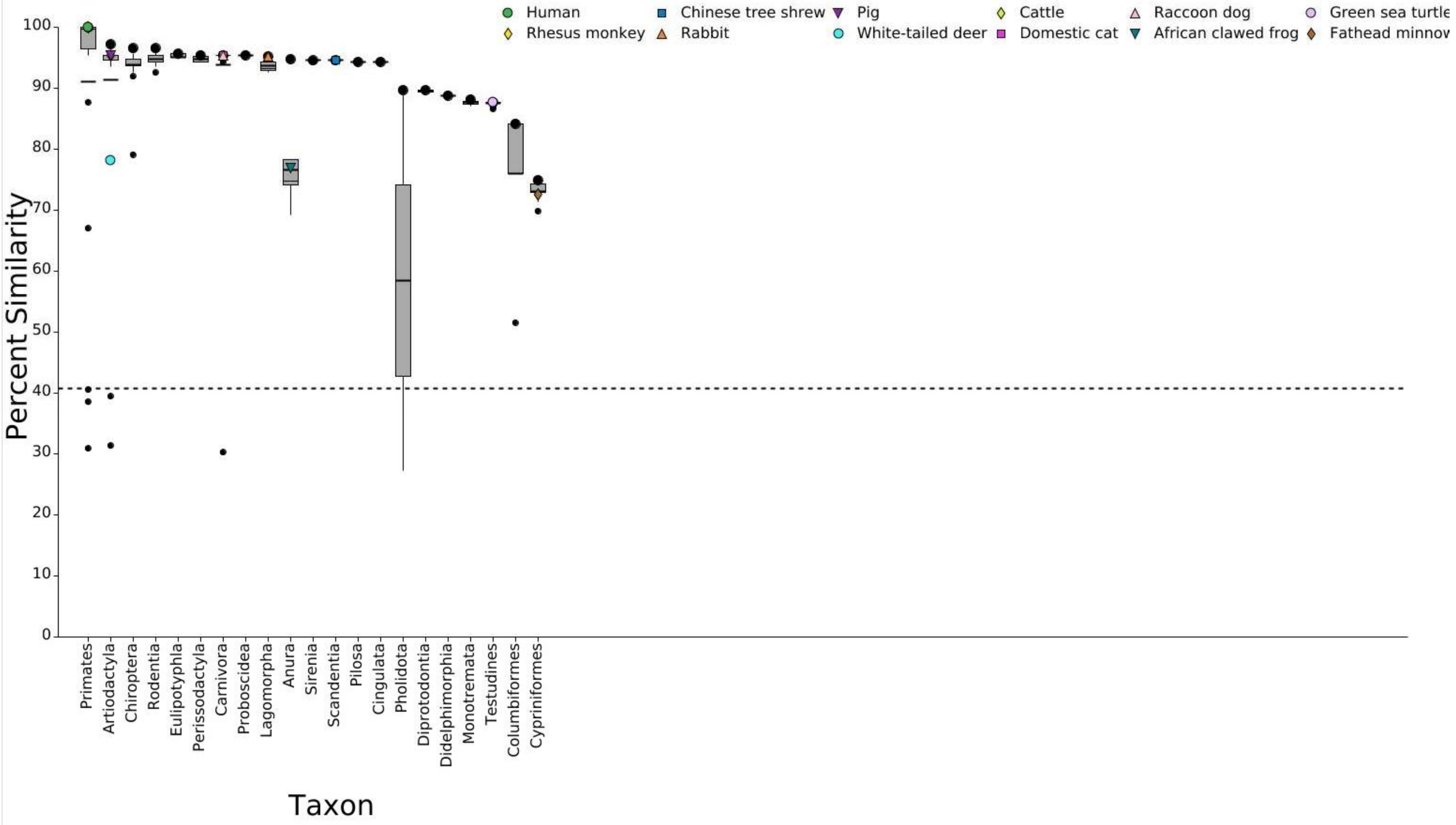

Figure S10a.
Mitochondrial antiviral-signaling protein isoform 1 (MAVS)
Query Accession: NP_065797.2
Level 1

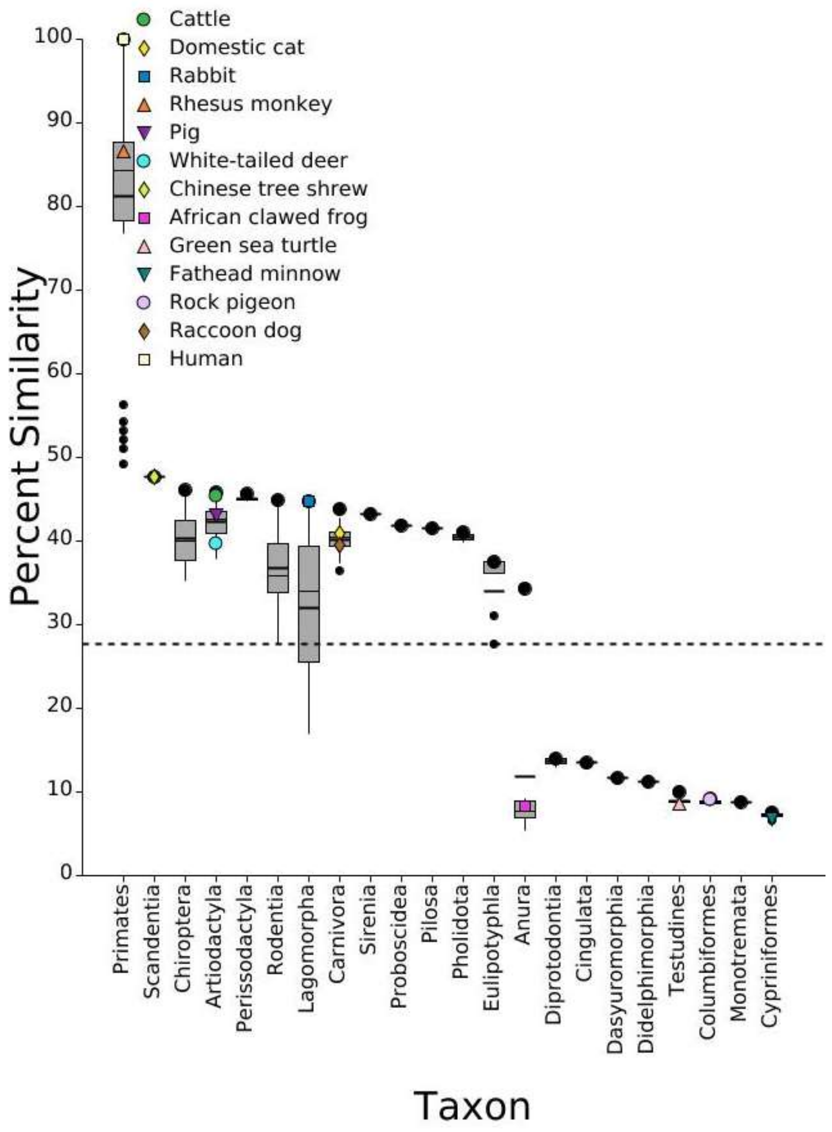

Figure S10b. Mitochondrial antiviral-signaling protein isoform 1 (MAVS)
Query Protein: NP_065797.2
Level 2: cd08811 CARD_IPS1 (Caspase activation and recruitment domain (CARD) found in IPS-1)

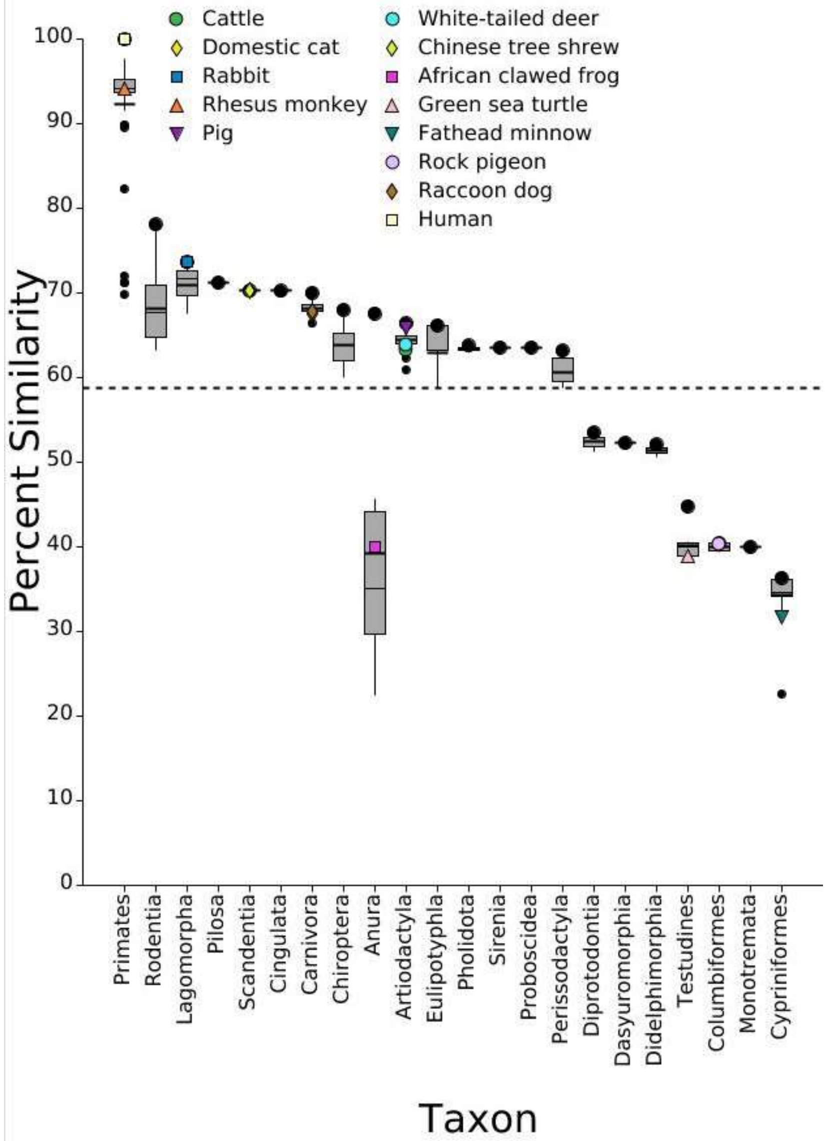

Figure S10c. Mitochondrial antiviral-signaling protein isoform 1 (MAVS) Query Protein: NP 065797.2
Level 3: Bond (Left) and Distance (Right)

Total Match
Partial Match
Not a Match

Common Name	Amino Acid 1	Amino Acid 2	Amino Acid 3	Amino Acid 4	Amino Acid 5	Amino Acid 6	Amino Acid 7	Amino Acid 8
Human	436F	440A	441I	442S	456E	457E	458N	459E
Western lowland gorilla	436F	440A	441I	442S	456E	457E	458N	459E
Rhesus monkey	437S	441A	442I	443S	457E	458E	459N	460E
Olive baboon	437S	441A	442I	443S	457E	458E	459N	460E
Green monkey	436S	440A	441I	442S	456E	457E	458N	459E
White-tufted-ear marmoset	428S	432A	433I	434S	448E	449E	450N	451E
Chinese tree shrew	405S	409A	410I	411S	425E	426E	427N	428E
Northern tree shrew	403S	407A	408I	409S	423E	424E	425N	426E
Cattle	427S	431A	432I	433S	447E	448E	449N	450E
Gray squirrel	416S	420A	421I	422S	434E	435E	436N	437E
American mink	430S	434A	435L	436S	450E	451E	452N	453E
Pig	426S	430A	431I	432S	446E	447E	448N	449E
Tiger	431S	435A	436L	437S	451E	452E	453N	454E
Domestic ferret	430S	434A	435L	436S	450E	451E	452N	453E
Lion	431S	435A	436L	437S	451E	452E	453N	454E
Canada lynx	431S	435A	436L	437S	451E	452E	453N	454E
Bobcat	431S	435A	436L	437S	451E	452E	453N	454E
Domestic cat	431S	435A	436L	437S	451E	452E	453N	454E
Fishing cat	431S	435A	436L	437S	451E	452E	453N	454E
Puma	431S	435A	436L	437S	451E	452E	453N	454E
Dog	426S	430A	431L	432S	446E	447E	448N	449E
Eurasian badger	430S	434A	435L	436S	450E	451E	452N	453E
Malayan pangolin	460S	464A	465I	466S	479E	480E	481N	482E
White-tailed deer	414S	418A	419I	420S	434E	435E	436N	437E
Raccoon dog	446S	450A	451L	452S	466E	467E	468N	469E
Red fox	426S	430A	431L	432S	446E	447E	448N	449E
Big brown bat	425S	429A	430I	431S	443E	444E	445N	446E
Egyptian rousette	427S	431A	432I	433S	447E	448E	449N	450E
Golden hamster	406S	410A	411I	412S	426E	427E	428S	429E
House mouse	414S	418A	419I	420S	434E	435E	436N	437E
Norway rat	416S	420A	421I	422S	436E	437E	438N	439E
Prairie deer mouse	416S	420A	421I	422S	436E	437E	438N	439E
Bank vole	415S	419A	420I	421S	435E	436E	437N	438E
Rock pigeon	--	--	--	--	--	589E	590N	--
Chinese alligator	588S	592L	593L	594S	618E	619E	620S	658S
African clawed frog	430S	434E	435I	436S	493E	494E	495N	537E
Fathead minnow	409S	413E	414I	415S	432E	433Q	434T	437P

Total Match
Partial Match
Not a Match

Common Name	Amino Acid 1	Amino Acid 2	Amino Acid 3	Amino Acid 4
Human	438D	439L	453H	455P
Western lowland gorilla	438D	439L	453H	455P
Rhesus monkey	439D	440L	454R	456P
Olive baboon	439D	440L	454H	456P
Green monkey	438D	439L	453H	455P
White-tufted-ear marmoset	430D	431L	445H	447P
Chinese tree shrew	407D	408L	422L	424P
Northern tree shrew	405D	406L	420L	422P
Cattle	429D	430L	444N	446P
Gray squirrel	418D	419L	431Q	433P
American mink	432D	433L	447N	449P
Pig	428D	429L	443N	445P
Tiger	433D	434L	448N	450P
Domestic ferret	432D	433L	447N	449P
Lion	433D	434L	448N	450P
Canada lynx	433D	434L	448N	450P
Bobcat	433D	434L	448N	450P
Domestic cat	433D	434L	448N	450P
Fishing cat	433D	434L	448N	450P
Puma	433D	434L	448N	450P
Dog	428D	429L	443N	445P
Eurasian badger	432D	433L	447N	449P
Malayan pangolin	462D	463L	476N	--
White-tailed deer	416D	417L	431N	433P
Raccoon dog	448D	449L	463N	465P
Red fox	428D	429L	443N	445P
Big brown bat	427D	428L	440N	442P
Egyptian rousette	429D	430L	444N	446P
Golden hamster	408D	409L	423R	425P
House mouse	416D	417L	431H	433P
Norway rat	418D	419L	433H	435P
Prairie deer mouse	418D	419L	433H	435P
Bank vole	417D	418L	432R	434P
Rock pigeon	--	--	585L	--
Chinese alligator	590R	591F	611G	617P
African clawed frog	432N	433L	461H	492P
Fathead minnow	411D	412L	426R	--

Figure S11a.
Melanoma-differentiation-associated gene 5 (MDA5) Query Accession: NP_071451.2
Level 1

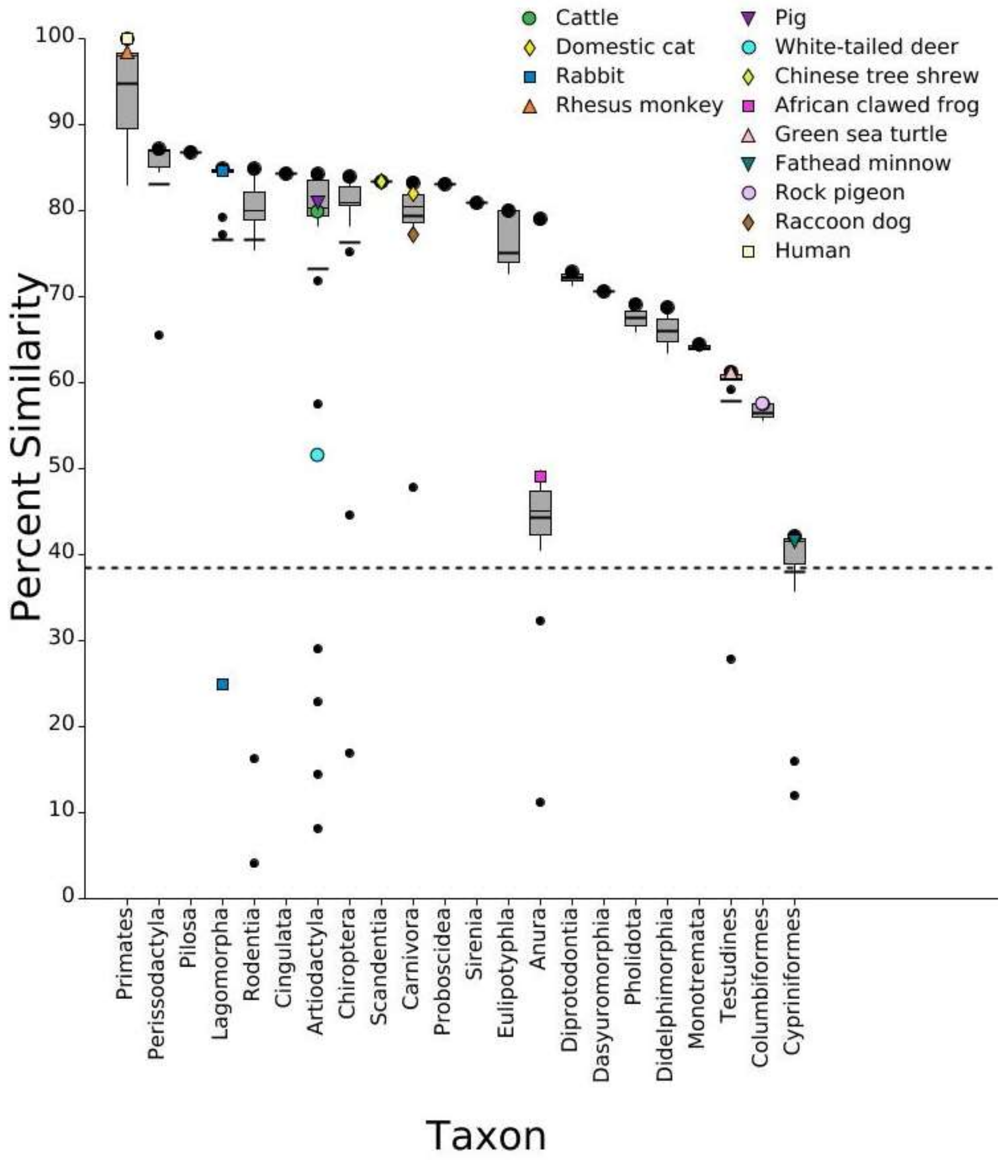

Figure S11b. Melanoma-differentiation-associated gene 5 (MDA5)
Level 2: cd08818 (CARD_MDA5_r1) (residues 8-99)

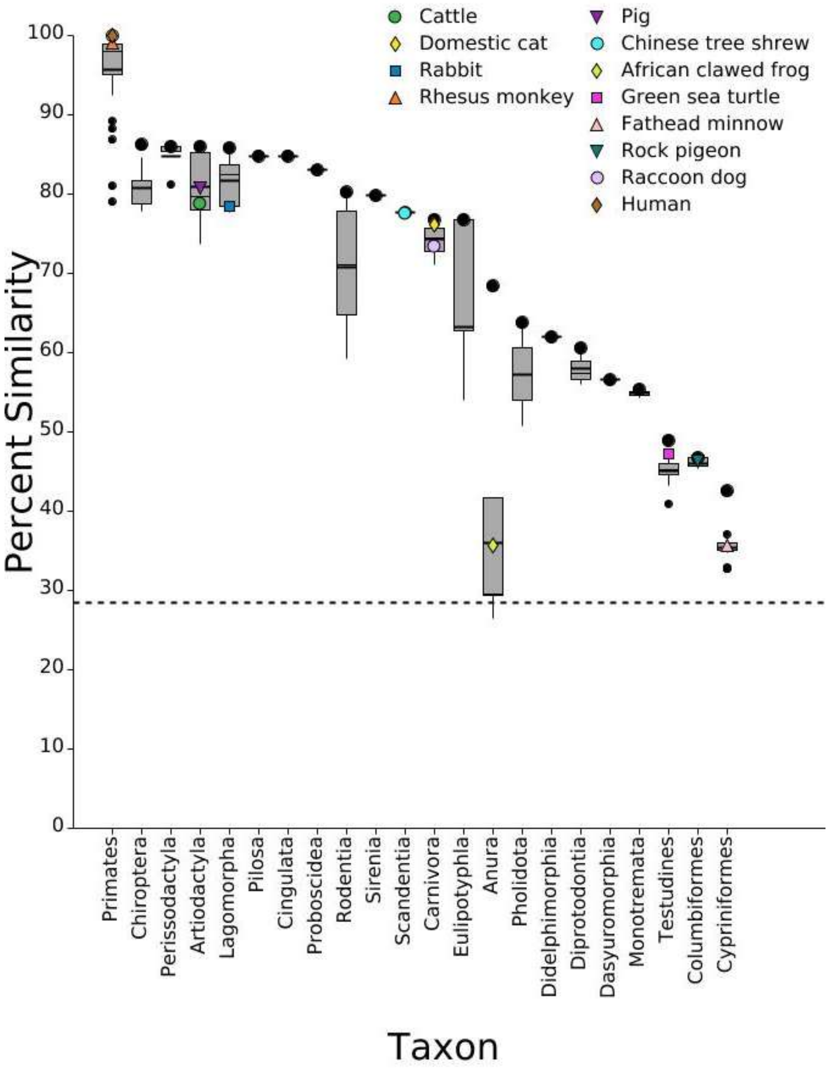

MDA5 Query Accession: NP_071451.2
Level 2 cd08819 CARD_MDA5_r2

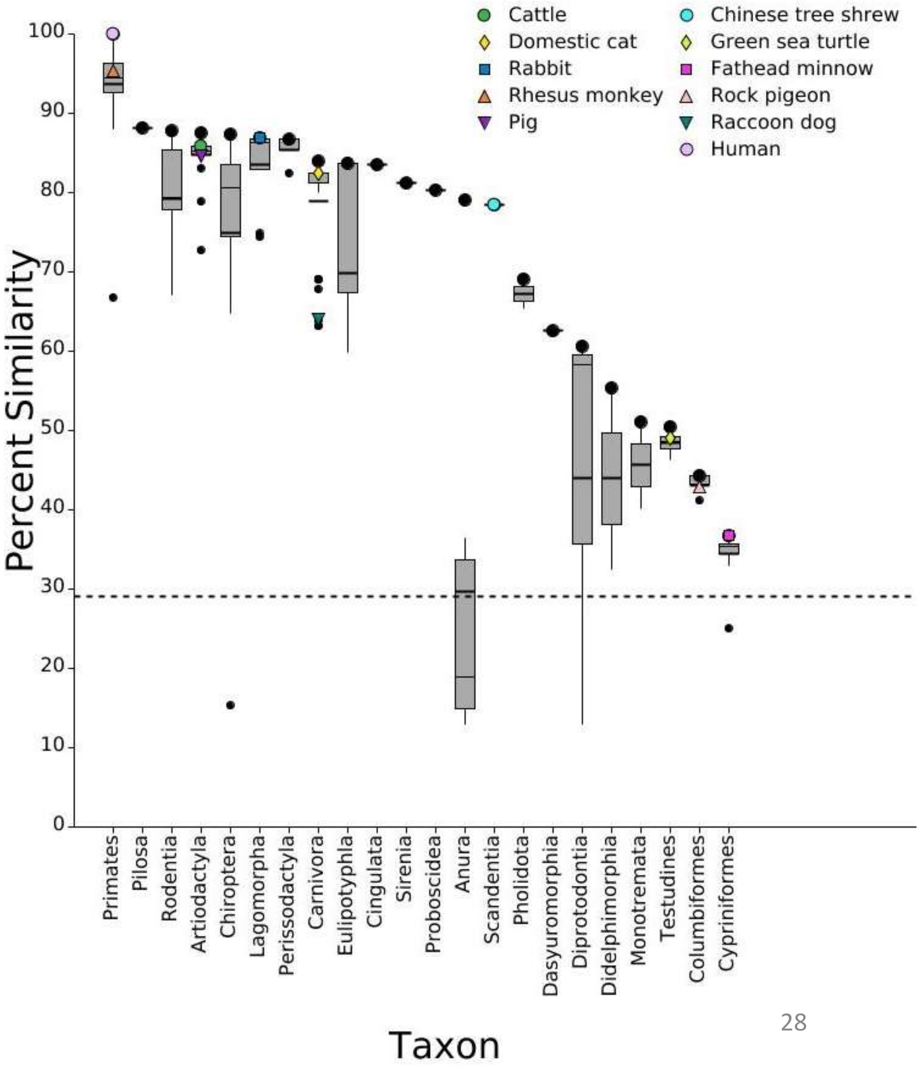

Figure S11b. Melanoma-differentiation-associated gene 5 (MDA5) Query Accession: NP_071451.2 Level 2 cd15807 MDA5_C (C-terminal domain)

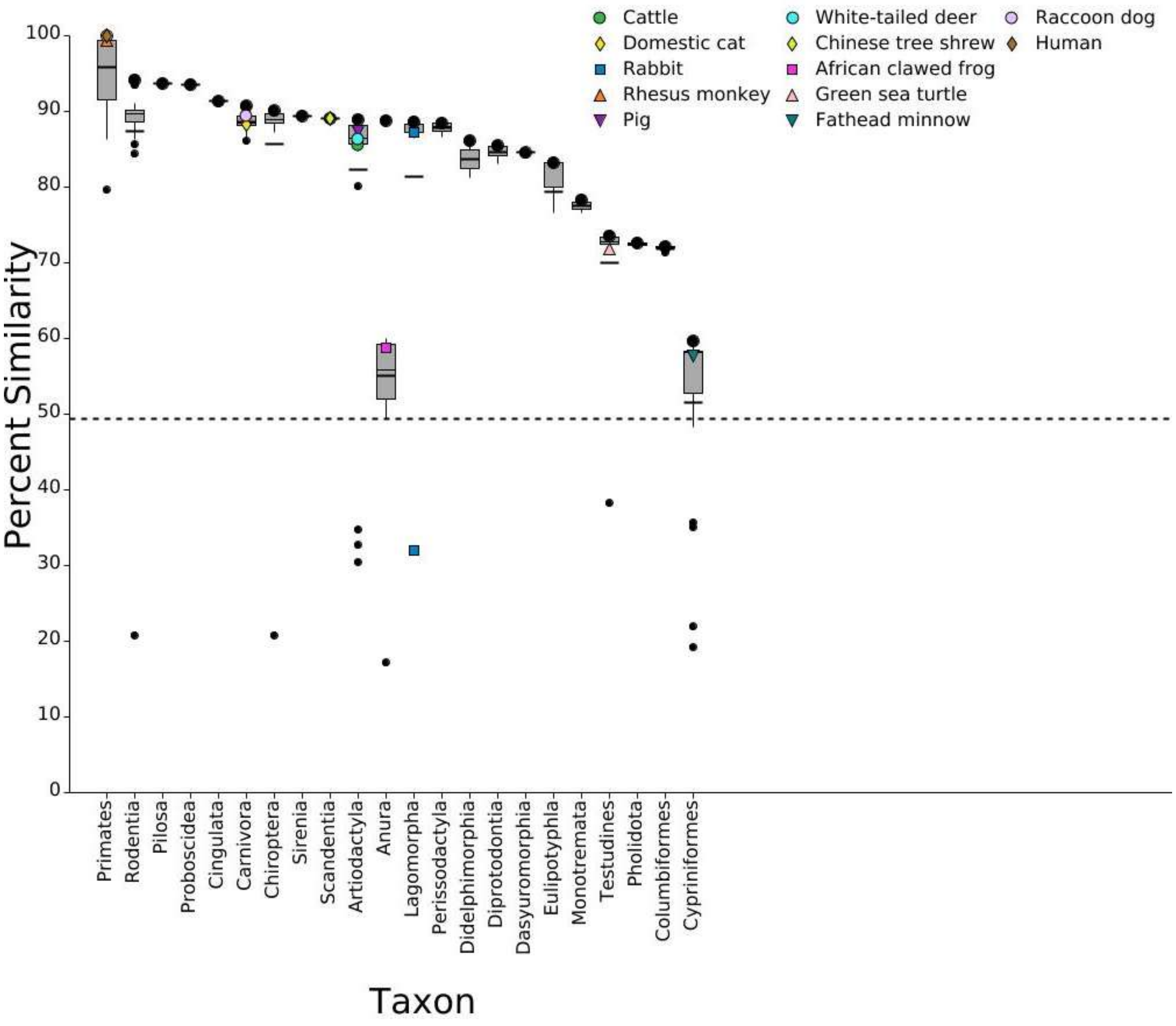

MDA5 Query Accession: NP_071451.2 Level 2 cd12090 MDA5_ID (insert domain)

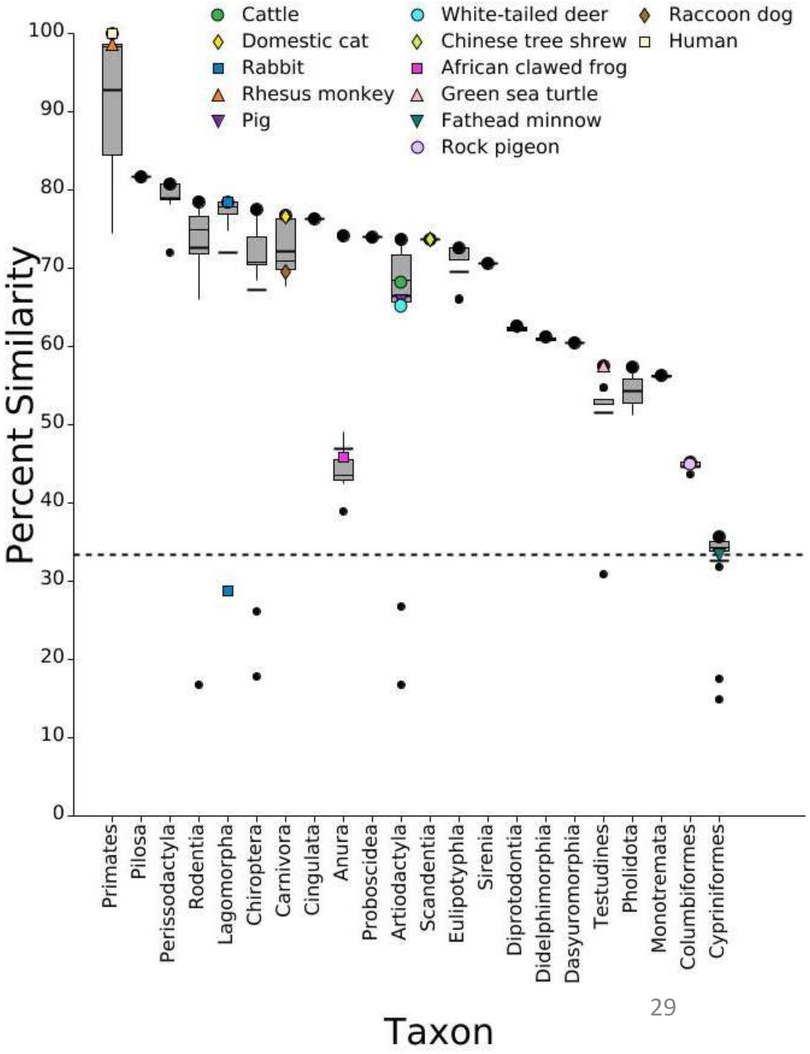

Figure S11c. Melanoma-differentiation-associated gene 5 (MDA5) Query Accession: NP_071451.2 ISGylation,(1-3) CARD oligomerization (4-5), and phosphorylation (6-7) sites

Total Match
Partial Match
Not a Match

Common Name	Amino Acid 1	Amino Acid 2	Amino Acid 3	Amino Acid 4	Amino Acid 5	Amino Acid 6	Amino Acid 7
Human	23K	43K	68K	74G	75W	88S	104S
Western lowland gorilla	23K	43K	68K	74G	75W	88S	104S
Rhesus monkey	23K	43K	68K	74G	75W	88S	104S
Olive baboon	23K	43K	68K	74G	75W	88S	104S
Green monkey	23K	43K	68K	74G	75W	88S	104S
White-tufted-ear marmoset	23K	43K	68K	74G	75W	88S	104S
Eastern cottontail	23K	43K	68N	74G	75W	88N	104S
Rabbit	23K	43R	68K	74G	75W	88S	104S
Egyptian rousette	21K	41K	66K	72G	73W	86S	102S
Gray squirrel	23K	43K	68K	74G	75W	88N	104S
Chinese tree shrew	23K	43K	68K	74G	75W	88N	104S
Bobcat	23K	43K	68K	74G	75W	88S	104S
Canada lynx	23K	43K	68K	74G	75W	88S	104S
Lion	23K	43K	68K	74G	75W	88S	104S
Domestic cat	23K	43K	68K	74G	75W	88S	104S
Puma	23K	43K	68R	74G	75W	88S	104S
Fishing cat	23K	43K	68K	74G	75W	88S	104S
Tiger	23K	43K	68K	74G	75W	88S	104S
Big brown bat	23R	43K	68K	74G	75W	88N	104S
Jamaican fruit-eating bat	23K	43K	68K	74G	75W	88N	104S
Pig	24K	44K	69K	75G	76W	89N	105S
Little brown bat	23R	43K	68K	74G	75W	88N	104S
Prairie deer mouse	23K	43K	68K	74G	75W	88S	102S
Cattle	24K	44K	69R	75G	76W	89N	105S
Golden hamster	23K	43K	68K	74G	75W	88S	102S
House mouse	23K	43K	68Q	74G	75W	88N	104S
Norway rat	23K	43K	68Q	74G	75W	88N	104S
Domestic ferret	23K	43K	68K	74G	75W	88S	104S
American mink	23K	43K	68K	74G	75W	88S	104S
Dog	24K	44K	69K	75G	76W	89S	105S
Red fox	24K	44K	69Q	75G	76W	89S	105S
Raccoon dog	24K	44K	69E	75G	76W	89S	105S
Malayan pangolin	29R	44R	69N	75G	76W	89S	102S
Rock pigeon	22E	42R	67R	73G	74W	87C	103S
Chinese alligator	--	--	--	--	--	8C	24S
African clawed frog	23V	43R	68K	74G	75W	88C	101T
Fathead minnow	21K	41K	66Q	72G	73W	86C	--

Figure S12a.
Interferon regulatory factor 3 (IRF3)
Query Accession: NP_001562.1
Level 1

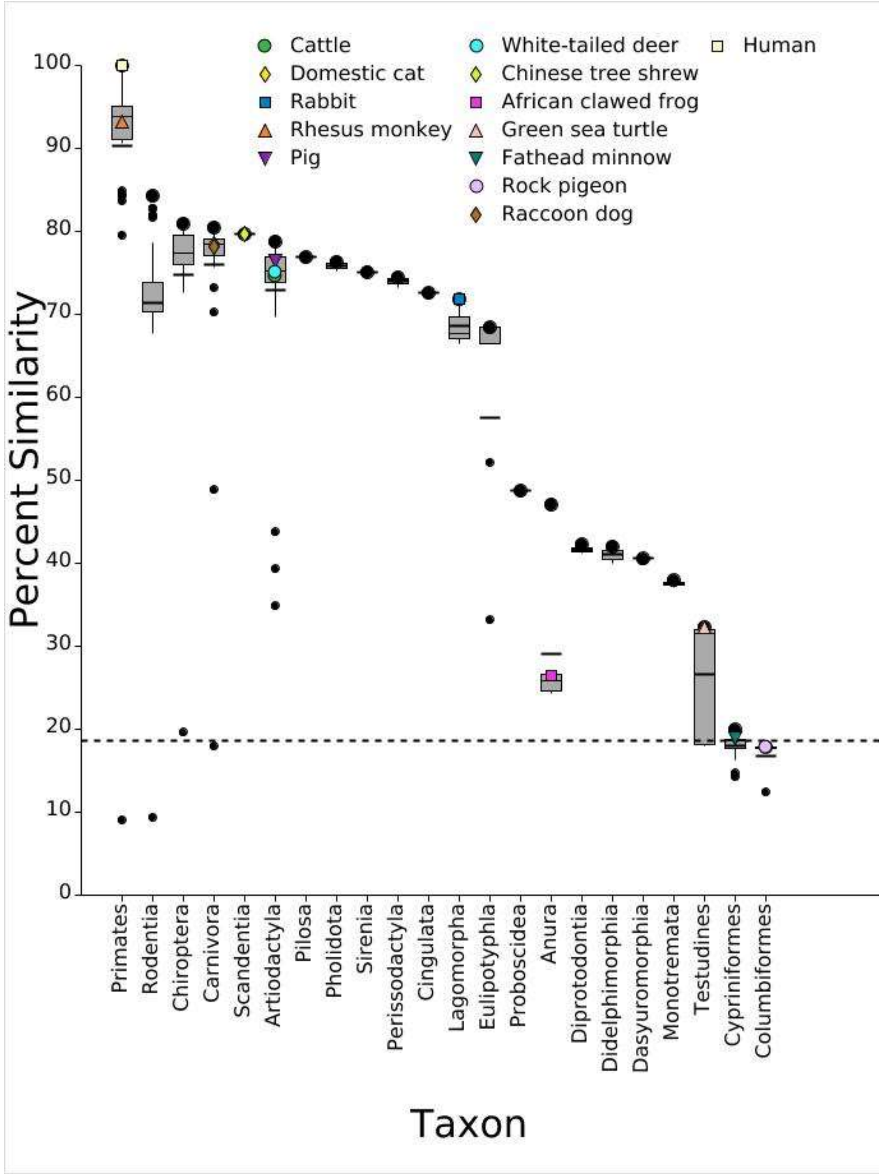

Figure S12b. Interferon regulatory factor 3 (IRF3) Query Accession: NP_001562.1 Level 2: cd00103 (IRF transcription factor DNA sequence recognition site)

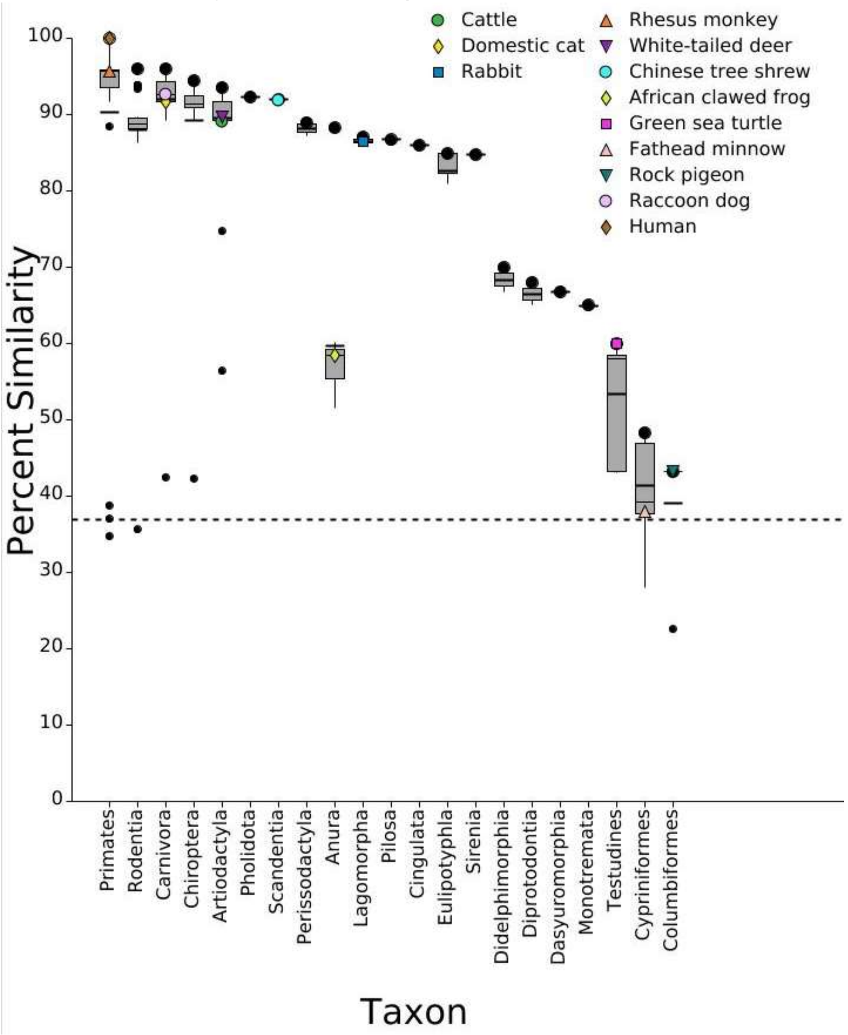

IRF3 Query Accession: NP_001562.1 Level 2: pfam10401 (cl11061: IRF-3 Superfamily res 202-380)

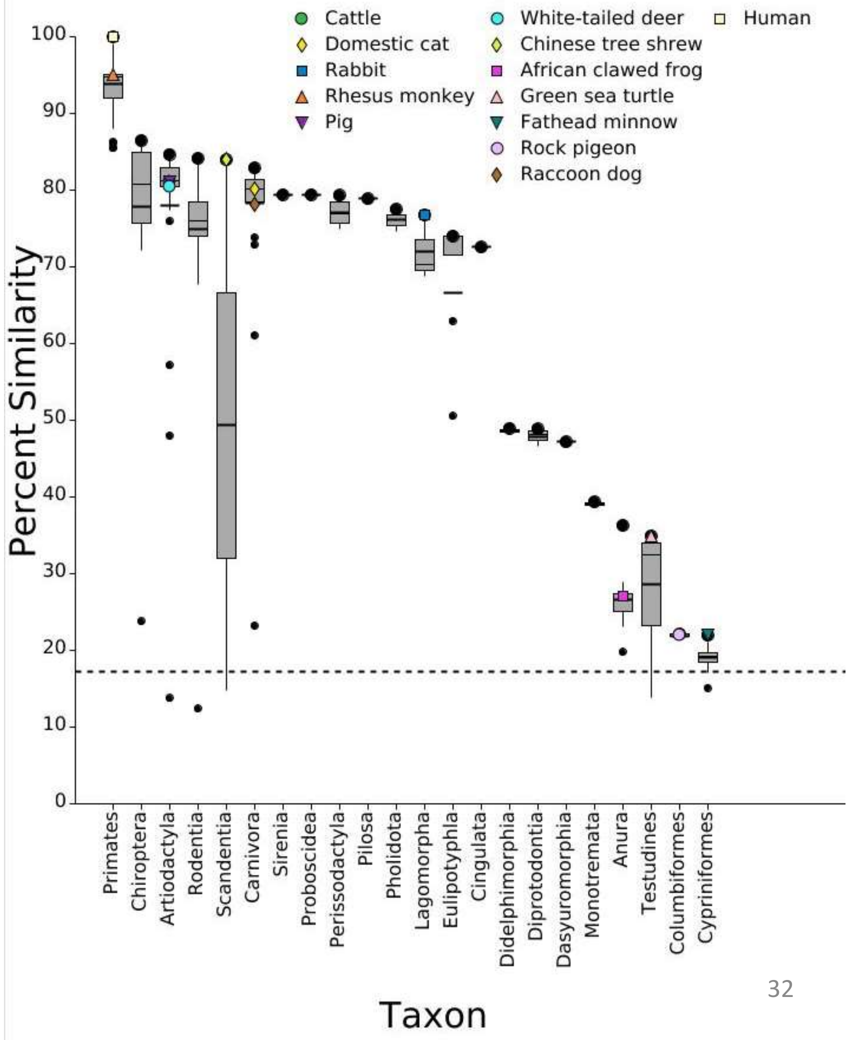

Figure S12c. Interferon regulatory factor 3 (IRF3) Query Accession: NP_001562.1 Level 3: Bond

Total Match
Partial Match
Not a Match

Common Name	Amino Acid 1	Amino Acid 2	Amino Acid 3	Amino Acid 4	Amino Acid 5	Amino Acid 6	Amino Acid 7	Amino Acid 8	Amino Acid 9	Amino Acid 10
Human	211R	263H	285R	288H	290H	313K	349G	350E	351S	360K
Western lowland gorilla	211R	263H	285R	288H	290H	313K	349G	350E	351S	360K
Green monkey	207R	259H	281R	284H	286H	308K	344G	345E	346S	355K
Olive baboon	207R	259H	281R	284H	286H	308K	344G	345E	346S	355K
Rhesus monkey	207R	259H	281R	284H	286H	308K	344G	345E	346S	355K
White-tufted-ear marmoset	211R	263H	285R	288Q	290H	313K	349G	350E	351P	360K
Gray squirrel	210R	262R	284R	287H	289H	312K	348G	349E	350S	359K
Chinese tree shrew	344R	396R	418R	421H	423G	446K	482G	483Q	484S	493K
Big brown bat	211R	263R	285R	288P	290H	313K	349G	350E	351P	360K
Egyptian rousette	211R	263R	285R	288H	290H	313K	349G	350E	351S	360K
Bobcat	226R	277R	299R	302H	304H	327K	363G	364E	365P	374K
Canada lynx	211R	262R	284R	287H	289H	312K	348G	349E	350P	359K
Tiger	211R	262R	284R	287H	289H	312K	348G	349E	350P	359K
Lion	211R	262R	284R	287H	289H	312K	348G	349E	350P	359K
Domestic cat	241R	292R	314R	317H	319H	342K	378G	379E	380P	389K
Raccoon dog	248R	300R	322R	325H	327H	350K	386G	387E	388P	397K
Fishing cat	211R	262R	284R	287H	289H	312K	348G	349E	350P	359K
Dog	211R	263R	285R	288H	290H	313K	349G	350E	351P	360K
Red fox	212R	264R	286R	289H	291H	314K	350G	351E	352P	361K
Domestic ferret	211R	263R	285R	288H	290H	313K	349G	350E	351P	360K
Pig	209R	261R	283R	286H	288H	311K	347G	348Q	349S	358K
American mink	211R	263R	285R	288H	290H	313K	349G	350E	351P	360K
Eurasian badger	211R	263R	285R	288H	290H	313K	349G	350E	351P	360K
Malayan pangolin	263R	315R	337R	340H	342H	365K	401G	402E	403P	412K
White-tailed deer	209R	261R	283R	286H	288H	311K	347G	348Q	349A	358K
Cattle	207R	259R	281R	284H	286H	309K	345G	346Q	347S	356K
Prairie deer mouse	205R	257H	279R	282H	284H	307K	343G	344E	345S	354K
Norway rat	251R	303Q	325R	328H	330H	353K	389G	390E	391S	400K
Golden hamster	236R	288H	310R	313H	315H	338K	374G	375E	376S	385K
House mouse	205R	256Q	278R	281H	283H	306K	342G	343E	344M	353K
Chinese alligator	223R	275R	297R	300R	302H	322K	358G	359E	360K	370R
African clawed frog	261R	312K	334R	337K	339R	358K	395G	396E	397L	406K
Fathead minnow	225R	274R	296R	299K	301K	322Q	358G	359E	360K	369K
Rock pigeon	227R	287R	309R	312Q	314K	333R	370G	371E	372E	381R

Figure S12c. Interferon regulatory factor 3 (IRF3)
Query Accession: NP_001562.1
Level 3: Distance

Total Match
Partial Match
Not a Match

Common Name	Amino Acid 1	Amino Acid 2	Amino Acid 3	Amino Acid 4	Amino Acid 5	Amino Acid 6
Human	260Y	264V	287G	289C	292Y	362L
Chinese rufous horseshoe bat	259Y	263V	286G	288C	291Y	361L
Greater horseshoe bat	314Y	318V	341G	343C	346Y	416L
Big brown bat	260Y	264V	287G	289C	292Y	362L
Common bats	258Y	262V	285G	287C	290Y	360L
Little brown bat	260Y	264V	287G	289C	292Y	362L
Common bats	260Y	264V	287G	289C	292Y	362L
Western lowland gorilla	260Y	264V	287G	289C	292Y	362L
Green monkey	256Y	260V	283G	285C	288Y	357L
Olive baboon	256Y	260V	283G	285C	288Y	357L
Rhesus monkey	256Y	260V	283G	285C	288Y	357L
White-tufted-ear marmoset	260Y	264V	287G	289C	292Y	362L
Gray squirrel	259Y	263V	286G	288C	291Y	361L
Chinese tree shrew	393Y	397V	420G	422C	425Y	495L
Big brown bat	260Y	264V	287G	289C	292Y	362L
Egyptian rousette	260F	264V	287G	289C	292Y	362L
Bobcat	274Y	278V	301G	303C	306Y	376L
Canada lynx	259Y	263V	286G	288C	291Y	361L
Tiger	259Y	263V	286G	288C	291Y	361L
Lion	259Y	263V	286G	288C	291Y	361L
Domestic cat	289Y	293V	316G	318C	321Y	391L
Raccoon dog	297Y	301V	324G	326C	329Y	399L
Fishing cat	259Y	263V	286G	288C	291Y	361L
Dog	260Y	264V	287G	289C	292Y	362L
Red fox	261Y	265V	288G	290C	293Y	363L
Domestic ferret	260Y	264V	287G	289C	292Y	362L
Pig	258Y	262V	285G	287C	290Y	360L
American mink	260Y	264V	287G	289C	292Y	362L
Eurasian badger	260Y	264V	287G	289C	292Y	362F
Malayan pangolin	312Y	316V	339G	341C	344Y	414L
White-tailed deer	258Y	262V	285G	287C	290Y	360L
Cattle	256Y	260V	283G	285C	288Y	358L
Prairie deer mouse	254Y	258V	281G	283S	286F	356L
Norway rat	300Y	304V	327G	329S	332F	402L
Golden hamster	285Y	289V	312G	314S	317F	387L
House mouse	253Y	257V	280G	282S	285F	355L
Chinese alligator	272Y	276L	299G	301C	304F	372L
African clawed frog	309E	313L	336G	338C	341F	408F
Fathead minnow	271F	275I	298D	300C	303F	371L
Rock pigeon	284F	288L	311C	313C	316Y	383L

Figure S12c. Interferon regulatory factor 3 (IRF3)
Query Accession: NP_001562.1
Level 3: Cleavage

Total Match
Partial Match
Not a Match

Common Name	Amino Acid 1	Amino Acid 2	Amino Acid 3	Amino Acid 4	Amino Acid 5	Amino Acid 6	Amino Acid 7
Human	267C	268L	269G	270G	271G	272L	273A
Chinese rufous horseshoe bat	266S	267L	268G	269G	270G	271L	272A
Greater horseshoe bat	321S	322L	323G	324G	325G	326L	327A
Big brown bat	267S	268L	269G	270G	271G	272L	273A
Common bats	265C	266L	267G	268G	269G	270L	271A
Little brown bat	267G	268L	269G	270G	271G	272L	273A
Common bats	267G	268L	269G	270G	271G	272L	273A
Western lowland gorilla	267C	268L	269G	270G	271G	272L	273A
Green monkey	263S	264L	265G	266G	267G	268L	269A
Olive baboon	263S	264L	265G	266G	267G	268L	269A
Rhesus monkey	263S	264L	265G	266G	267G	268L	269A
White-tufted-ear marmoset	267R	268L	269G	270G	271G	272L	273A
Gray squirrel	266G	267L	268R	269G	270G	271L	272A
Chinese tree shrew	400C	401L	402G	403G	404G	405L	406A
Big brown bat	267S	268L	269G	270G	271G	272L	273A
Egyptian rousette	267S	268L	269G	270G	271G	272L	273A
Bobcat	281C	282L	283G	284G	285G	286L	287A
Canada lynx	266C	267L	268G	269G	270G	271L	272A
Tiger	266C	267L	268G	269G	270G	271L	272A
Lion	266C	267L	268G	269G	270G	271L	272A
Domestic cat	296C	297L	298G	299G	300G	301L	302A
Raccoon dog	304C	305L	306G	307G	308G	309L	310A
Fishing cat	266C	267L	268G	269G	270G	271L	272A
Dog	267C	268L	269G	270G	271G	272L	273A
Red fox	268C	269L	270G	271G	272G	273L	274A
Domestic ferret	267C	268L	269G	270R	271G	272L	273A
Pig	265C	266L	267G	268G	269G	270L	271A
American mink	267C	268L	269G	270G	271G	272L	273A
Eurasian badger	267C	268L	269G	270G	271G	272L	273A
Malayan pangolin	319S	320L	321G	322G	323G	324L	325A
White-tailed deer	265C	266L	267G	268G	269G	270L	271A
Cattle	263C	264L	265G	266G	267G	268L	269A
Prairie deer mouse	261G	262L	263G	264K	265G	266L	267A
Norway rat	307G	308L	309G	310K	311G	312L	313V
Golden hamster	292G	293L	294G	295K	296G	297L	298A
House mouse	260G	261L	262G	263N	264G	265L	266A
Chinese alligator	279G	280L	281G	282P	283G	284V	285V
African clawed frog	316N	317L	318E	319E	320G	321V	322L
Fathead minnow	278S	279I	280Q	281R	282G	283L	284Q
Rock pigeon	291V	292M	293D	294R	295G	296L	297I

Figure S13a.
Ubiquitin-like interferon stimulated gene 15 (ISG15)
Query Accession: NP_005092.1
Level 1

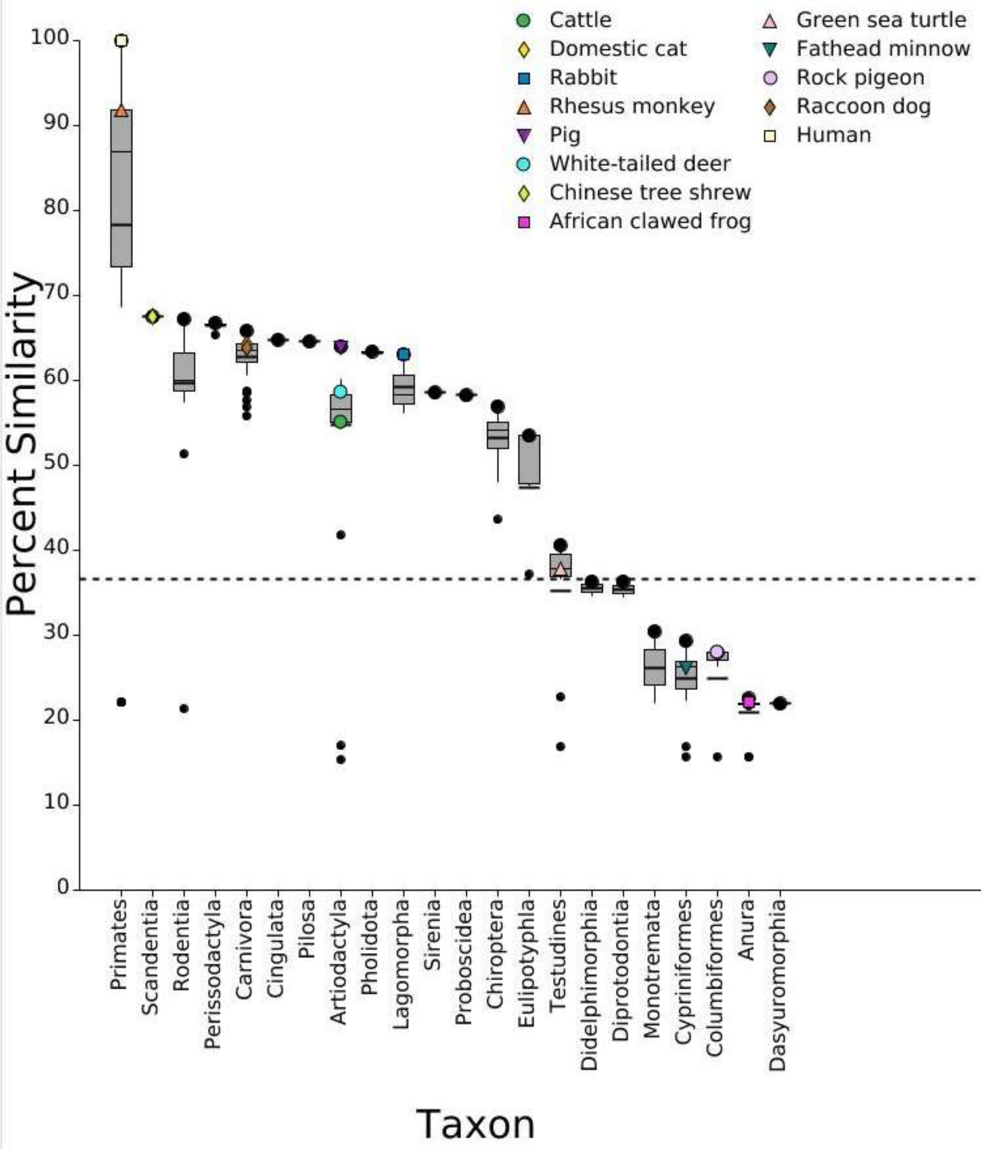

Figure S13b. Ubiquitin-like interferon stimulated gene 15 (ISG15)
Query Accession: NP_005092.1
Level 2: cd01792 ubiquitin-like (Ubl) domain 1

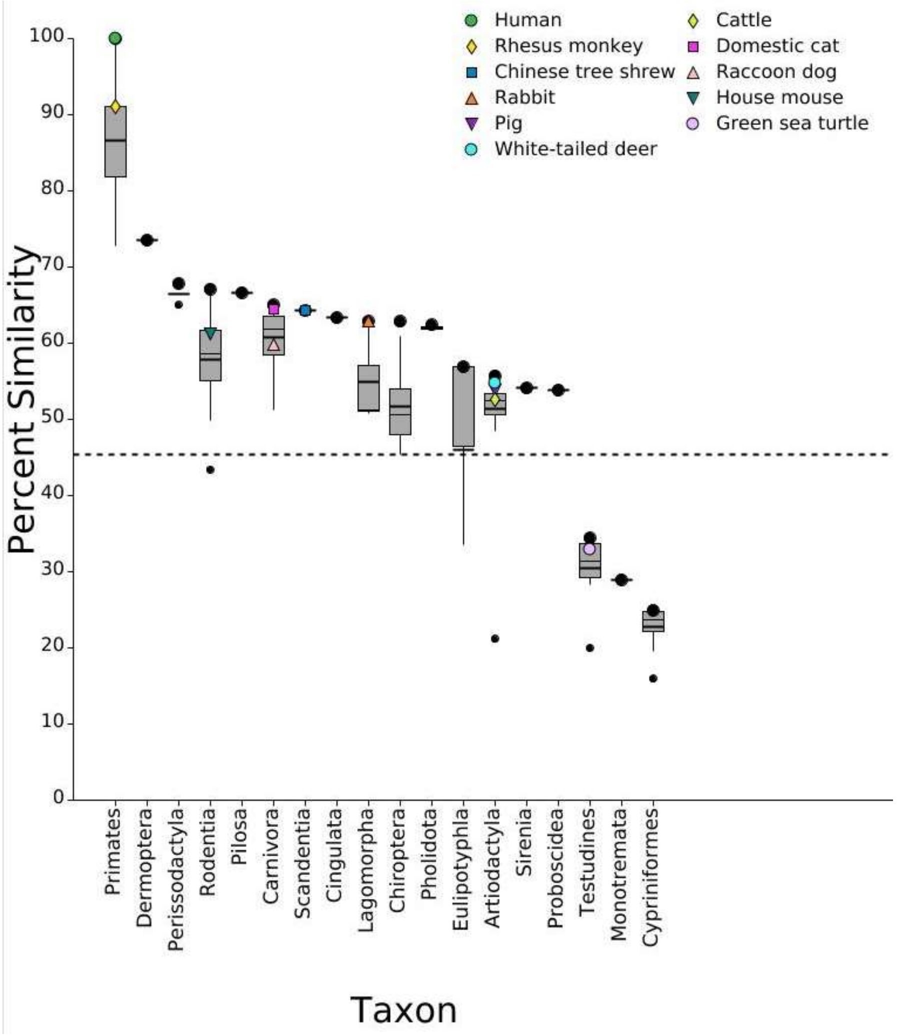

ISG15 Query Accession: NP_005092.1
Level 2: cd01810 Ubl2_ISG15

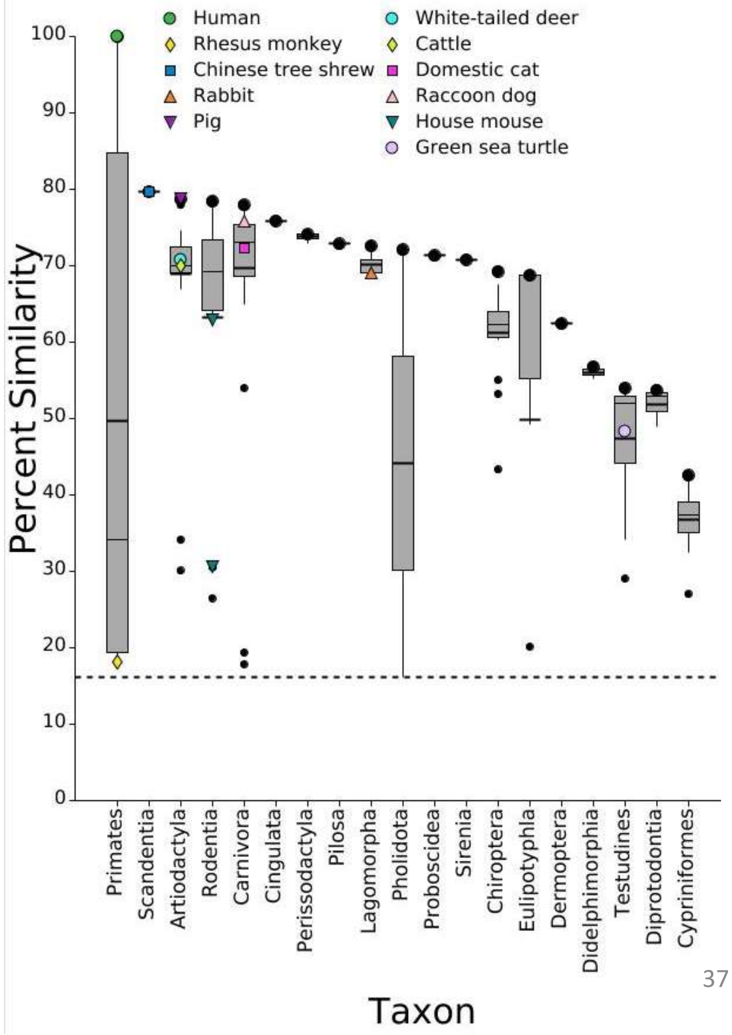

Figure S13c. Ubiquitin-like interferon stimulated gene 15 (ISG15)
Query Accession: NP_005092.1
Level 3: Bond

Total Match
Partial Match
Not a Match

Common Name	Amino Acid 1	Amino Acid 2	Amino Acid 3	Amino Acid 4	Amino Acid 5	Amino Acid 6	Amino Acid 7	Amino Acid 8
Human	22S	123W	127E	128G	130P	153R	155R	156G
Rhesus monkey	22S	123W	127E	128G	130P	153R	155R	156G
Olive baboon	22S	123W	127E	128G	130P	153R	155R	156G
Green monkey	22S	123W	127E	128G	130P	153R	155R	156G
White-tufted-ear marmoset	22S	123W	127E	128G	130P	153R	155R	156G
Chinese tree shrew	22S	123W	127E	128G	130D	153R	155R	156G
Northern tree shrew	22S	123W	127E	128G	130D	153R	155R	156G
Tiger	22N	123W	127E	128G	130P	153R	155R	156G
Puma	22N	123W	127E	128G	130P	153R	155R	156G
Lion	22N	123W	127E	128G	130P	153R	155R	156G
Domestic cat	22N	123W	127E	128G	130P	153R	155R	156G
American mink	22S	123W	127Q	128G	130P	153R	155R	156G
Canada lynx	22N	123W	127E	128G	130P	153R	155R	156G
Fishing cat	22N	123W	127E	128G	130P	153R	155R	156G
Raccoon dog	22S	123W	127E	128G	130P	153R	155R	156G
Pig	22C	122W	126E	127G	129P	152R	154R	155G
Golden hamster	22S	122W	126E	127G	129P	152R	154R	155G
Domestic ferret	22S	123W	127Q	128G	130P	153R	155R	156G
Malayan pangolin	22S	123W	127E	128G	130S	153R	155R	156G
Bobcat	22N	123W	127E	128G	130P	153R	155R	156G
Dog	28S	129W	133E	134G	136P	159R	161R	162G
Eurasian badger	22S	123W	127Q	128G	130P	153R	155R	156G
Gray squirrel	22S	122W	126E	127G	129S	152R	154R	155G
House mouse	22S	121W	125E	126G	128P	151R	153R	154G
Norway rat	22S	122W	126N	127G	129P	152R	154R	155G
Prairie deer mouse	22S	122W	126E	127G	129R	152H	154R	155G
White-tailed deer	24S	125W	129E	130G	132P	155R	157R	158G
Cattle	22S	120W	124E	125G	127P	150R	152R	153G
Big brown bat	21S	122W	126Q	127G	129P	152R	154R	155G
Egyptian rousette	21S	122W	126Q	127G	129V	152R	154R	155G
Western lowland gorilla	96S	194R	198A	199G	201Q	224R	226R	227G
Rock pigeon	375S	478R	482G	483S	485E	508R	510R	511G
Fathead minnow	20N	122R	126N	127G	129Q	152R	154R	155G
Chinese alligator	365S	469R	473N	474S	476E	499R	501R	502G
African clawed frog	35S	133R	137A	138G	140Q	163R	165R	166G

Figure S13c.
Ubiquitin-like interferon stimulated gene 15 (ISG15)
Query Accession: NP_005092.1
Level 3: Distance

Legend: Total Match; Partial Match; Not a Match

Common Name	Amino Acid 1	Amino Acid 2	Amino Acid 3	Amino Acid 4	Amino Acid 5	Amino Acid 6	Amino Acid 7	Amino Acid 8	Amino Acid 9	Amino Acid 10	Amino Acid 11
Human	20S	23M	27E	30A	121L	129K	131L	132E	151N	152L	154L
Rhesus monkey	20S	23M	27E	30A	121L	129K	131L	132E	151N	152L	154L
Olive baboon	20S	23M	27E	30A	121L	129K	131L	132E	151N	152L	154L
Green monkey	20S	23M	27E	30A	121L	129K	131L	132E	151N	152L	154L
White-tufted-ear marmoset	20S	23M	27E	30A	121L	129K	131M	132E	151N	152L	154L
Chinese tree shrew	20S	23M	27E	30Q	121L	129R	131M	132E	151H	152L	154L
Northern tree shrew	20S	23M	27E	30Q	121L	129R	131M	132E	151H	152L	154L
Tiger	20R	23M	27E	30Q	121L	129K	131M	132E	151N	152L	154L
Puma	20R	23M	27E	30Q	121L	129K	131M	132E	151N	152L	154L
Lion	20R	23M	27E	30Q	121L	129K	131M	132E	151N	152L	154L
Domestic cat	20R	23M	27E	30Q	121L	129K	131M	132E	151N	152L	154L
American mink	20R	23M	27E	30Q	121L	129K	131M	132E	151N	152L	154L
Canada lynx	20R	23M	27E	30Q	121L	129K	131M	132E	151N	152L	154L
Fishing cat	20R	23M	27E	30Q	121L	129K	131M	132E	151N	152L	154L
Raccoon dog	20R	23M	27E	30Q	121L	129K	131M	132E	151N	152L	154L
Pig	20R	23M	27D	30Q	120H	128K	130M	131E	150N	151L	153L
Golden hamster	20T	23T	27E	30R	120Q	128R	130M	131E	150N	151L	153L
Domestic ferret	20R	23M	27E	30Q	121L	129K	131M	132E	151N	152L	154L
Malayan pangolin	20Q	23M	27E	30Q	121Q	129K	131L	132E	151N	152L	154L
Bobcat	20R	23M	27E	30Q	121L	129K	131M	132E	151N	152L	154L
Dog	26R	29M	33E	36Q	127L	135K	137M	138E	157N	158L	160L
Eurasian badger	20R	23M	27E	30Q	121L	129K	131M	132D	151N	152L	154L
Gray squirrel	20S	23M	27E	30Q	120M	128R	130M	131E	150N	151L	153L
House mouse	20T	23M	27E	30K	119Q	127R	129M	130E	149H	150L	152L
Norway rat	20T	23M	27E	30K	120Q	128R	130M	131E	150N	151L	153L
Prairie deer mouse	20T	23M	27E	30R	120Q	128R	130M	131E	150N	151L	153L
White-tailed deer	22R	25M	29E	32Q	123Q	131K	133M	134D	153N	154L	156L
Cattle	20R	23M	27E	30Q	118Q	126R	128M	129D	148N	149L	151L
Big brown bat	19R	22M	26E	29Q	120Q	128K	130L	131D	150N	151L	153L
Egyptian rousette	19T	22M	26D	29Q	120Q	128K	130L	131E	150Q	151L	153L
Western lowland gorilla	94E	97D	101N	104A	192Q	200K	202L	203E	222V	223L	225L
Rock pigeon	373S	376T	380K	383E	476Q	484K	486L	487E	506L	507L	509L
Fathead minnow	18S	21A	25E	28Q	120Q	128R	130L	131E	150T	151L	153L
Chinese alligator	363S	366T	370Q	373G	467Q	475Q	477L	478E	497L	498L	500L
African clawed frog	33E	36D	40N	43A	131Q	139K	141L	142E	161V	162L	164L

Figure S14a.
NF-kappa-B essential modulator isoform a (NEMO)
Query Accession: NP_003630.1
Level 1

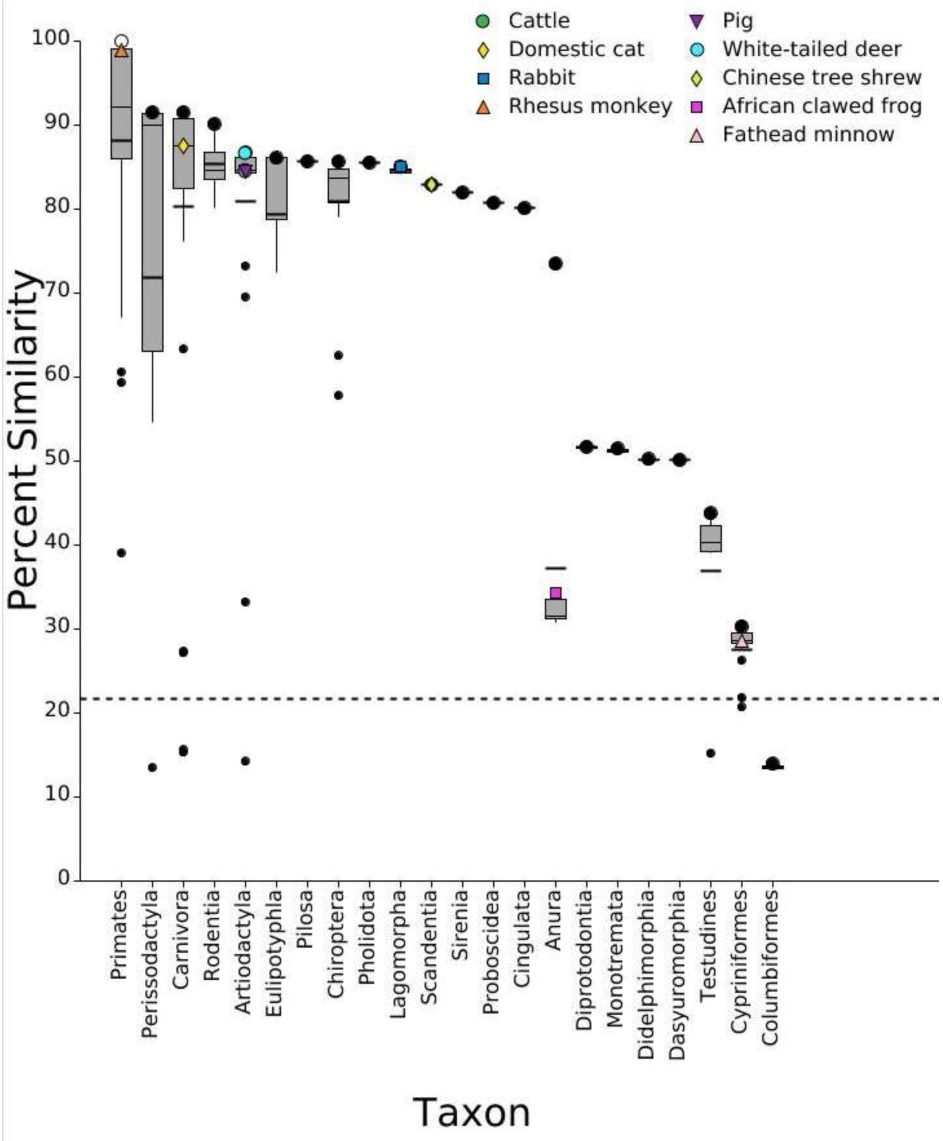

Figure S14b.
NF-kappa-B essential modulator isoform a (NEMO)
Query Accession: NP_003630.1
Level 2: cd09803 UBAN domain

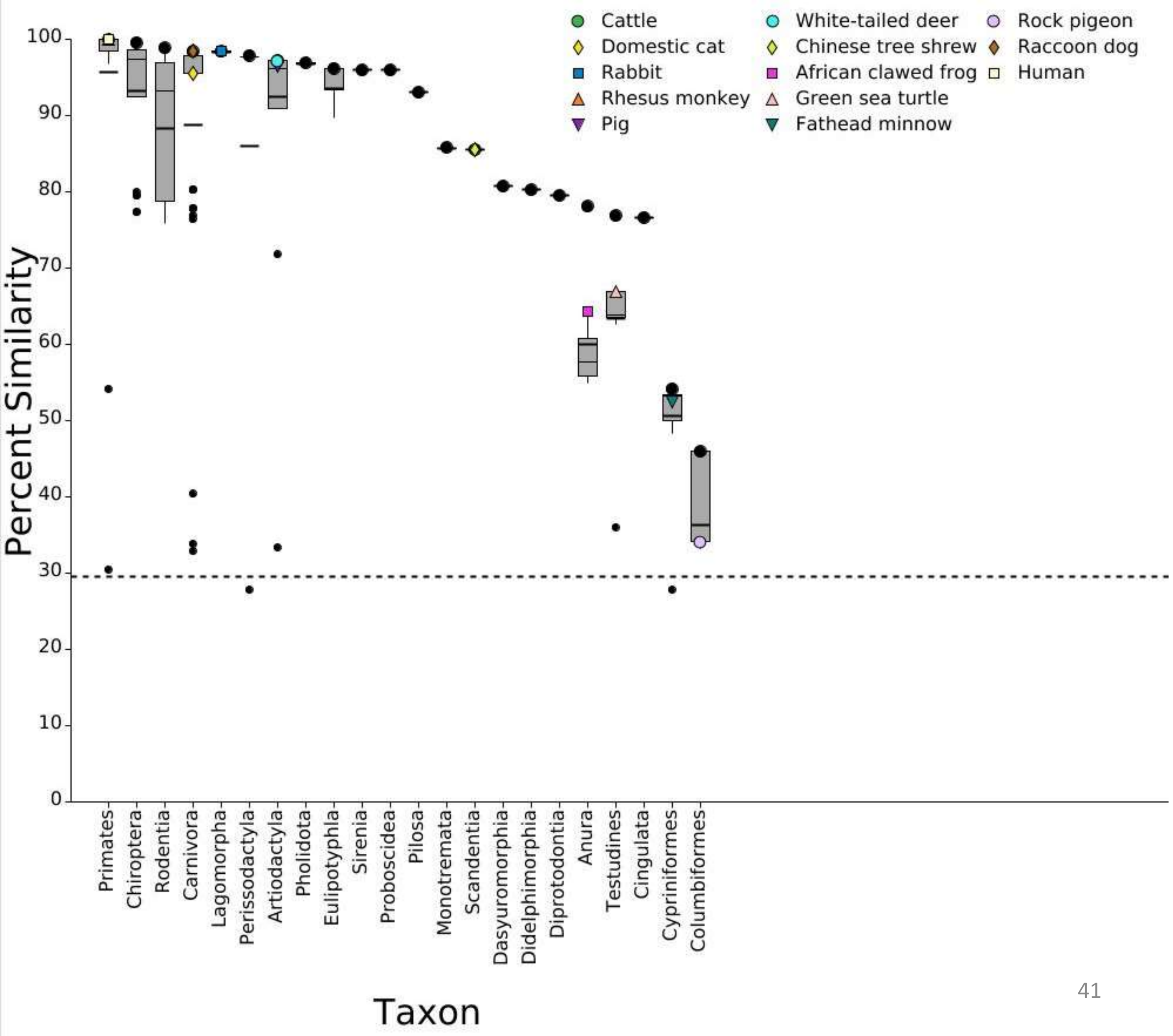

Total Match
Partial Match
Not a Match

Figure S14c. NF-kappa-B essential modulator isoform a (NEMO)
Query Accession: NP_003630.1 Level 3: Bond (Left) and Distance (Right)

Common Name	Amino Acid 1	Amino Acid 2	Amino Acid 3	Amino Acid 4	Amino Acid 5	Amino Acid 6	Amino Acid 7	Amino Acid 8
Human	226K	228A	229Q	230L	231Q	232V	233A	234Y
Western lowland gorilla	226K	228A	229Q	230L	231Q	232V	233A	234Y
Olive baboon	226K	228A	229Q	230L	231Q	232V	233A	234Y
Rhesus monkey	226K	228A	229Q	230L	231Q	232V	233A	234Y
Green monkey	237K	239A	240Q	241L	242Q	243V	244A	245Y
White-tufted-ear marmoset	226K	228A	229Q	230L	231Q	232V	233A	234Y
Gray squirrel	254K	256A	257Q	258L	259Q	260V	261A	262Y
Dog	226K	228A	229Q	230L	231Q	232V	233A	234Y
Red fox	226K	228A	229Q	230L	231Q	232V	233A	234Y
Raccoon dog	264K	266A	267Q	268L	269Q	270V	271A	272Y
Domestic cat	226K	228A	229Q	230L	231Q	232V	233A	234Y
Fishing cat	226K	228A	229Q	230L	231Q	232V	233A	234Y
Canada lynx	226K	228A	229Q	230L	231Q	232V	233A	234Y
Bobcat	226K	228A	229Q	230L	231Q	232V	233A	234Y
White-tailed deer	226K	228A	229Q	230L	231Q	232V	233A	234Y
Puma	225K	227A	228Q	229L	230Q	231V	232A	233Y
Malayan pangolin	226K	228A	229Q	230L	231Q	232V	233A	234Y
Egyptian rousette	226K	228A	229Q	230L	231Q	232V	233A	234Y
Rabbit	226K	228A	229Q	230L	231Q	232V	233A	234Y
Tiger	226K	228A	229Q	230L	231Q	232V	233A	234Y
Cattle	226K	228A	229Q	230L	231Q	232V	233A	234Y
Pig	226K	228A	229Q	230L	231Q	232V	233A	234Y
Prairie deer mouse	226K	228A	229Q	230L	231Q	232A	233A	234Y
House mouse	226K	228A	229Q	230L	231Q	232A	233A	234Y
Norway rat	226K	228A	229Q	230L	231Q	232A	233A	234Y
Chinese tree shrew	226L	228R	229D	230L	231G	232Q	233A	234Y
Golden hamster	226K	228A	229Q	230L	231Q	232A	233A	234Y
Domestic ferret	226K	228A	229Q	230L	231Q	232V	233A	234Y
American mink	226K	228A	229Q	230L	231Q	232V	233A	234Y
Eurasian badger	226K	228A	229Q	230L	231Q	232V	233A	234Y
Lion	379K	381A	382T	383L	384Q	385V	386T	387H
Big brown bat	185K	187A	188Q	189L	190Q	191A	192A	193Y
Chinese alligator	335K	337A	338Q	339L	340Q	341V	342A	343Y
African clawed frog	322K	324A	325Q	326L	327Q	328V	329A	330Y
Fathead minnow	382K	384A	385Q	386L	387Q	388H	389A	390Y

Total Match
Partial Match
Not a Match

Common Name	Amino Acid 1	Amino Acid 2
Human	227L	235H
Western lowland gorilla	227L	235H
Olive baboon	227L	235H
Rhesus monkey	227L	235H
Green monkey	238L	246H
White-tufted-ear marmoset	227L	235H
Gray squirrel	255L	263H
Dog	227L	235H
Red fox	227L	235H
Raccoon dog	265L	273H
Domestic cat	227L	235H
Fishing cat	227L	235H
Canada lynx	227L	235H
Bobcat	227L	235H
White-tailed deer	227L	235H
Puma	226L	234H
Malayan pangolin	227L	235H
Egyptian rousette	227L	235H
Rabbit	227L	235H
Tiger	227L	235H
Cattle	227L	235H
Pig	227L	235H
Prairie deer mouse	227L	235H
House mouse	227L	235H
Norway rat	227L	235H
Chinese tree shrew	227M	235H
Golden hamster	227L	235H
Domestic ferret	227L	235H
American mink	227L	235H
Eurasian badger	227L	235H
Lion	380V	388N
Big brown bat	186L	194H
Chinese alligator	336L	344H
African clawed frog	323L	331H
Fathead minnow	383L	391T

Figure S14c. NF-kappa-B essential modulator isoform a (NEMO)
Query Accession: NP_003630.1
Level 3: UBAN Domain

Total Match
Partial Match
Not a Match

Common Name	Amino Acid 1	Amino Acid 2	Amino Acid 3	Amino Acid 4	Amino Acid 5	Amino Acid 6	Amino Acid 7	Amino Acid 8	Amino Acid 9	Amino Acid 10	Amino Acid 11	Amino Acid 12	Amino Acid 13	Amino Acid 14	Amino Acid 15	Amino Acid 16	Amino Acid 17	Amino Acid 18	Amino Acid 19	Amino Acid 20	Amino Acid 21	Amino Acid 22	Amino Acid 23	Amino Acid 24	Amino Acid 25	Amino Acid 26
Human	296E	297T	299P	300V	301L	302K	303A	304Q	305A	306D	307I	308Y	309K	310A	311D	312F	313Q	314A	315E	316R	317Q	319R	320E	323A	324E	327E
Western lowland gorilla	296E	297T	299P	300V	301L	302K	303A	304Q	305A	306D	307I	308Y	309K	310A	311D	312F	313Q	314A	315E	316R	317Q	319R	320E	323A	324E	327E
Olive baboon	296E	297T	299P	300V	301L	302K	303A	304Q	305A	306D	307I	308Y	309K	310A	311D	312F	313Q	314A	315E	316R	317Q	319R	320E	323A	324E	327E
Rhesus monkey	296E	297T	299P	300V	301L	302K	303A	304Q	305A	306D	307I	308Y	309K	310A	311D	312F	313Q	314A	315E	316R	317Q	319R	320E	323A	324E	327E
Green monkey	307E	308T	310P	311V	312L	313K	314A	315Q	316A	317D	318I	319Y	320K	321A	322D	323F	324Q	325A	326E	327R	328Q	330R	331E	334A	335E	338E
White-tufted-ear marmoset	296E	297T	299P	300V	301L	302K	303A	304Q	305A	306D	307I	308Y	309K	310A	311D	312F	313Q	314A	315E	316R	317Q	319R	320E	323A	324E	327E
Gray squirrel	324E	325T	327P	328V	329L	330K	331A	332Q	333A	334D	335I	336Y	337K	338A	339D	340F	341Q	342A	343E	344R	345Q	347R	348E	351A	352E	355E
Dog	296E	297T	299P	300V	301L	302K	303A	304Q	305A	306D	307I	308Y	309K	310A	311D	312F	313Q	314A	315E	316R	317Q	319R	320E	323A	324E	327E
Red fox	296E	297T	299P	300V	301L	302K	303A	304Q	305A	306D	307I	308Y	309K	310A	311D	312F	313Q	314A	315E	316R	317Q	319R	320E	323A	324E	327E
Raccoon dog	334E	335T	337P	338V	339L	340K	341A	342Q	343A	344D	345I	346Y	347K	348A	349D	350F	351Q	352A	353E	354R	355Q	357R	358E	361A	362E	365E
Domestic cat	296E	297T	299P	300V	301L	302K	303A	304Q	305A	306D	307I	308Y	309K	310A	311D	312F	313Q	314A	315E	316R	317Q	319R	320E	323A	324E	327E
Fishing cat	296E	297T	299P	300V	301L	302K	303A	304Q	305A	306D	307I	308Y	309K	310A	311D	312F	313Q	314A	315E	316R	317Q	319R	320E	323A	324E	327E
Canada lynx	296E	297T	299P	300V	301L	302K	303A	304Q	305A	306D	307I	308Y	309K	310A	311D	312F	313Q	314A	315E	316R	317Q	319R	320E	323A	324E	327E
Bobcat	296E	297T	299P	300V	301L	302K	303A	304Q	305A	306D	307I	308Y	309K	310A	311D	312F	313Q	314A	315E	316R	317Q	319R	320E	323A	324E	327E
White-tailed deer	296E	297T	299P	300V	301L	302K	303A	304Q	305A	306D	307I	308Y	309K	310A	311D	312F	313Q	314A	315E	316R	317Q	319R	320E	323A	324E	327E
Puma	295E	296T	298P	299V	300L	301K	302A	303Q	304A	305D	306I	307Y	308K	309A	310D	311F	312Q	313A	314E	315R	316Q	318R	319E	322A	323E	326E
Malayan pangolin	296E	297T	299P	300V	301L	302K	303A	304Q	305A	306D	307I	308Y	309K	310A	311D	312F	313Q	314A	315E	316R	317Q	319R	320E	323A	324E	327E
Egyptian rousette	296E	297T	299P	300V	301L	302K	303A	304Q	305A	306D	307I	308Y	309K	310A	311D	312F	313Q	314A	315E	316R	317Q	319R	320E	323A	324E	327E
Rabbit	296E	297T	299P	300V	301L	302K	303A	304Q	305A	306D	307I	308Y	309K	310A	311D	312F	313Q	314A	315E	316R	317Q	319R	320E	323A	324E	327E
Tiger	296E	297T	299P	300V	301L	302K	303A	304Q	305A	306D	307I	308Y	309K	310A	311D	312F	313Q	314A	315E	316R	317Q	319R	320E	323A	324E	327E
Cattle	296E	297T	299P	300V	301L	302K	303A	304Q	305A	306D	307I	308Y	309K	310A	311D	312F	313Q	314A	315E	316R	317Q	319R	320E	323A	324E	327E
Pig	296E	297T	299P	300V	301L	302K	303A	304Q	305A	306D	307I	308Y	309K	310A	311D	312F	313Q	314A	315E	316R	317Q	319R	320E	323A	324E	327E
Prairie deer mouse	296E	297T	299P	300V	301L	302K	303A	304Q	305A	306D	307I	308Y	309K	310A	311D	312F	313Q	314A	315E	316R	317H	319R	320E	323V	324E	327E
House mouse	289E	290T	292P	293V	294L	295K	296A	297Q	298A	299D	300I	301Y	302K	303A	304D	305F	306Q	307A	308E	309R	310H	312R	313E	316V	317E	320E
Norway rat	289E	290T	292P	293V	294L	295K	296A	297Q	298A	299D	300I	301Y	302K	303A	304D	305F	306Q	307A	308E	309R	310H	312R	313E	316V	317E	320E
Chinese tree shrew	296E	297T	299P	300V	301L	302R	303A	304Q	305A	306D	307I	308Y	309K	310A	311D	312F	313Q	314A	315E	316R	317Q	319R	320E	323A	324E	327E
Golden hamster	289E	290T	292P	293V	294L	295K	296A	297Q	298A	299D	300I	301Y	302K	303A	304D	305F	306Q	307A	308E	309R	310H	312R	313E	316V	317E	320E
Domestic ferret	289E	290T	292P	293V	294L	295K	296A	297Q	298A	299D	300I	301Y	302K	303A	304D	305F	306Q	307A	308E	309R	310Q	312R	313E	316A	317E	320E
American mink	289E	290T	292P	293V	294L	295K	296A	297Q	298A	299D	300I	301Y	302K	303A	304D	305F	306Q	307A	308E	309R	310Q	312R	313E	316A	317E	320E
Eurasian badger	289E	290T	292P	293V	294L	295K	296A	297Q	298A	299D	300I	301Y	302K	303A	304D	305F	306Q	307A	308E	309R	310Q	312R	313E	316A	317E	320E
Lion	453E	454T	456T	457V	458L	459R	460A	461Q	462M	463E	464V	465Y	466C	467S	468D	469F	470H	471A	472E	473R	474A	476R	477E	480H	481E	484E
Big brown bat	255E	256T	258P	259V	260L	261K	262A	263Q	264A	265D	266I	267Y	268E	269A	270D	271F	272Q	273A	274E	275R	276Q	278R	279E	282A	283E	286E
Chinese alligator	407E	408T	410P	411V	412L	413K	414A	415Q	416A	417D	418I	419Y	420K	421A	422D	423F	424L	425A	426E	427R	428Q	430R	431E	434H	435G	438E
African clawed frog	394D	395T	397P	398V	399L	400K	401A	402Q	403V	404E	405I	406Y	407R	408A	409D	410F	411L	412A	413E	414R	415K	417R	418E	421H	422A	425E
Fathead minnow	450E	451T	453P	454V	455L	456N	457A	458Q	459A	460E	461I	462F	463K	464M	465D	466F	467L	468A	469E	470R	471E	473R	474E	477N	478Q	481E

Figure S15a.
DNA polymerase alpha 1, catalytic subunit (POLA1)
Query Accession: NP_058633.2
Level 1

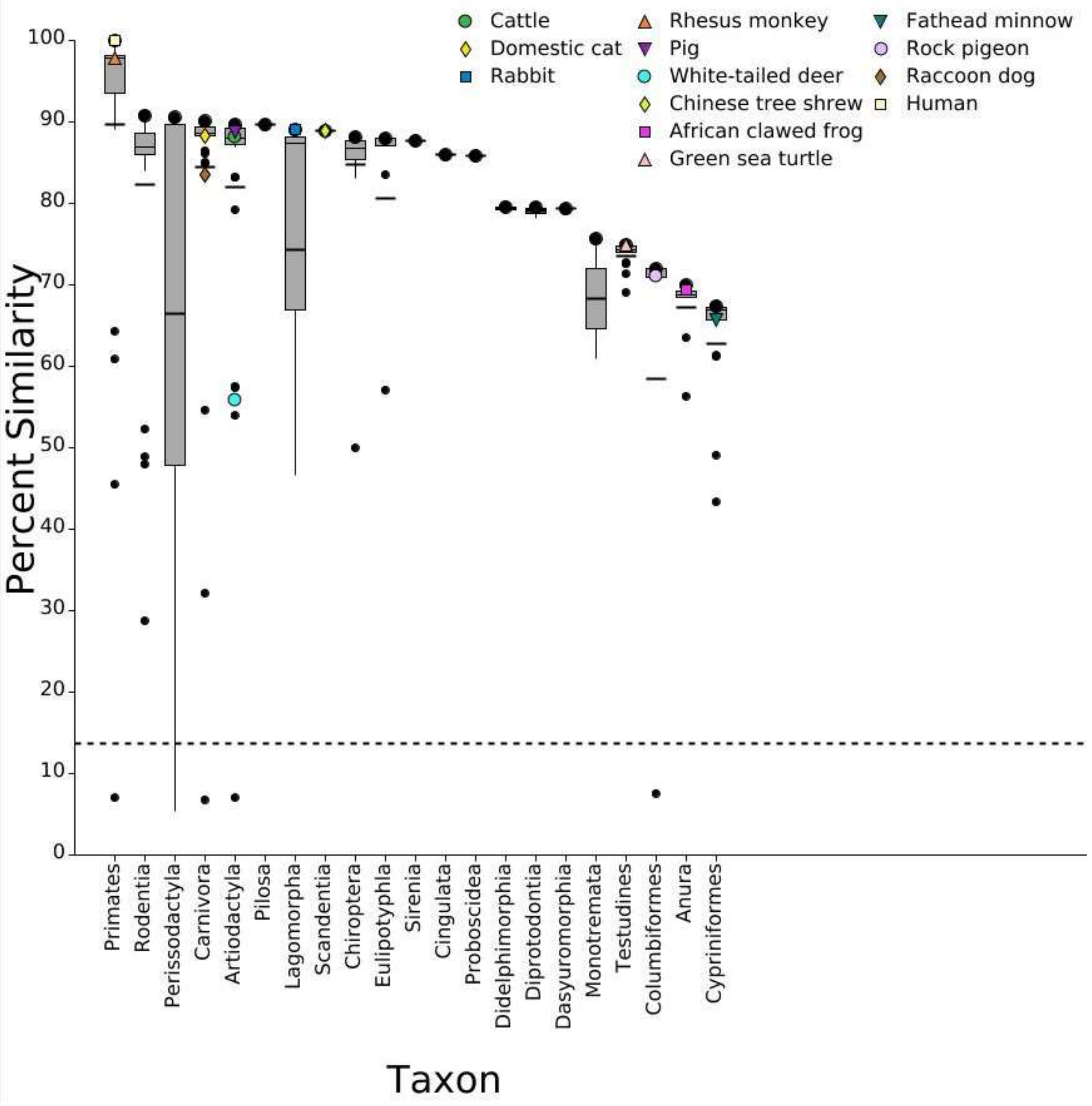

Figure S15b. DNA polymerase alpha 1, catalytic subunit (POLA1) Query Accession: NP_058633.2
Level 2: cd05776 DNA_polB_alpha_exo

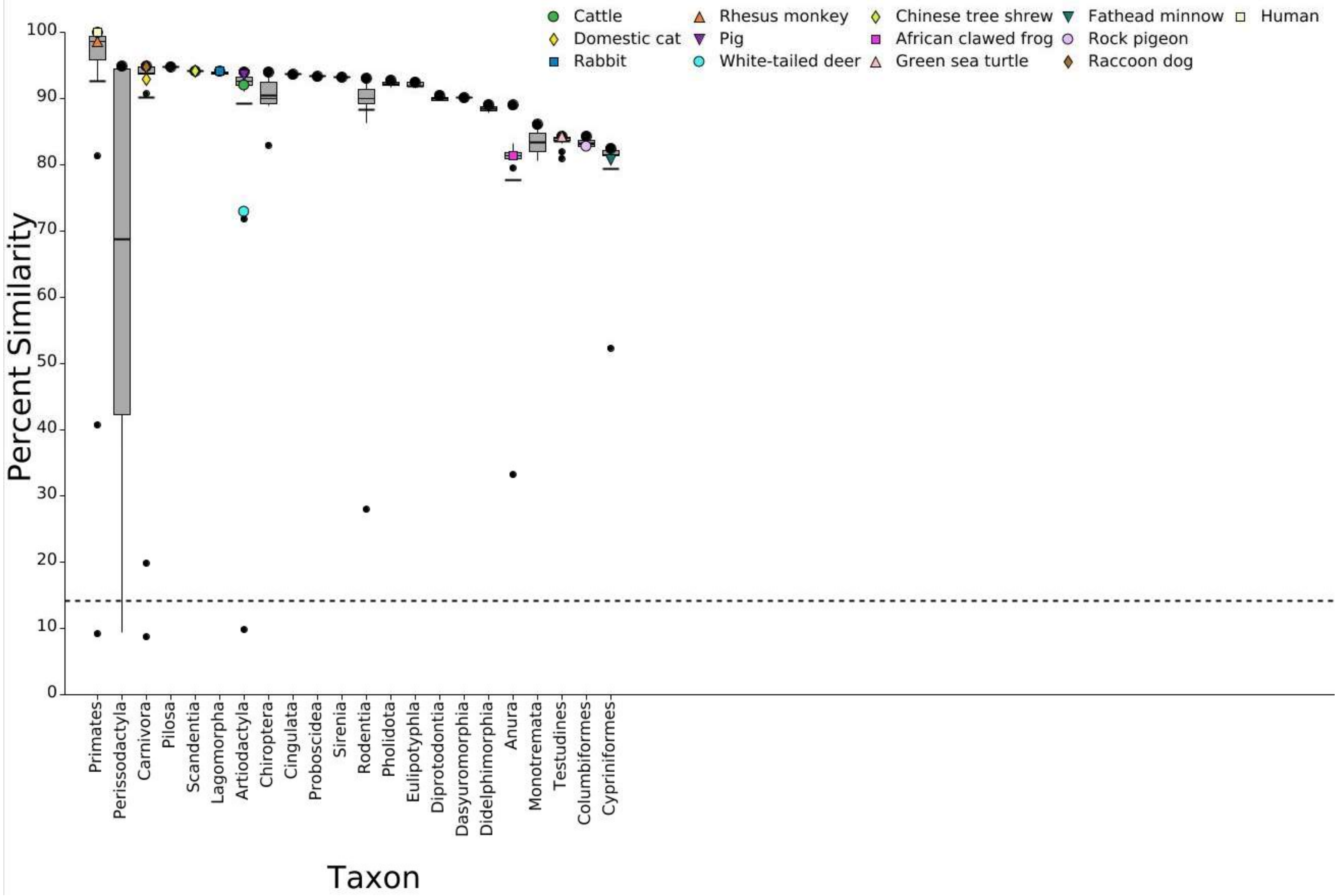

Figure S15c. DNA polymerase alpha 1, catalytic subunit (POLA1) Query Accession: NP_058633.2

Level 3: Bond (Left) and Distance (Right)

(Note lion sequence correction, Supplementary File 4.)

Total Match
Partial Match
Not a Match

Common Name	Amino Acid 1	Amino Acid 2	Amino Acid 3	Amino Acid 4	Amino Acid 5
Human	610E	613A	616R	617T	655K
Western lowland gorilla	610E	613A	616R	617T	655K
Green monkey	671E	674A	677R	678T	716K
Rhesus monkey	616E	619A	622R	623T	661K
Olive baboon	613E	616A	619R	620T	658K
White-tufted-ear marmoset	610E	613A	616R	617T	655K
Red fox	616E	619A	622R	623T	661K
Gray squirrel	613E	616T	619R	620T	658K
Dog	616E	619A	622R	623T	661K
Eurasian badger	616E	619A	622R	623T	661K
Puma	610E	613A	616R	617T	655K
Chinese tree shrew	613E	616A	619R	620T	658K
Pig	616E	619A	622R	623T	661K
Tiger	616E	619A	622R	623T	661K
Bobcat	616E	619A	622R	623T	661K
Canada lynx	616E	619A	622R	623T	661K
American mink	616E	619A	622R	623T	661K
Domestic ferret	616E	619A	622R	623T	661K
Fishing cat	616E	619A	622R	623T	661K
Domestic cat	616E	619A	622R	623T	661K
Cattle	610E	613A	616R	617T	655K
Egyptian rousette	608E	611A	614R	615T	653K
Norway rat	617E	620A	623R	624T	662K
Malayan pangolin	613E	616A	619R	620T	658K
Golden hamster	616E	619A	622R	623T	661K
Prairie deer mouse	615E	618A	621R	622T	660K
House mouse	614E	617A	620R	621T	659K
Raccoon dog	616E	619A	622R	623T	661K
Lion	369Q	372E	375E	376E	414K
Big brown bat	610E	613A	616R	617T	655K
Rock pigeon	629E	632A	635R	636T	674K
Chinese alligator	626E	629A	632R	633T	671K
African clawed frog	610E	613L	616R	617T	655K
Fathead minnow	622E	625G	628R	629A	667K

Total Match
Partial Match
Not a Match

Common Name	Amino Acid 1	Amino Acid 2	Amino Acid 3	Amino Acid 4	Amino Acid 5	Amino Acid 6	Amino Acid 7	Amino Acid 8
Human	594F	611V	614T	620G	621F	624A	625K	657P
Western lowland gorilla	594F	611V	614T	620G	621F	624A	625K	657P
Green monkey	655F	672V	675T	681G	682F	685A	686K	718P
Rhesus monkey	600F	617V	620T	626G	627F	630A	631K	663P
Olive baboon	597F	614V	617T	623G	624F	627A	628K	660P
White-tufted-ear marmoset	594F	611V	614T	620G	621F	624A	625N	657P
Red fox	600F	617I	620T	626G	627F	630A	631K	663P
Gray squirrel	597F	614V	617T	623G	624F	627A	628K	660P
Dog	600F	617I	620T	626G	627F	630A	631K	663P
Eurasian badger	600F	617I	620T	626G	627F	630A	631K	663P
Puma	594F	611I	614T	620G	621F	624A	625K	657P
Chinese tree shrew	597F	614V	617T	623G	624F	627A	628K	660P
Pig	600F	617V	620T	626G	627F	630A	631K	663P
Tiger	600F	617I	620T	626G	627F	630A	631K	663P
Bobcat	600F	617I	620T	626G	627F	630A	631K	663P
Canada lynx	600F	617I	620T	626G	627F	630A	631K	663P
American mink	600F	617I	620T	626G	627F	630A	631K	663P
Domestic ferret	600F	617I	620T	626G	627F	630A	631K	663P
Fishing cat	600F	617I	620T	626G	627F	630A	631K	663P
Domestic cat	600F	617I	620T	626G	627F	630A	631K	663P
Cattle	594F	611V	614T	620G	621F	624A	625K	657P
Egyptian rousette	592F	609V	612T	618G	619F	622A	623K	655P
Norway rat	601F	618V	621T	627G	628F	631A	632K	664P
Malayan pangolin	597L	614V	617T	623G	624F	627A	628K	660P
Golden hamster	600F	617V	620T	626G	627F	630A	631K	663P
Prairie deer mouse	599F	616I	619T	625G	626F	629A	630K	662P
House mouse	598F	615I	618T	624G	625F	628A	629K	661P
Raccoon dog	600F	617I	620T	626G	627F	630A	631K	663P
Lion	364L	370S	373R	379Q	380A	383T	384F	419P
Big brown bat	594F	611I	614T	620G	621F	624A	625K	657P
Rock pigeon	613F	630I	633T	639G	640F	643A	644K	676P
Chinese alligator	610F	627I	630T	636G	637F	640A	641K	673P
African clawed frog	594F	611I	614T	620G	621F	624A	625K	657P
Fathead minnow	606F	623I	626T	632G	633F	636A	637K	669P

Figure S16a. Ribonucleic acid export 1 (RAE1)
Query Accession: NP_003601.1
Level 1

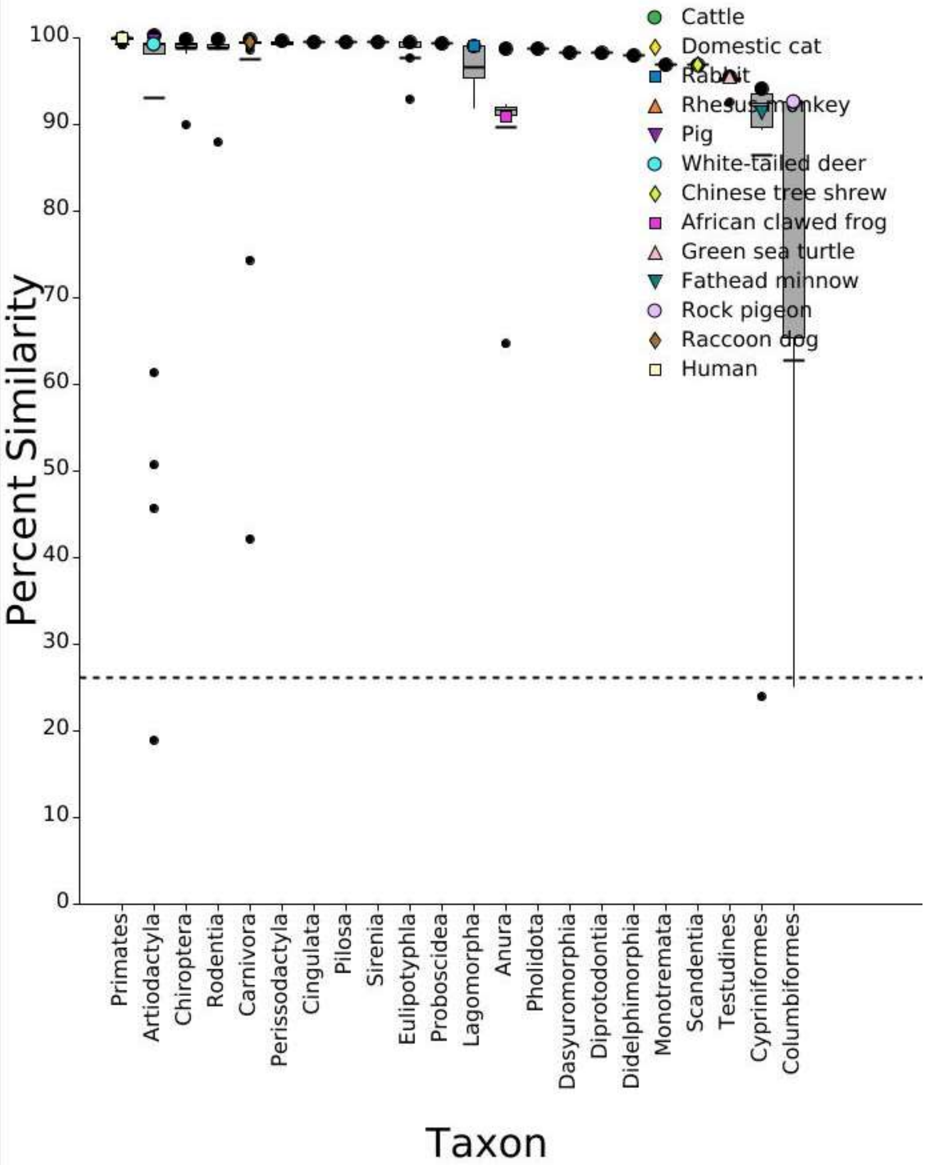

Figure S16b.
Ribonucleic acid export 1 (RAE1)
Query Accession: NP_003601.1
Level 3: Bond

Total Match
Partial Match
Not a Match

Common Name	Amino Acid 1	Amino Acid 2	Amino Acid 3	Amino Acid 4	Amino Acid 5	Amino Acid 6	Amino Acid 7	Amino Acid 8	Amino Acid 9
Human	239R	256T	257F	258K	305R	306T	307K	309K	310T
Rhesus monkey	239R	256T	257F	258K	305R	306T	307K	309K	310T
Olive baboon	239R	256T	257F	258K	305R	306T	307K	309K	310T
White-tufted-ear marmoset	247R	264T	265F	266K	313R	314T	315K	317K	318T
Gray squirrel	296R	313T	314F	315K	362R	363T	364K	366K	367T
Western lowland gorilla	239R	256T	257F	258K	305R	306T	307K	309K	310T
Green monkey	239R	256T	257F	258K	305R	306T	307K	309K	310T
Tiger	254R	271T	272F	273K	320R	321T	322K	324K	325T
Lion	254R	271T	272F	273K	320R	321T	322K	324K	325T
Pig	239R	256T	257F	258K	305R	306T	307K	309K	310T
Domestic cat	239R	256T	257F	258K	305R	306T	307K	309K	310T
Puma	239R	256T	257F	258K	305R	306T	307K	309K	310T
Raccoon dog	239R	256T	257F	258K	305R	306T	307K	309K	310T
Canada lynx	239R	256T	257F	258K	305R	306T	307K	309K	310T
Fishing cat	239R	256T	257F	258K	305R	306T	307K	309K	310T
Dog	239R	256T	257F	258K	305R	306T	307K	309K	310T
Red fox	239R	256T	257F	258K	305R	306T	307K	309K	310T
Bobcat	239R	256T	257F	258K	305R	306T	307K	309K	310T
Domestic ferret	247R	264T	265F	266K	313R	314T	315K	317K	318T
Eurasian badger	239R	256T	257F	258K	305R	306T	307K	309K	310T
White-tailed deer	239R	256T	257F	258K	305R	306T	307K	309K	310T
American mink	239R	256T	257F	258K	305R	306T	307K	309K	310T
Cattle	239R	256T	257F	258K	305R	306T	307K	309K	310T
House mouse	239R	256T	257F	258K	305R	306T	307K	309K	310T
Golden hamster	239R	256T	257F	258K	305R	306T	307K	309K	310T
Prairie deer mouse	239R	256T	257F	258K	305R	306T	307K	309K	310T
Norway rat	243R	260T	261F	262K	309R	310T	311K	313K	314T
Malayan pangolin	238R	255T	256F	257K	304R	305T	306K	308K	309T
Egyptian rousette	239R	256T	257F	258K	305R	306T	307K	309K	310T
Big brown bat	239R	256T	257F	258K	305R	306T	307K	309K	310T
Rock pigeon	239R	256T	257F	258K	305R	306T	307K	309K	310T
Chinese alligator	229R	246T	247F	248K	295R	296T	297K	299K	300T
Fathead minnow	239R	256T	257F	258K	305R	306T	307K	309K	310T
African clawed frog	239R	256T	257F	258K	305R	306T	307K	309K	310T

Figure S16b.
Ribonucleic acid export 1 (RAE1)
Query Accession: NP_003601.1
Level 3: Distance

Total Match
Partial Match
Not a Match

Common Name	Amino Acid 1	Amino Acid 2	Amino Acid 3	Amino Acid 4	Amino Acid 5	Amino Acid 6
Human	255F	300W	301D	302K	308L	365R
Rhesus monkey	255F	300W	301D	302K	308L	365R
Olive baboon	255F	300W	301D	302K	308L	365R
White-tufted-ear marmoset	263F	308W	309D	310K	316L	373R
Gray squirrel	312F	357W	358D	359K	365L	422R
Western lowland gorilla	255F	300W	301D	302K	308L	365R
Green monkey	255F	300W	301D	302K	308L	365R
Tiger	270F	315W	316D	317K	323L	380R
Lion	270F	315W	316D	317K	323L	380R
Pig	255F	300W	301D	302K	308L	365R
Domestic cat	255F	300W	301D	302K	308L	365R
Puma	255F	300W	301D	302K	308L	365R
Raccoon dog	255F	300W	301D	302K	308L	365R
Canada lynx	255F	300W	301D	302K	308L	365R
Fishing cat	255F	300W	301D	302K	308L	365R
Dog	255F	300W	301D	302K	308L	365R
Red fox	255F	300W	301D	302K	308L	365R
Bobcat	255F	300W	301D	302K	308L	365R
Domestic ferret	263F	308W	309D	310K	316L	373R
Eurasian badger	255F	300W	301D	302K	308L	365R
White-tailed deer	255F	300W	301D	302K	308L	365R
American mink	255F	300W	301D	302K	308L	365R
Cattle	255F	300W	301D	302K	308L	365R
House mouse	255F	300W	301D	302K	308L	365R
Golden hamster	255F	300W	301D	302K	308L	365R
Prairie deer mouse	255F	300W	301D	302K	308L	365R
Norway rat	259F	304W	305D	306K	312L	369R
Malayan pangolin	254F	299W	300D	301K	307L	364R
Egyptian rousette	255F	300W	301D	302K	308L	365R
Big brown bat	255F	300W	301D	302K	308L	365R
Rock pigeon	255F	300W	301D	302K	308L	365R
Chinese alligator	245F	290W	291D	292K	298L	355R
African clawed frog	255F	300W	301D	302K	308L	365R
Fathead minnow	255F	300W	301D	302K	308L	365R

Figure S17a.
Retinoic acid-inducible gene I (RIG-I)
Query Accession: NP_055129.2
Level 1

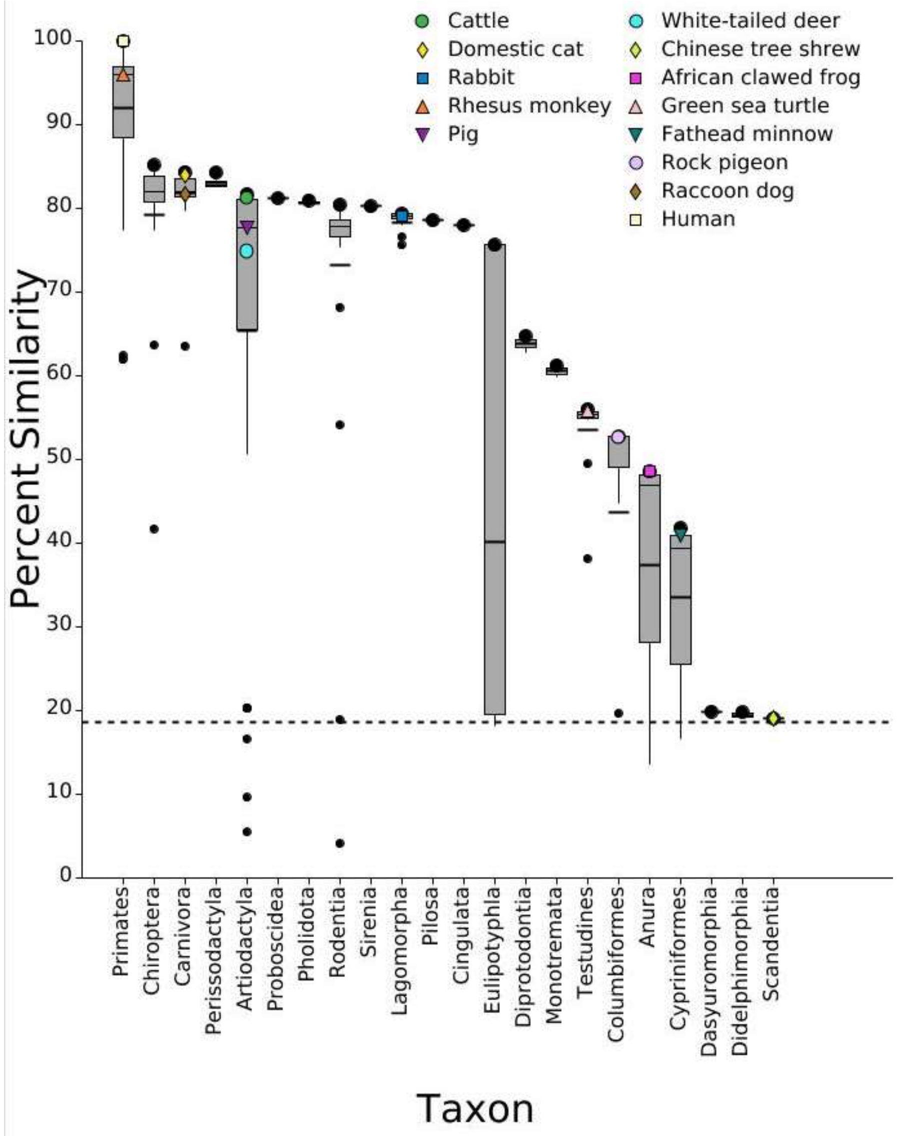

Figure S17b. Retinoic acid-inducible gene I (RIG-I)
Level 2: cd08816: CARD_RIG-I_r1 (N-terminal Caspase activation and recruitment domain found in RIG-I, first repeat)

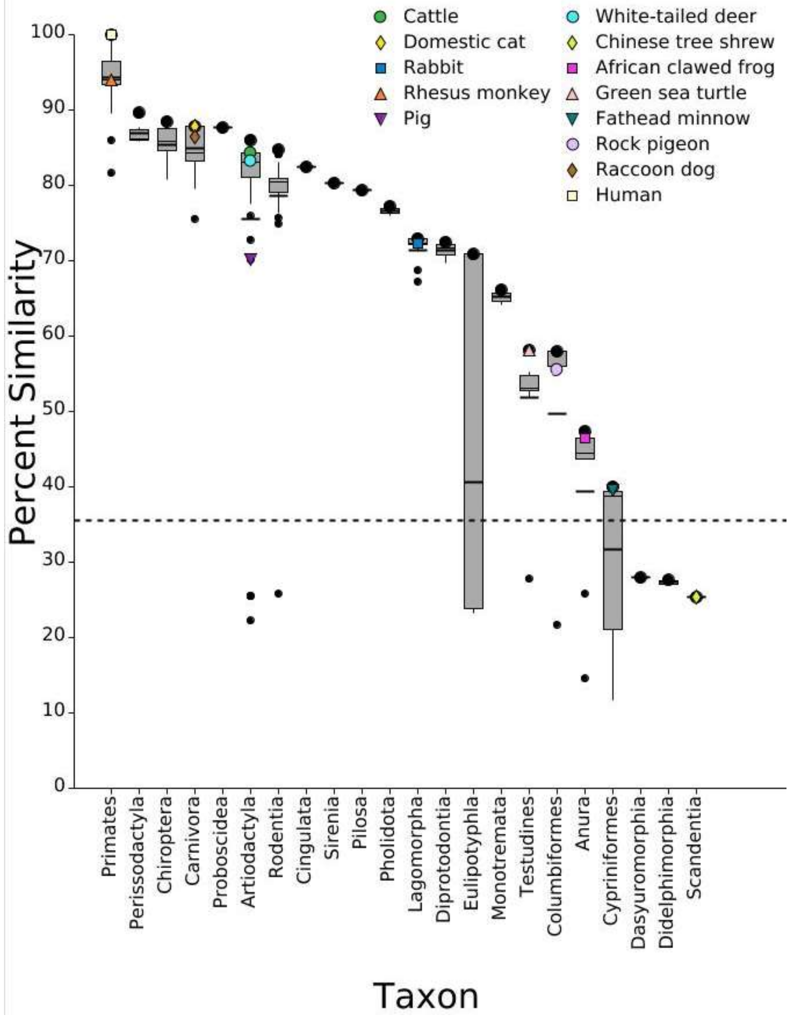

RIG-I Level 2: cd08817: CARD_RIG-I_r2 (second repeat)

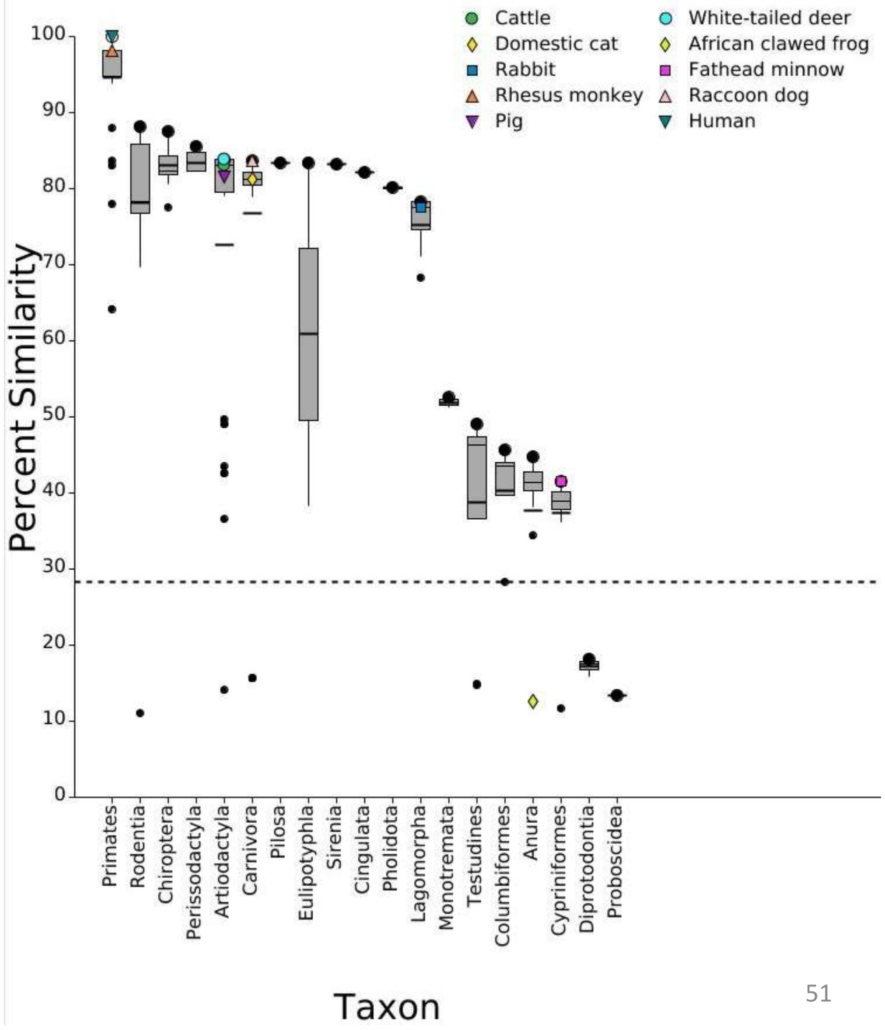

Figure S18. Signal recognition particle 19 (SRP19)
Query Accession: NP_003126.1
Level 1

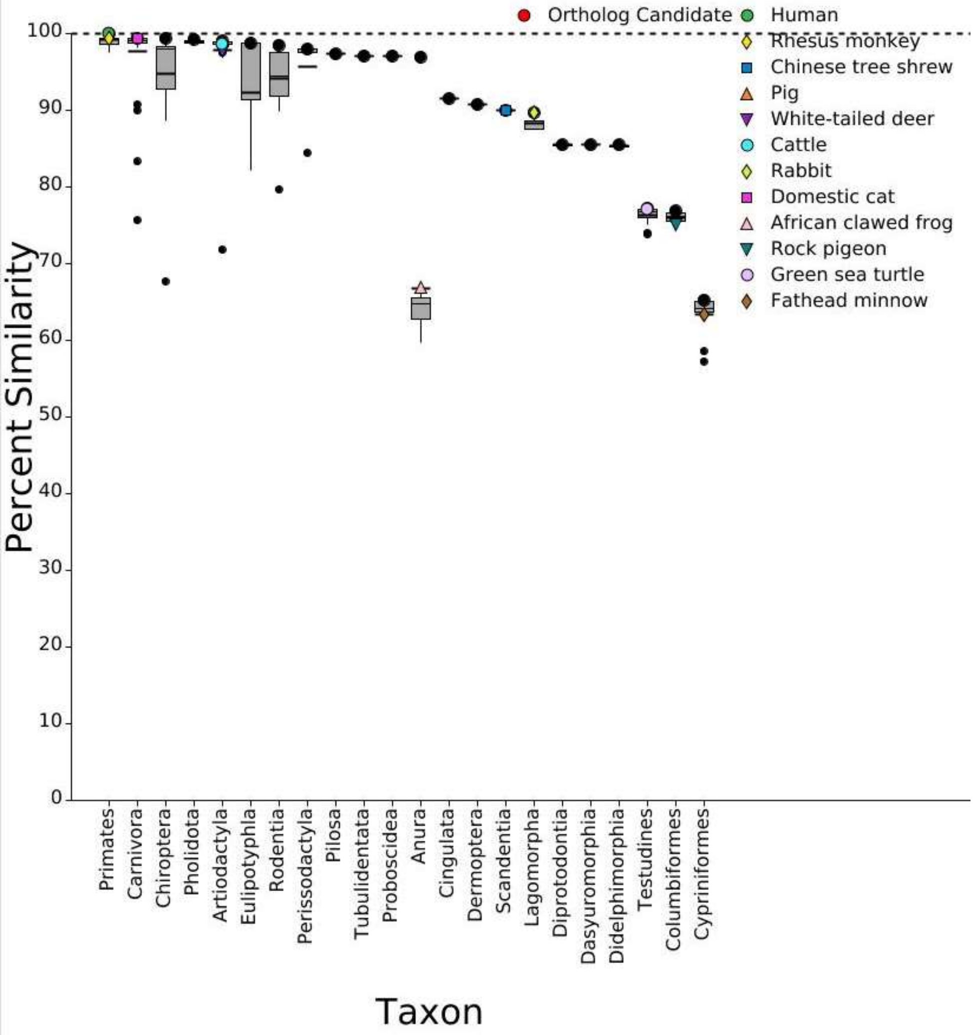

Figure S19. Signal recognition particle 54 (SRP54)
Query Accession: NP_003127.1
Level 1

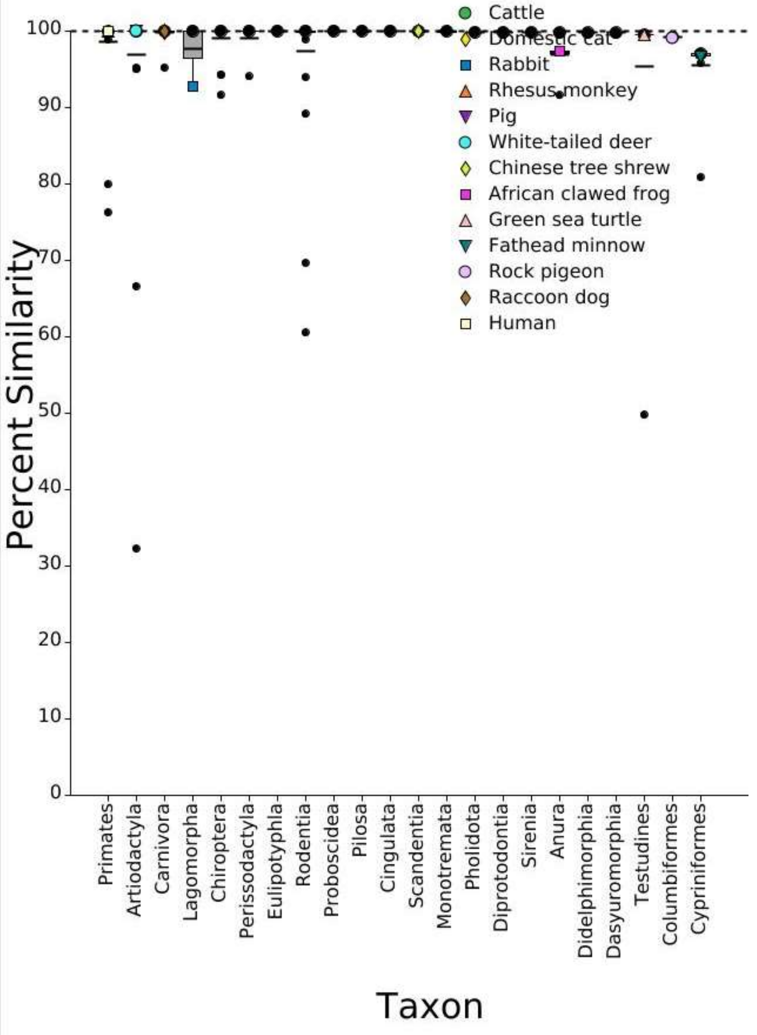

Figure S20a. Signal transducer and activator of transcription 1 (STAT1)
Query Accession: NP_009330.1
Level 1

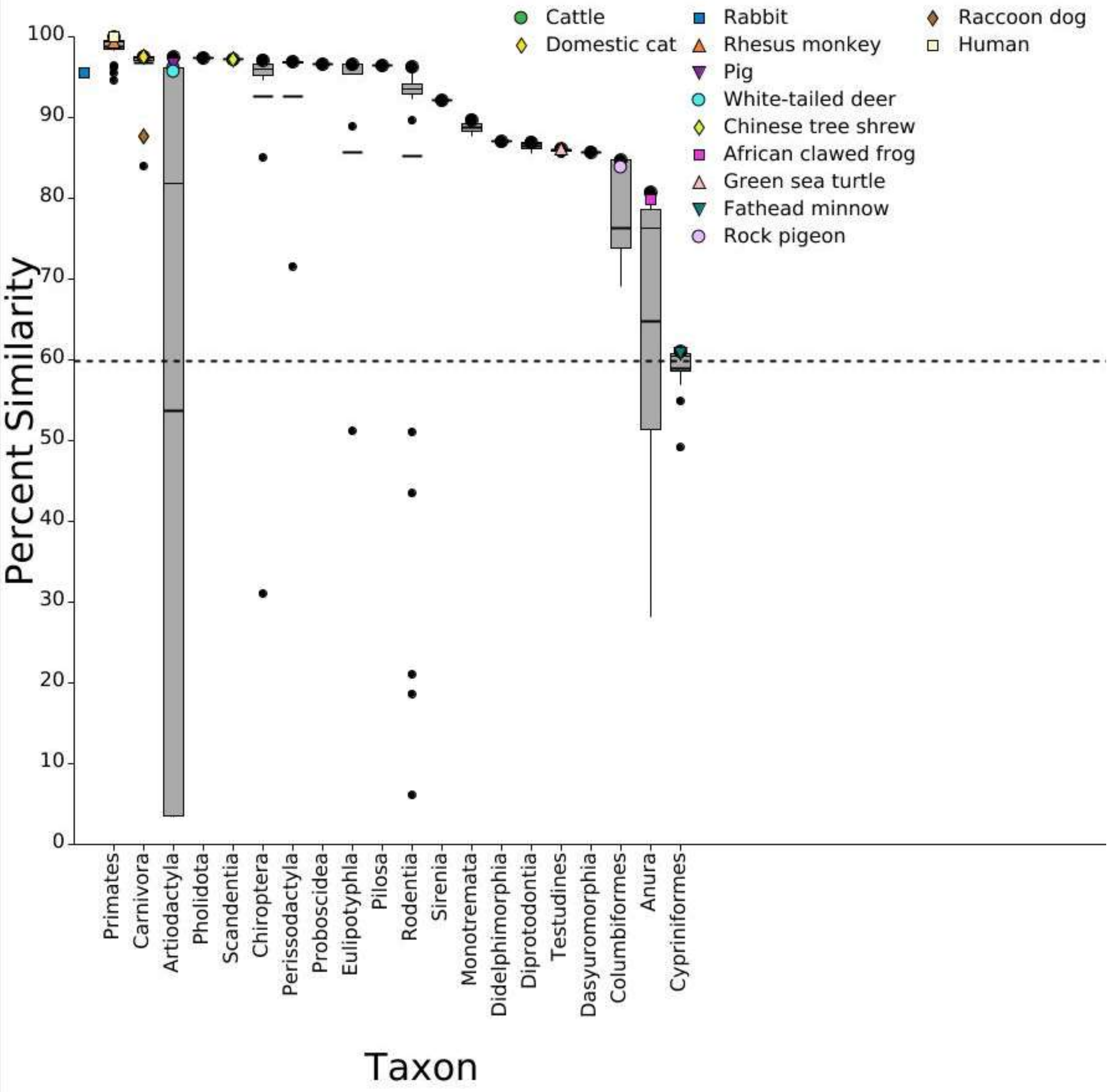

Figure S20b. Signal transducer and activator of transcription 1 (STAT1) Query Accession: NP_009330.1
Level 2: cd10372 SH2_STAT1 Src homology 2 (SH2) domain found in STAT1 proteins

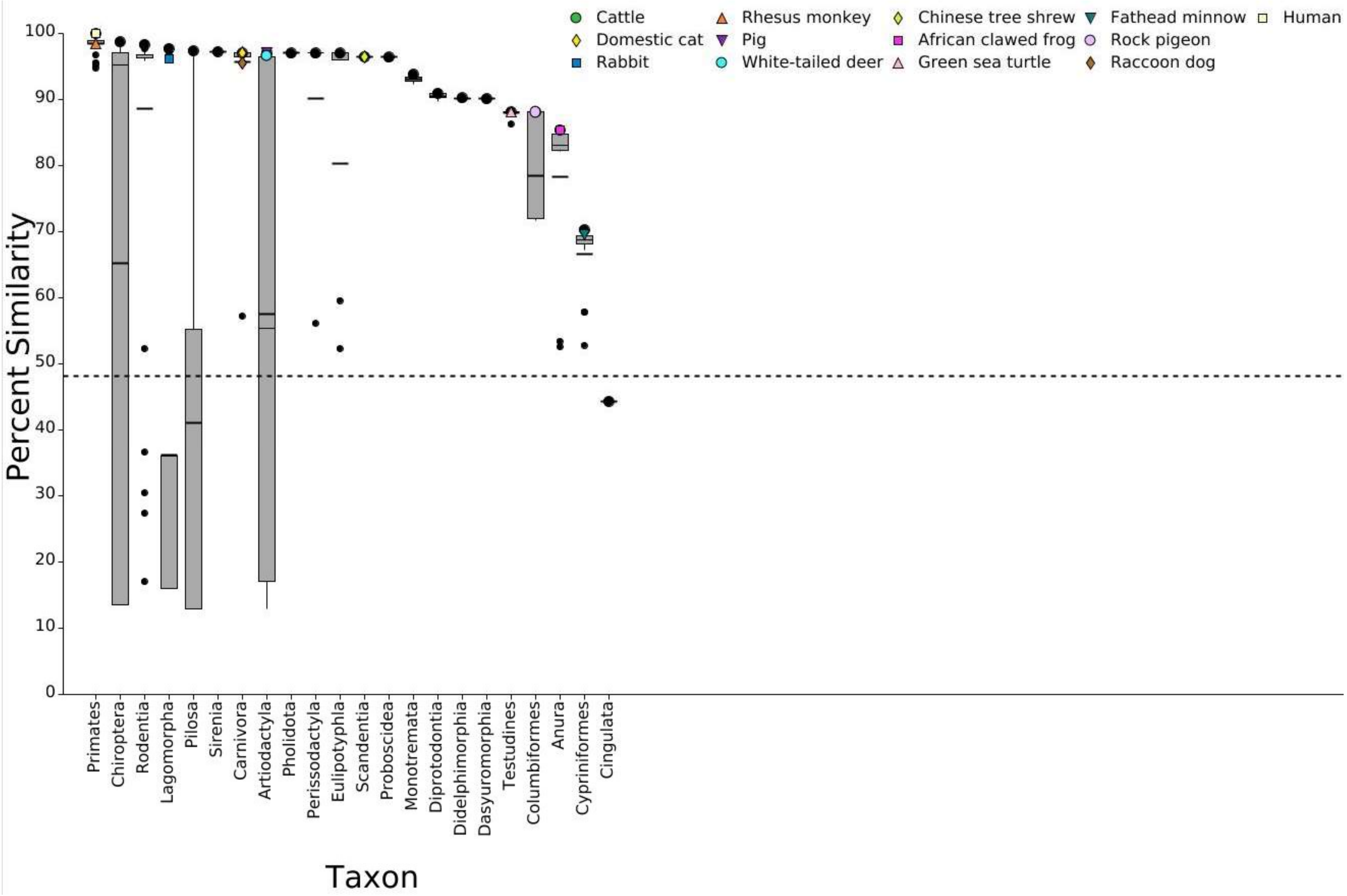

Figure S20c. Signal transducer and activator of transcription 1 (STAT1) Query Accession: NP_009330.1 Level 3: Bond (Left) and Distance (Right)

Total Match
Partial Match
Not a Match

Common Name	Amino Acid 1	Amino Acid 2	Amino Acid 3	Amino Acid 4	Amino Acid 5	Amino Acid 6	Amino Acid 7
Human	628F	629H	630A	631V	632E	651Y	652K
Big brown bat	628F	629H	630A	631V	632E	651Y	652K
Western lowland gorilla	628F	629H	630A	631V	632E	651Y	652K
Rhesus monkey	628F	629H	630A	631V	632E	651Y	652K
Green monkey	628F	629H	630A	631V	632E	651Y	652K
Olive baboon	628F	629H	630A	631V	632E	651Y	652K
White-tufted-ear marmoset	628F	629H	630A	631V	632E	651Y	652K
Domestic cat	628F	629H	630A	631V	632E	651Y	652K
Lion	628F	629H	630A	631V	632E	651Y	652K
Tiger	628F	629H	630A	631V	632E	651Y	652K
Puma	628F	629H	630A	631V	632E	651Y	652K
Bobcat	628F	629H	630A	631V	632E	651Y	652K
Canada lynx	628F	629H	630A	631V	632E	651Y	652K
Fishing cat	628F	629H	630A	631V	632E	651Y	652K
Malayan pangolin	628F	629H	630A	631V	632E	651Y	652K
Chinese tree shrew	628F	629H	630A	631V	632E	651Y	652K
American mink	628F	629H	630A	631V	632E	651Y	652K
Domestic ferret	628F	629H	630A	631V	632E	651Y	652K
Eurasian badger	628F	629H	630A	631V	632E	651Y	652K
Dog	628F	629H	630A	631V	632E	651Y	652K
Red fox	628F	629H	630A	631V	632E	651Y	652K
Egyptian rousette	628F	629H	630A	631V	632E	651Y	652K
Pig	628F	629H	630A	631V	632E	651Y	652K
Gray squirrel	628F	629H	630A	631V	632E	651Y	652K
Cattle	628F	629H	630A	631V	632E	651Y	652K
White-tailed deer	628F	629H	630A	631V	632E	651Y	652K
Golden hamster	628F	629H	630A	631V	632E	651Y	652K
Norway rat	628F	629H	630A	631V	632E	651Y	652K
House mouse	628F	629H	630A	631V	632E	651Y	652K
Prairie deer mouse	628F	629H	630A	631V	632E	651Y	652K
Raccoon dog	655F	656H	657A	658V	659E	678Y	679K
Chinese alligator	628F	629H	630S	631V	632E	651Y	652K
Rock pigeon	630F	631H	632S	633V	634E	653Y	654K
African clawed frog	627F	628H	629S	630V	631E	650Y	651K
Fathead minnow	625I	626R	627S	628V	629E	648Y	649R

Total Match
Partial Match
Not a Match

Common Name	Amino Acid 1	Amino Acid 2	Amino Acid 3	Amino Acid 4	Amino Acid 5	Amino Acid 6	Amino Acid 7	Amino Acid 8
Human	584K	585E	588R	616W	627D	633P	634Y	653V
Big brown bat	584K	585E	588R	616W	627Y	633P	634Y	653V
Western lowland gorilla	584K	585E	588R	616W	627D	633P	634Y	653V
Rhesus monkey	584K	585E	588R	616W	627Y	633P	634Y	653V
Green monkey	584K	585E	588R	616W	627Y	633P	634Y	653V
Olive baboon	584K	585E	588R	616W	627Y	633P	634Y	653V
White-tufted-ear marmoset	584K	585E	588R	616W	627Y	633P	634Y	653V
Domestic cat	584K	585E	588R	616W	627Y	633P	634Y	653V
Lion	584K	585E	588R	616W	627Y	633P	634Y	653V
Tiger	584K	585E	588R	616W	627Y	633P	634Y	653V
Puma	584K	585E	588R	616W	627Y	633P	634Y	653V
Bobcat	584K	585E	588R	616W	627Y	633P	634Y	653V
Canada lynx	584K	585E	588R	616W	627Y	633P	634Y	653V
Fishing cat	584K	585E	588R	616W	627Y	633P	634Y	653V
Malayan pangolin	584K	585E	588R	616W	627Y	633P	634Y	653V
Chinese tree shrew	584K	585E	588R	616W	627Y	633P	634Y	653V
American mink	584K	585E	588R	616W	627H	633P	634Y	653V
Domestic ferret	584K	585E	588R	616W	627H	633P	634Y	653V
Eurasian badger	584K	585E	588R	616W	627H	633P	634Y	653V
Dog	584K	585E	588R	616W	627Y	633P	634Y	653V
Red fox	584K	585E	588R	616W	627Y	633P	634Y	653V
Egyptian rousette	584K	585E	588R	616W	627Y	633P	634Y	653V
Pig	584K	585E	588R	616W	627Y	633P	634Y	653V
Gray squirrel	584K	585E	588R	616W	627Y	633P	634Y	653V
Cattle	584K	585E	588R	616W	627Y	633P	634Y	653V
White-tailed deer	584K	585E	588R	616W	627Y	633P	634Y	653V
Golden hamster	584K	585E	588R	616W	627D	633P	634Y	653V
Norway rat	584K	585E	588R	616W	627D	633P	634Y	653V
House mouse	584K	585E	588R	616W	627D	633P	634Y	653V
Prairie deer mouse	584K	585E	588R	616W	627D	633P	634Y	653V
Raccoon dog	611K	612E	615R	643W	654Y	660P	661Y	680V
Chinese alligator	586K	587E	590R	618W	627Q	633P	634Y	653V
Rock pigeon	588K	589E	592R	620W	629Q	635P	636Y	655V
African clawed frog	585K	586E	589R	617W	626L	632P	633Y	652V
Fathead minnow	582K	583D	586K	614W	624Q	630P	631Y	650V

Figure S20c. Signal transducer and activator of transcription 1 (STAT1)
Query Accession: NP_009330.1
Level 3: Phosphorylation Site

Total Match
Partial Match
Not a Match

Common Name	Amino Acid 1	Amino Acid 2	Amino Acid 3	Amino Acid 4	Amino Acid 5	Amino Acid 6	Amino Acid 7	Amino Acid 8	Amino Acid 9
Human	701Y	702I	703K	704T	705E	706L	707I	724L	727S
Big brown bat	701Y	702I	703K	704T	705E	706L	707I	724L	727S
Western lowland gorilla	701Y	702I	703K	704T	705E	706L	707I	724L	727S
Rhesus monkey	701Y	702I	703K	704T	705E	706L	707I	724L	727S
Green monkey	701Y	702I	703K	704T	705E	706L	707I	724L	727S
Olive baboon	701Y	702I	703K	704T	705E	706L	707I	724L	727S
White-tufted-ear marmoset	701Y	702I	703K	704T	705E	706L	707I	724L	727S
Domestic cat	701Y	702I	703K	704T	705E	706L	707I	724L	727S
Lion	701Y	702I	703K	704T	705E	706L	707I	724L	727S
Tiger	701Y	702I	703K	704T	705E	706L	707I	724L	727S
Puma	701Y	702I	703K	704T	705E	706L	707I	724L	727S
Bobcat	701Y	702I	703K	704T	705E	706L	707I	724L	727S
Canada lynx	701Y	702I	703K	704T	705E	706L	707I	724L	727S
Fishing cat	701Y	702I	703K	704T	705E	706L	707I	724L	727S
Malayan pangolin	701Y	702I	703K	704T	705E	706L	707I	724L	727S
Chinese tree shrew	701Y	702I	703K	704T	705E	706L	707I	724L	727S
American mink	701Y	702I	703K	704T	705E	706L	707I	724L	727S
Domestic ferret	701Y	702I	703K	704T	705E	706L	707I	724L	727S
Eurasian badger	701Y	702I	703K	704T	705E	706L	707I	724L	727S
Dog	701Y	702I	703K	704T	705E	706L	707I	724L	727S
Red fox	701Y	702I	703K	704T	705E	706L	707I	724L	727S
Egyptian rousette	701Y	702I	703K	704T	705E	706L	707I	724L	727S
Pig	701Y	702I	703K	704T	705E	706L	707I	724L	727S
Gray squirrel	701Y	702I	703K	704T	705E	706L	707I	724L	727S
Cattle	701Y	702I	703K	704T	705E	706L	707I	724L	727S
White-tailed deer	701Y	702I	703K	704T	705E	706L	707I	724L	727S
Golden hamster	701Y	702I	703K	704T	705E	706L	707I	724L	727S
Norway rat	701Y	702I	703K	704T	705E	706L	707I	724L	727S
House mouse	701Y	702I	703K	704T	705E	706L	707I	724L	727S
Prairie deer mouse	701Y	702I	703K	704T	705E	706L	707I	724L	727S
Raccoon dog	728Y	729I	730K	731T	732E	733L	734I	803L	806H
Chinese alligator	701Y	702I	703K	704T	705E	706L	707I	724L	727S
Rock pigeon	703Y	704I	705K	706T	707E	708L	709I	726L	729S
African clawed frog	700Y	701I	702K	703T	704E	705L	706I	723L	726S
Fathead minnow	698Y	699I	700S	701T	702T	703L	704I	719M	722S

Figure S21a. Signal transducer and activator of transcription 2 (STAT2)
Query Accession: NP_005410.1
Level 1

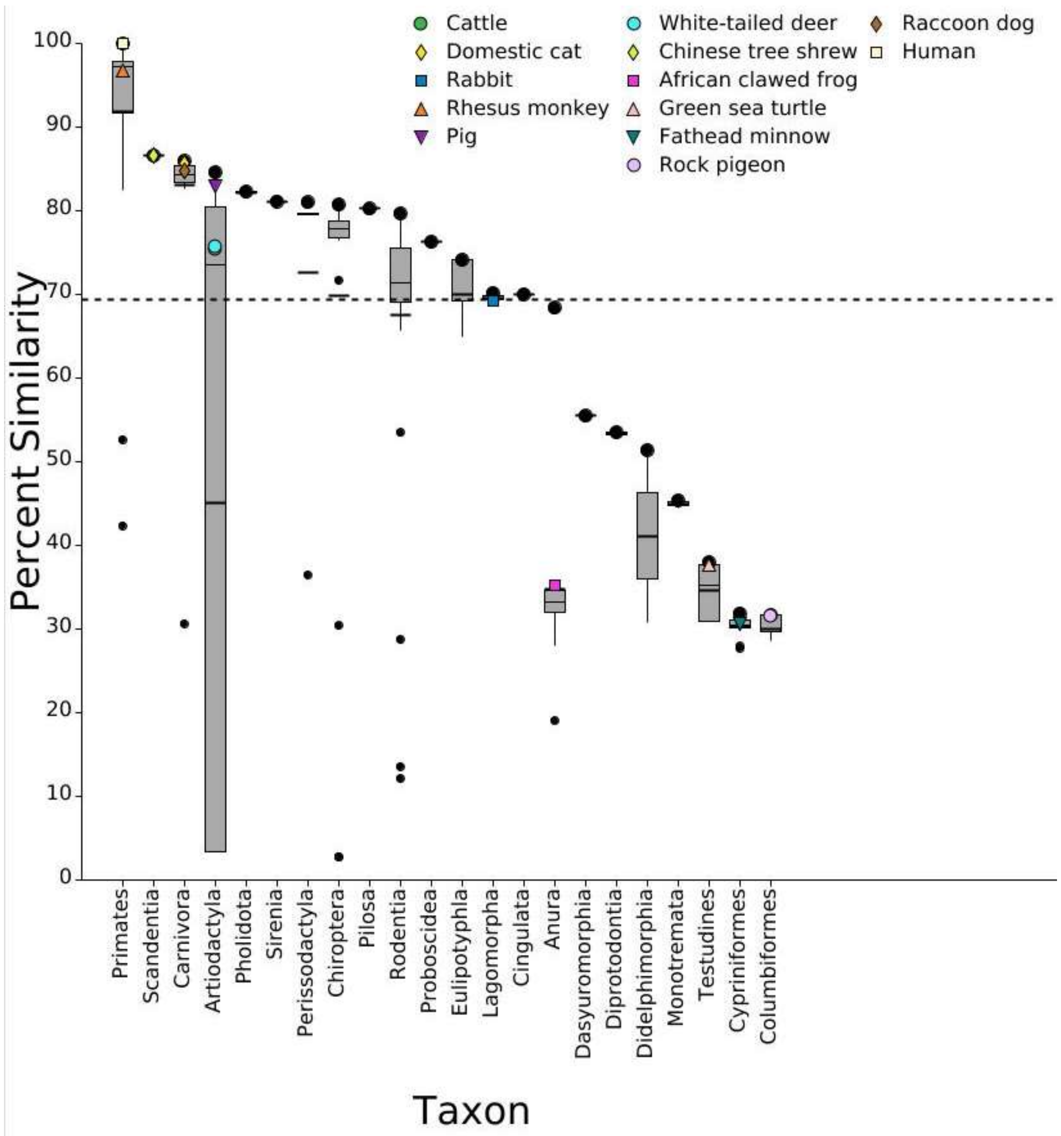

Figure S21b. Signal transducer and activator of transcription 2 (STAT2)
Query Accession: NP_005410.1 Level 2: cd10373 SH2_STAT2

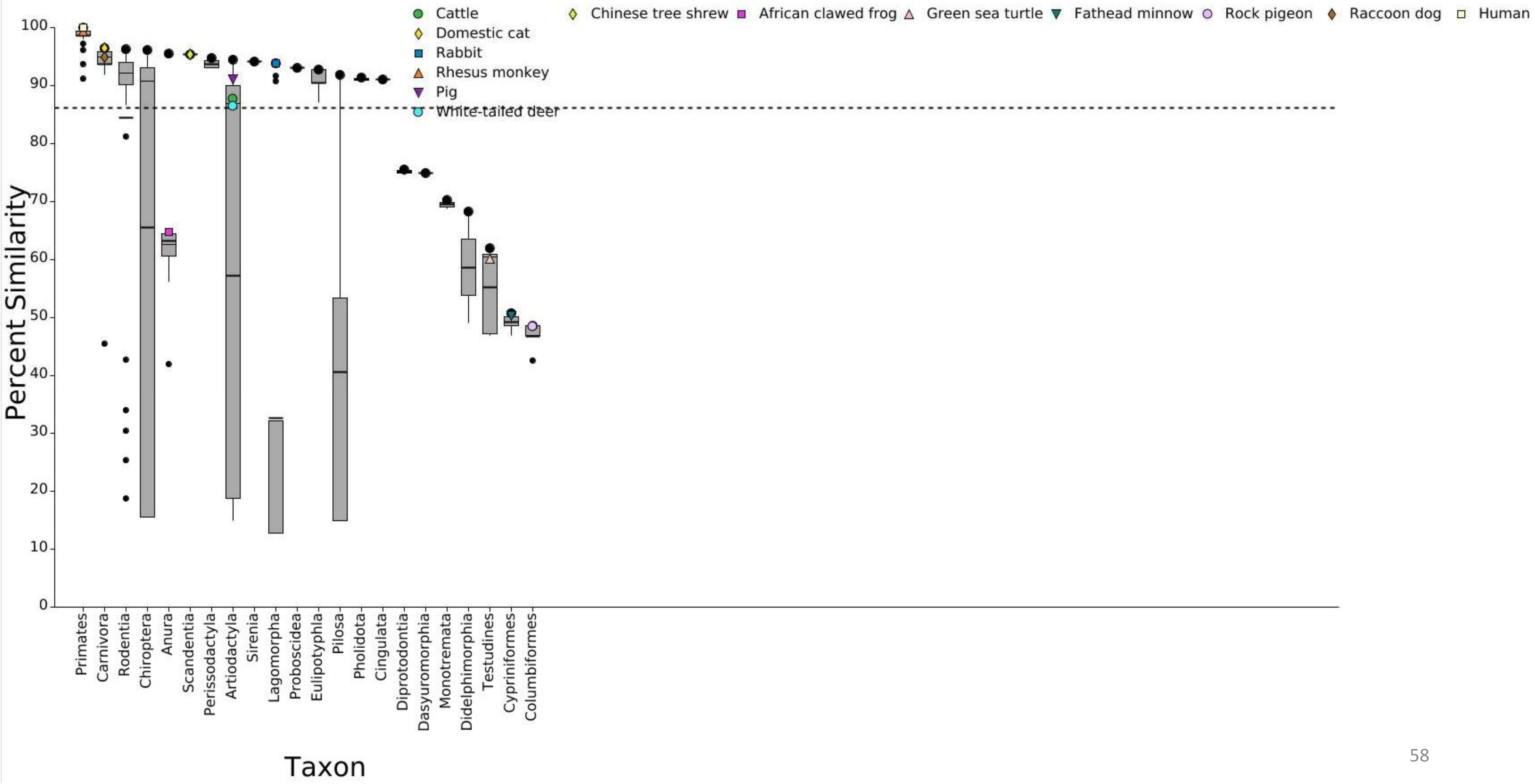

Figure S21c. Signal transducer and activator of transcription 2 (STAT2)
Query Accession: NP_005410.1
Level 3: Phosphorylation Site

(dark blue)	Total Match
(light blue)	Partial Match
(yellow)	Not a Match

Common Name	Amino Acid 1	Amino Acid 2	Amino Acid 3	Amino Acid 4	Amino Acid 5
Human	583R	601R	627S	629Q	690Y
Western lowland gorilla	583R	601R	627S	629Q	690Y
Green monkey	583R	601R	627S	629Q	690Y
Rhesus monkey	583R	601R	627S	629Q	690Y
White-tufted-ear marmoset	583R	601R	627S	629Q	690Y
Chinese tree shrew	583R	601R	627S	629Q	690Y
Fishing cat	583R	601R	627S	629Q	690Y
Puma	583R	601R	627S	629Q	690Y
Domestic cat	583R	601R	627S	629Q	690Y
Lion	583R	601R	627S	629Q	690Y
Bobcat	583R	601R	627S	629Q	690Y
Tiger	583R	601R	627S	629Q	690Y
Canada lynx	583R	601R	627S	629Q	690Y
Red fox	582R	600R	626S	628Q	689Y
Raccoon dog	582R	600R	626S	628Q	689Y
Dog	582R	600R	626S	628Q	689Y
American mink	583R	601R	627S	629Q	690Y
Eurasian badger	583R	601R	627S	629Q	690Y
Domestic ferret	583R	601R	627S	629Q	690Y
Pig	583R	601R	627S	629Q	690Y
Malayan pangolin	583R	601R	627S	629Q	690Y
Egyptian rousette	588R	606R	632S	634Q	695Y
Gray squirrel	582R	600R	626S	628Q	689Y
White-tailed deer	583R	601R	627S	629Q	690Y
Cattle	583R	601R	627S	629Q	690Y
Golden hamster	583R	601R	627S	629Q	690Y
Norway rat	583R	601R	627S	629Q	690Y
Prairie deer mouse	582R	600R	626S	628Q	689Y
House mouse	582R	600R	626S	628Q	689Y
African clawed frog	582R	600R	626S	628Q	690Y
Rock pigeon	588K	606R	632S	634E	703Y
Fathead minnow	580K	598R	625T	627K	689Y

Figure S22a. Stimulator of interferon genes (STING) Query Accession: NP_938023.1 Level 1

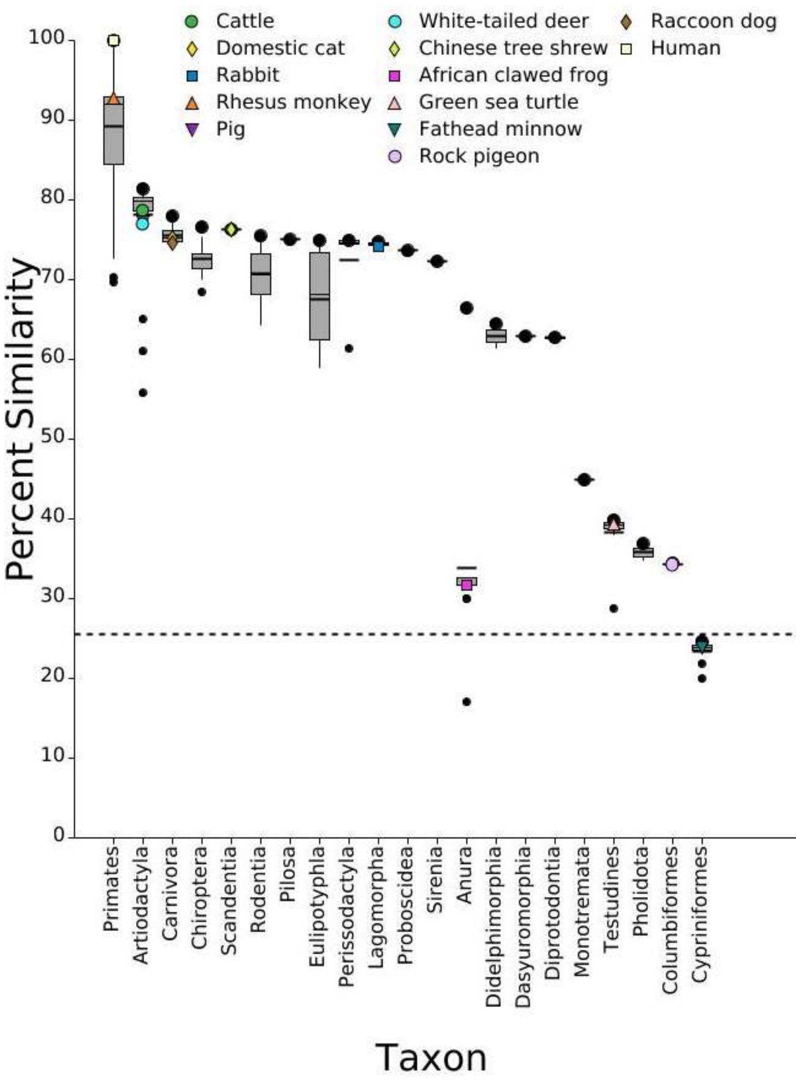

Figure S22b. STING Query Accession: NP_938023.1 Level 2: cd12146 STING_C (C-terminal binding domain of STING)

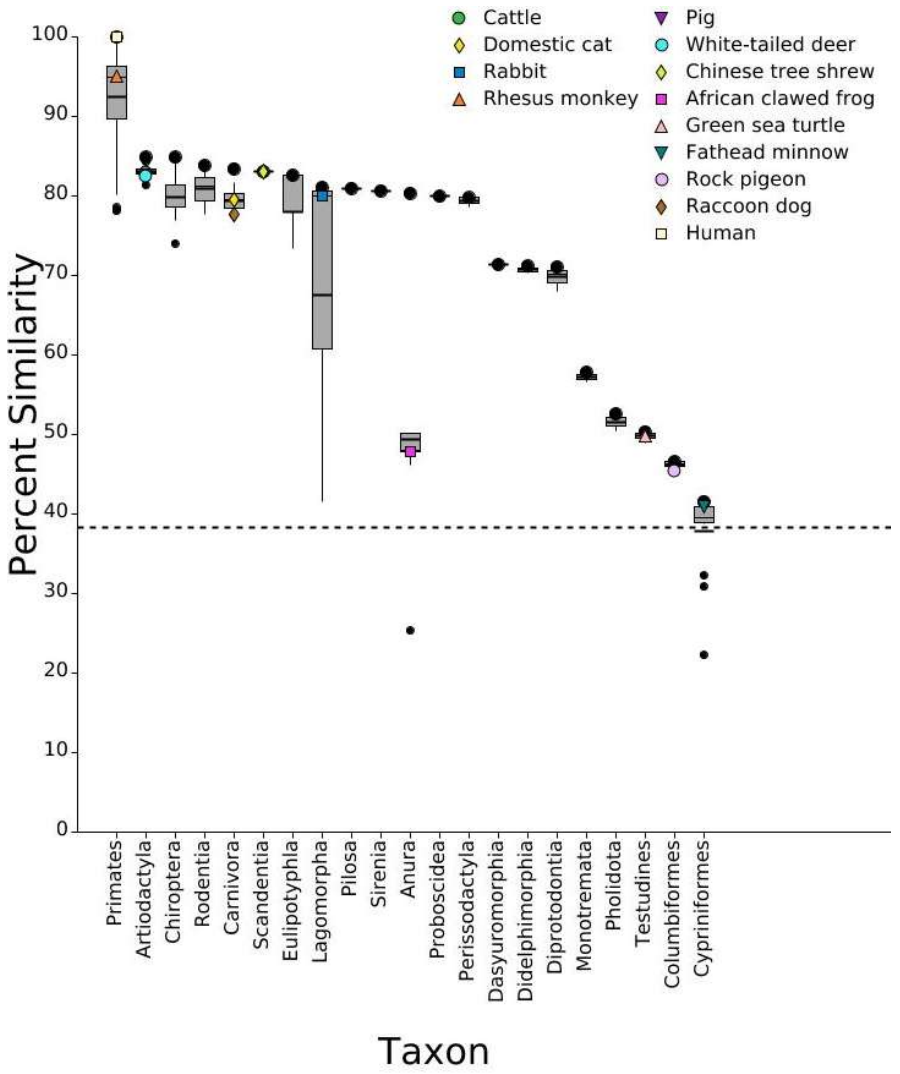

Figure S23a. TANK-binding kinase 1 (TBK1) Query Accession: NP_037386.1
Level 1

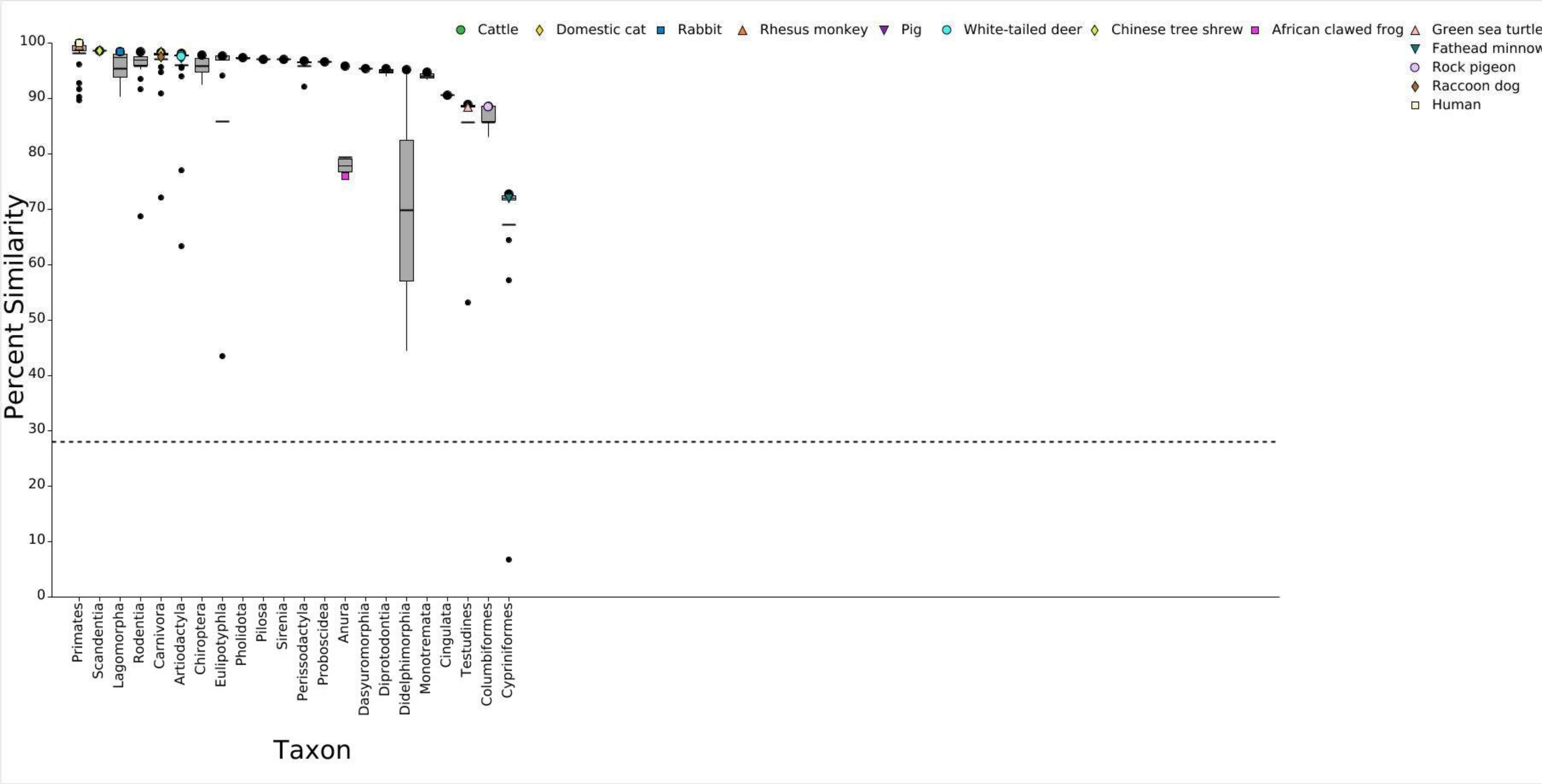

Figure S23b. TANK-binding kinase 1 (TBK1) Query Accession: NP_037386.1
Level 2: cd13988 STKc_TBK1 (Catalytic domain of the Serine/Threonine kinase, TBK1 for transfer of the gamma-phosphoryl group from ATP to serine/threonine residues on protein substrates.)

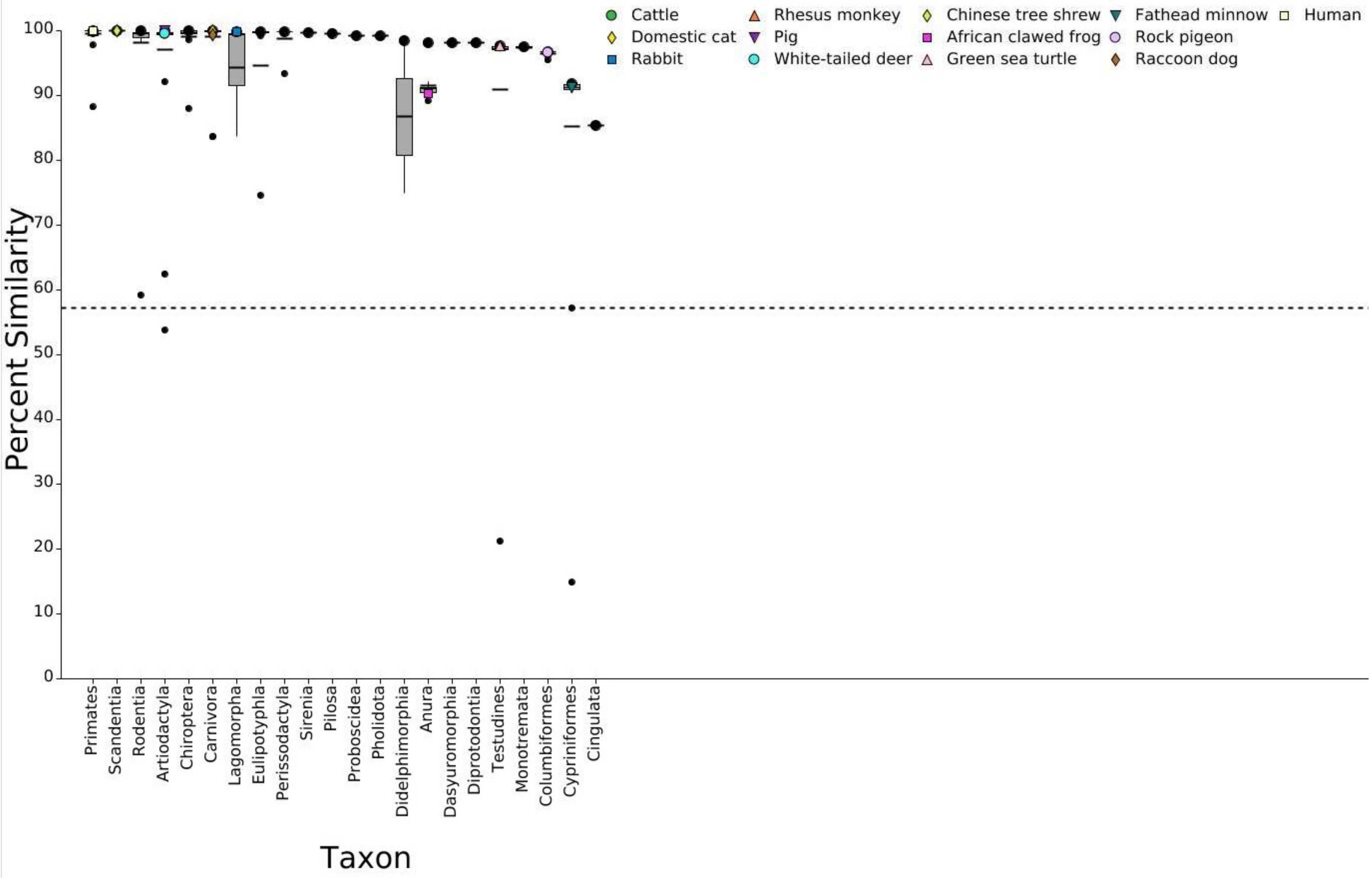

Figure S23c. TANK-binding kinase 1 (TBK1) Query Accession: NP_037386.1 Level 3: All Contacts part 1

- Total Match
- Partial Match
- Not a Match

Common Name	Amino Acid 1	Amino Acid 2	Amino Acid 3	Amino Acid 4	Amino Acid 5	Amino Acid 6	Amino Acid 7	Amino Acid 8	Amino Acid 9	Amino Acid 10	Amino Acid 11	Amino Acid 12	Amino Acid 13	Amino Acid 14	Amino Acid 15	Amino Acid 16
Human	50D	51V	52Q	54R	123M	127R	132V	133H	134R	135D	159G	177E	180L	181H	182P	183D
Western lowland gorilla	50D	51V	52Q	54R	123M	127R	132V	133H	134R	135D	159G	177E	180L	181H	182P	183D
Green monkey	50D	51V	52Q	54R	123M	127R	132V	133H	134R	135D	159G	177E	180L	181H	182P	183D
Rhesus monkey	50D	51V	52Q	54R	123M	127R	132V	133H	134R	135D	159G	177E	180L	181H	182P	183D
White-tufted-ear marmoset	50D	51V	52Q	54R	123M	127R	132V	133H	134R	135D	159G	177E	180L	181H	182P	183D
Chinese tree shrew	50D	51V	52Q	54R	123M	127R	132V	133H	134R	135D	159G	177E	180L	181H	182P	183D
Bobcat	50D	51V	52Q	54R	123M	127R	132V	133H	134R	135D	159G	177E	180L	181H	182P	183D
Domestic cat	50D	51V	52Q	54R	123M	127R	132V	133H	134R	135D	159G	177E	180L	181H	182P	183D
Lion	50D	51V	52Q	54R	123M	127R	132V	133H	134R	135D	159G	177E	180L	181H	182P	183D
Tiger	50D	51V	52Q	54R	123M	127R	132V	133H	134R	135D	159G	177E	180L	181H	182P	183D
Fishing cat	50D	51V	52Q	54R	123M	127R	132V	133H	134R	135D	159G	177E	180L	181H	182P	183D
American mink	50D	51V	52Q	54R	123M	127R	132V	133H	134R	135D	159G	177E	180L	181H	182P	183D
Domestic ferret	50D	51V	52Q	54R	123M	127R	132V	133H	134R	135D	159G	177E	180L	181H	182P	183D
Eurasian badger	50D	51V	52Q	54R	123M	127R	132V	133H	134R	135D	159G	177E	180L	181H	182P	183D
Cattle	50D	51V	52Q	54R	123M	127R	132V	133H	134R	135D	159G	177E	180L	181H	182P	183D
White-tailed deer	50D	51V	52Q	54R	123M	127R	132V	133H	134R	135D	159G	177E	180L	181H	182P	183D
Raccoon dog	50D	51V	52Q	54R	123M	127R	132V	133H	134R	135D	159G	177E	180L	181H	182P	183D
Red fox	93D	94V	95Q	97R	166M	170R	175V	176H	177R	178D	202G	220E	223L	224H	225P	226D
Dog	50D	51V	52Q	54R	123M	127R	132V	133H	134R	135D	159G	177E	180L	181H	182P	183D
Prairie deer mouse	50D	51V	52Q	54R	123M	127R	132V	133H	134R	135D	159G	177E	180L	181H	182P	183D
Malayan pangolin	50D	51V	52Q	54R	123M	127R	132V	133H	134R	135D	159G	177E	180L	181H	182P	183D
Pig	50D	51V	52Q	54R	123M	127R	132V	133H	134R	135D	159G	177E	180L	181H	182P	183D
Egyptian rousette	50D	51V	52Q	54R	123M	127R	132V	133H	134R	135D	159G	177E	180L	181H	182P	183D
House mouse	50D	51V	52Q	54R	123M	127R	132V	133H	134R	135D	159G	177E	180L	181H	182P	183D
Norway rat	50D	51V	52Q	54R	123M	127R	132V	133H	134R	135D	159G	177E	180L	181H	182P	183D
Puma	50L	51I	52Q	54L	128M	132R	137V	138H	139R	140D	164G	182E	185L	186H	187P	188D
Big brown bat	50D	51V	52Q	54R	123M	127R	132V	133H	134R	135D	159G	177E	180L	181H	182P	183D
Rock pigeon	50D	51V	52Q	54R	123M	127R	132V	133H	134R	135D	159G	177E	180L	181H	182P	183D
Chinese alligator	50E	51V	52Q	54R	123M	127R	132V	133H	134R	135D	159G	177E	180L	181H	182P	183D
African clawed frog	50D	51V	52Q	54R	123M	127R	132I	133H	134R	135D	159G	177E	180L	181H	182P	183D
Fathead minnow	50D	51V	52Q	54R	123M	127R	132V	133H	134R	135D	159G	177E	180L	181H	182P	183D

Figure S23c. TANK-binding kinase 1 (TBK1) Query Accession: NP_037386.1 Level 3: All Contacts part 2

Total Match
Partial Match
Not a Match

Common Name	Amino Acid 1	Amino Acid 2	Amino Acid 3	Amino Acid 4	Amino Acid 5	Amino Acid 6	Amino Acid 7	Amino Acid 8	Amino Acid 9	Amino Acid 10	Amino Acid 11	Amino Acid 12	Amino Acid 13
Human	200A	201T	202V	203D	204L	206S	207I	232E	235Y	289Q	290E	294G	295F
Western lowland gorilla	200A	201T	202V	203D	204L	206S	207I	232E	235Y	289Q	290E	294G	295F
Green monkey	200A	201T	202V	203D	204L	206S	207I	232E	235Y	289Q	290E	294G	295F
Rhesus monkey	200A	201T	202V	203D	204L	206S	207I	232E	235Y	289Q	290E	294G	295F
White-tufted-ear marmoset	200A	201T	202V	203D	204L	206S	207I	232E	235Y	289Q	290E	294G	295F
Chinese tree shrew	200A	201T	202V	203D	204L	206S	207I	232E	235Y	289Q	290E	294G	295F
Bobcat	200A	201T	202V	203D	204L	206S	207I	232E	235Y	289Q	290E	294G	295F
Domestic cat	200A	201T	202V	203D	204L	206S	207I	232E	235Y	289Q	290E	294G	295F
Lion	200A	201T	202V	203D	204L	206S	207I	232E	235Y	289Q	290E	294G	295F
Tiger	200A	201T	202V	203D	204L	206S	207I	232E	235Y	289Q	290E	294G	295F
Fishing cat	200A	201T	202V	203D	204L	206S	207I	232E	235Y	289Q	290E	294G	295F
American mink	200A	201T	202V	203D	204L	206S	207I	232E	235Y	289Q	290E	294G	295F
Domestic ferret	200A	201T	202V	203D	204L	206S	207I	232E	235Y	289Q	290E	294G	295F
Eurasian badger	200A	201T	202V	203D	204L	206S	207I	232E	235Y	289Q	290E	294G	295F
Cattle	200A	201T	202V	203D	204L	206S	207I	232E	235Y	289Q	290E	294G	295F
White-tailed deer	200A	201T	202V	203D	204L	206S	207I	232E	235Y	289Q	290E	294G	295F
Raccoon dog	200A	201T	202V	203D	204L	206S	207I	232E	235Y	289Q	290E	294G	295F
Red fox	243A	244T	245V	246D	247L	249S	250I	275E	278Y	332Q	333E	337G	338F
Dog	200A	201T	202V	203D	204L	206S	207I	232E	235Y	289Q	290E	294G	295F
Prairie deer mouse	200A	201T	202V	203D	204L	206S	207I	232E	235Y	289Q	290E	294G	295F
Malayan pangolin	200A	201T	202V	203D	204L	206S	207I	232E	235Y	289Q	290E	294G	295F
Pig	200A	201T	202V	203D	204L	206S	207I	232E	235Y	289Q	290E	294G	295F
Egyptian rousette	200A	201T	202V	203D	204L	206S	207I	232E	235Y	289Q	290E	294G	295F
House mouse	200A	201T	202V	203D	204L	206S	207V	232E	235Y	289Q	290E	294G	295F
Norway rat	200A	201T	202V	203D	204L	206S	207V	232E	235Y	289Q	290E	294G	295F
Puma	205A	206T	207V	208D	209L	211S	212I	237E	240Y	294Q	295E	299G	300F
Big brown bat	200A	201T	202V	203D	204L	206S	207I	232E	235Y	289Q	290E	294G	295F
Rock pigeon	200A	201T	202V	203D	204L	206S	207I	232E	235Y	289Q	290E	294G	295F
Chinese alligator	200A	201T	202V	203D	204L	206S	207I	232E	235Y	289Q	290E	294G	295F
African clawed frog	200A	201T	202V	203D	204L	206S	207I	232E	235Y	289Q	290E	294G	295F
Fathead minnow	200A	201T	202V	203D	204L	206S	207I	232E	235Y	289Q	290E	294G	295F

Figure S24a. Transmembrane serine protease 2 (TMPRSS2) Query Accession:NP_005647.3 Level 1

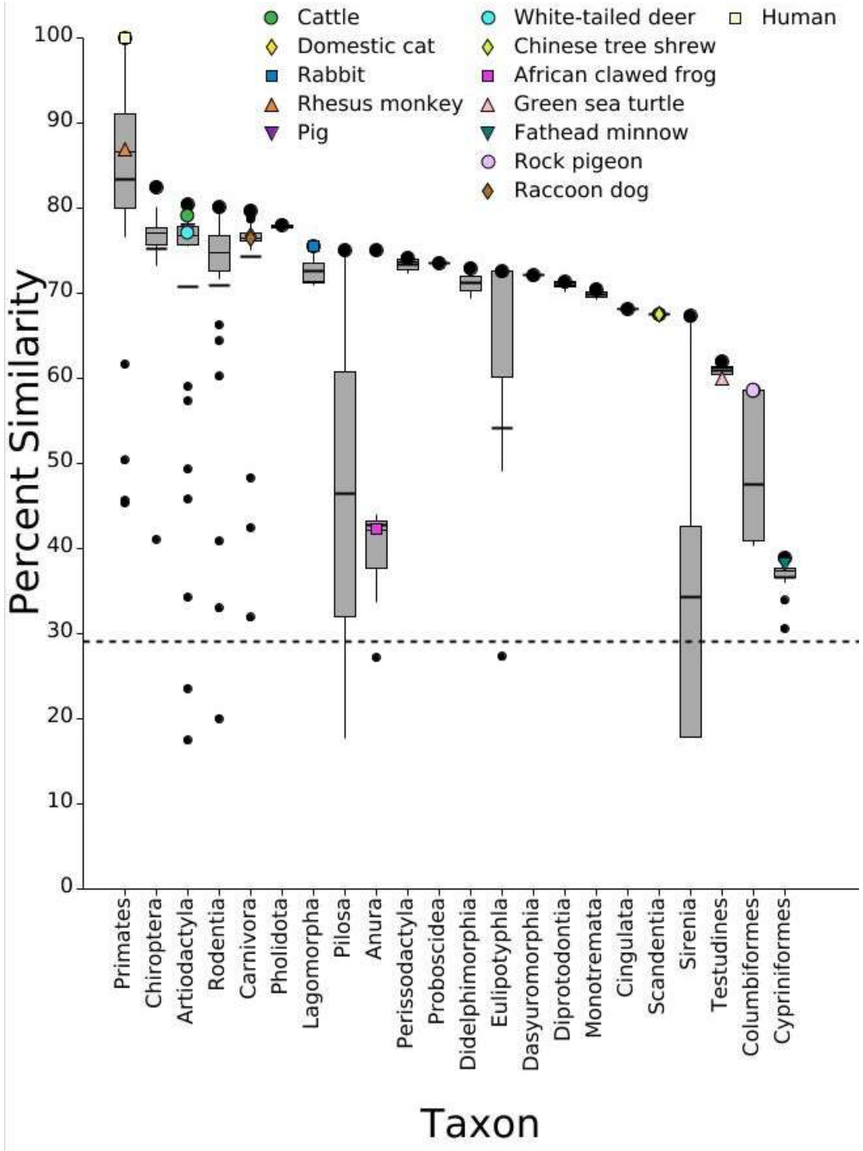

Figure S24b. Transmembrane serine protease 2 (TMPRSS2)
Query Accession NP_005647.3
Level 2: cd00190 Tryp_SPc (Trypsin-like serine protease active/cleavage site)

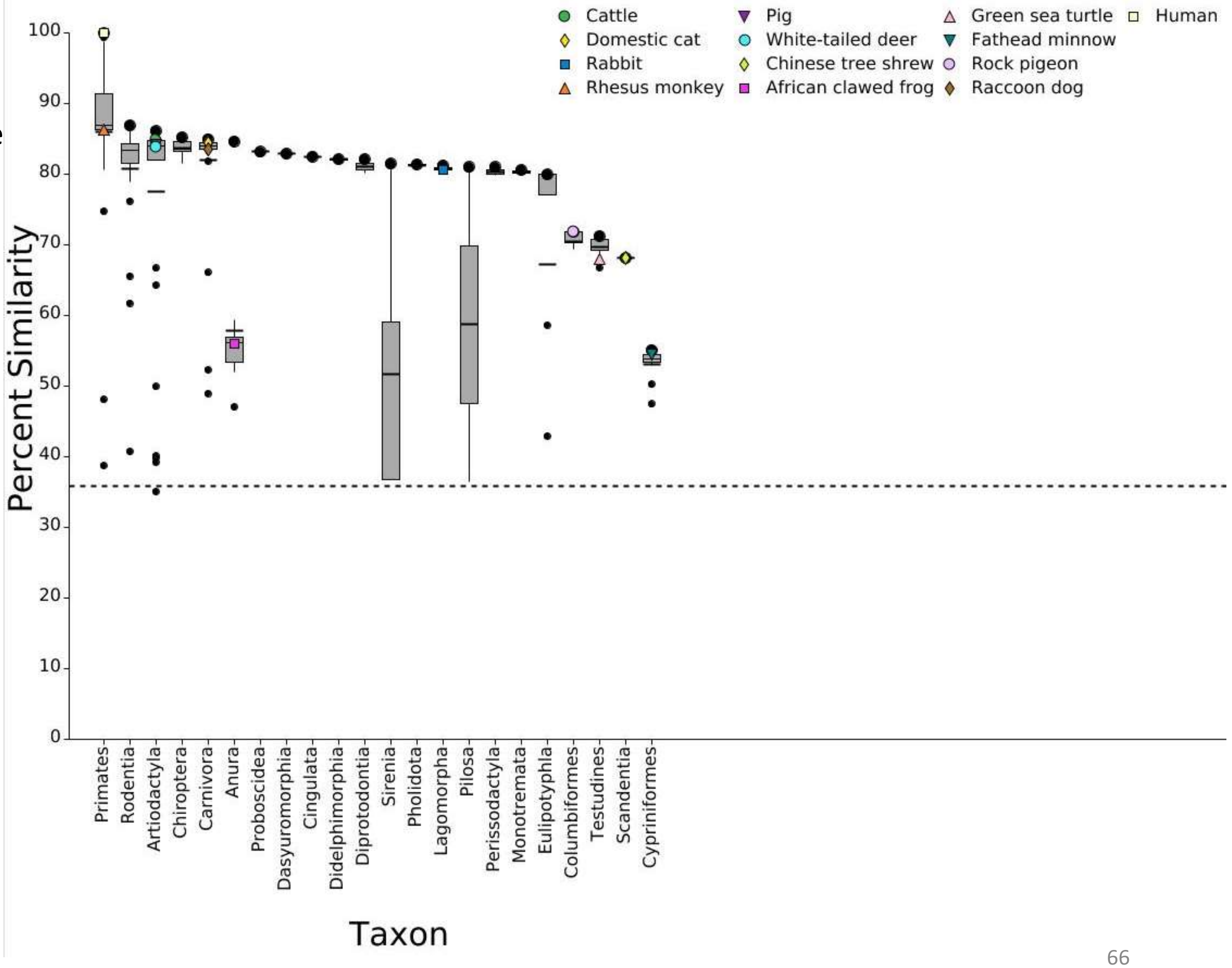

Figure S24c. Transmembrane serine protease 2 TMPRSS2 Query Accession: NP_005647.3 Level 3: Bond

Total Match
Partial Match
Not a Match

Common Name	Amino Acid 1	Amino Acid 2	Amino Acid 3	Amino Acid 4	Amino Acid 5	Amino Acid 6	Amino Acid 7	Amino Acid 8	Amino Acid 9	Amino Acid 10	Amino Acid 11	Amino Acid 12	Amino Acid 13	Amino Acid 14	Amino Acid 15	Amino Acid 16
Human	296H	299E	338D	340K	341T	342K	389E	391G	392K	436S	438Q	439G	441S	460S	462G	464G
Big brown bat	296H	299E	338D	340K	341T	342K	389E	391G	392K	436S	438Q	439G	441S	460S	462G	464G
Western lowland gorilla	296H	299E	338D	340K	341T	342K	389E	391G	392K	436S	438Q	439G	441S	460S	462G	464G
Olive baboon	329H	332E	371D	373K	374T	375K	422E	424G	425K	469S	471Q	472G	474S	493S	495G	497G
White-tufted-ear marmoset	296H	299E	338D	340Q	341T	342K	389E	391G	392K	436S	438Q	439G	441S	460S	462G	464G
Rhesus monkey	338H	341E	380D	382K	383T	384K	431E	433G	434K	478S	480Q	481G	483S	502S	504G	506G
Green monkey	465H	468E	507D	509K	510T	511K	558E	560G	561K	605S	607Q	608G	610S	629S	631G	633G
Gray squirrel	295H	298E	337D	339K	340T	341K	388E	390G	391K	435S	437Q	438G	440S	459S	461G	463G
American mink	296H	299E	338D	340K	341T	342K	389E	391G	392K	436S	438Q	439G	441S	460S	462G	464G
Cattle	294H	297E	336D	338K	339T	340K	387E	389G	390K	434S	436Q	437G	439S	458S	460G	462G
Domestic ferret	296H	299E	338D	340K	341T	342K	389E	391G	392K	436S	438Q	439G	441S	460S	462G	464G
Eurasian badger	296H	299E	338D	340K	341T	342K	389E	391G	392K	436S	438Q	439G	441S	460S	462G	464G
House mouse	294H	297E	336D	338K	339T	340K	387E	389G	390K	434S	436Q	437G	439S	458S	460G	462G
Malayan pangolin	296H	299E	338D	340Q	341T	342K	389E	391G	392K	436S	438Q	439G	441S	460S	462G	464G
Pig	299H	302E	341D	343K	344T	345K	392E	394G	395K	439S	441Q	442G	444S	463S	465G	467G
White-tailed deer	294H	297E	336D	338K	339T	340K	387E	389G	390K	434S	436Q	437G	439S	458S	460G	462G
Dog	296H	299E	338D	340K	341T	342K	389E	391G	392K	436S	438Q	439G	441S	460S	462G	464G
Norway rat	294H	297E	336D	338K	339T	340K	387E	389G	390K	434S	436Q	437G	439S	458S	460G	462G
Domestic cat	296H	299E	338D	340K	341T	342K	389E	391G	392K	436S	438Q	439G	441S	460S	462G	464G
Tiger	296H	299E	338D	340K	341S	342K	389E	391G	392K	436S	438Q	439G	441S	460S	462G	464G
Raccoon dog	296H	299E	338D	340K	341T	342K	389E	391G	392K	436S	438Q	439G	441S	460S	462G	464G
Red fox	296H	299E	338D	340K	341T	342K	389E	391G	392K	436S	438Q	439G	441S	460S	462G	464G
Lion	296H	299E	338D	340K	341S	342K	389E	391G	392K	436S	438Q	439G	441S	460S	462G	464G
Canada lynx	296H	299E	338D	340K	341T	342K	389E	391G	392K	436S	438Q	439G	441S	460S	462G	464G
Bobcat	296H	299E	338D	340K	341T	342K	389E	391G	392K	436S	438Q	439G	441S	460S	462G	464G
Prairie deer mouse	298H	301E	340D	342K	343T	344K	391E	393G	394K	438S	440Q	441G	443S	462S	464G	466G
Egyptian rousette	299H	302E	341D	343K	344T	345K	392E	394G	395K	439S	441Q	442G	444S	463S	465G	467G
Golden hamster	295H	298E	337N	339E	340T	341K	388E	390G	391K	435S	437Q	438G	440S	459S	461G	463G
Puma	--	--	288D	290K	291T	292K	--	309G	310K	354S	356Q	357G	359S	378S	380G	382G
Chinese alligator	293H	296E	335N	337E	338T	339K	386Q	388G	389K	433S	435Q	436G	438S	457S	459G	461G
Rock pigeon	288H	291E	330D	332D	333S	334K	381Q	383G	384K	428S	430Q	431G	433S	452S	454G	456G
African clawed frog	289H	292E	329D	331K	332T	333K	380E	382G	383T	427S	429Q	430G	432S	451S	453G	455G
Fathead minnow	300H	303Q	340D	342R	343T	344N	392S	394G	395L	439S	441Q	442G	444S	463S	465G	467G

Figure S24c. Transmembrane serine protease 2 (TMPRSS2) Query Accession: NP_005647.3 Level 3: Distance

- Total Match
- Partial Match
- Not a Match

Common Name	Amino Acid 1	Amino Acid 2	Amino Acid 3	Amino Acid 4	Amino Acid 5	Amino Acid 6	Amino Acid 7	Amino Acid 8	Amino Acid 9	Amino Acid 10	Amino Acid 11	Amino Acid 12	Amino Acid 13
Human	280V	300K	419L	435D	437C	440D	459T	461W	463S	465C	470R	471P	472G
Big brown bat	280V	300E	419L	435D	437C	440D	459T	461W	463S	465C	470R	471P	472G
Western lowland gorilla	280V	300K	419L	435D	437C	440D	459T	461W	463S	465C	470R	471P	472G
Olive baboon	313V	333K	452L	468D	470C	473D	492T	494W	496S	498C	503R	504P	505G
White-tufted-ear marmoset	280V	300K	419L	435D	437C	440D	459T	461W	463S	465C	470R	471P	472G
Rhesus monkey	322V	342K	461L	477D	479C	482D	501T	503W	505S	507C	512R	513P	514G
Green monkey	449V	469K	588L	604D	606C	609D	628T	630W	632S	634C	639R	640P	641G
Gray squirrel	279I	299E	418L	434D	436C	439D	458T	460W	462S	464C	469R	470P	471G
American mink	280V	300E	419L	435D	437C	440D	459T	461W	463S	465C	470R	471P	472G
Cattle	278V	298E	417L	433D	435C	438D	457T	459W	461S	463C	468R	469P	470G
Domestic ferret	280V	300E	419L	435D	437C	440D	459T	461W	463S	465C	470R	471P	472G
Eurasian badger	280V	300E	419L	435D	437C	440D	459T	461W	463S	465C	470R	471P	472G
House mouse	278V	298E	417L	433D	435C	438D	457T	459W	461S	463C	468R	469P	470G
Malayan pangolin	280V	300E	419L	435D	437C	440D	459T	461W	463S	465C	470R	471P	472G
Pig	283I	303E	422L	438D	440C	443D	462T	464W	466S	468C	473R	474P	475G
White-tailed deer	278V	298E	417L	433D	435C	438D	457T	459W	461S	463C	468R	469P	470G
Dog	280V	300E	419L	435D	437C	440D	459T	461W	463S	465C	470R	471P	472G
Norway rat	278V	298E	417L	433D	435C	438D	457T	459W	461S	463C	468R	469P	470G
Domestic cat	280V	300E	419L	435D	437C	440D	459T	461W	463S	465C	470R	471P	472G
Tiger	280V	300E	419L	435D	437C	440D	459T	461W	463S	465C	470R	471P	472G
Raccoon dog	280V	300E	419L	435D	437C	440D	459T	461W	463S	465C	470R	471P	472G
Red fox	280V	300E	419L	435D	437C	440D	459T	461W	463S	465C	470R	471P	472G
Lion	280V	300E	419L	435D	437C	440D	459T	461W	463S	465C	470R	471P	472G
Canada lynx	280V	300E	419L	435D	437C	440D	459T	461W	463S	465C	470R	471P	472G
Bobcat	280V	300E	419L	435D	437C	440D	459T	461W	463S	465C	470R	471P	472G
Prairie deer mouse	282V	302E	421L	437D	439C	442D	461T	463W	465S	467C	472R	473P	474G
Egyptian rousette	283V	303E	422L	438D	440C	443D	462T	464W	466S	468C	473R	474P	475G
Golden hamster	279V	299E	418L	434D	436C	439D	458T	460W	462S	464C	469R	470P	471G
Puma	241P	--	337L	353D	355C	358D	377T	379W	381S	383C	388R	389P	390G
Chinese alligator	277V	297G	416L	432D	434C	437D	456T	458W	460T	462C	467R	468P	469G
Fathead minnow	284L	304E	422Q	438D	440C	443D	462T	464W	466D	468C	473K	474P	475G
African clawed frog	273V	293G	410A	426D	428C	431D	450T	452W	454T	456C	461K	462P	463G
Rock pigeon	272V	292G	411M	427D	429C	432D	451T	453W	455T	457C	462R	463P	464G

Figure S25a. Translocase of outer mitochondrial membrane (TOMM70)
Query Accession: NP_055635.3
Level 1

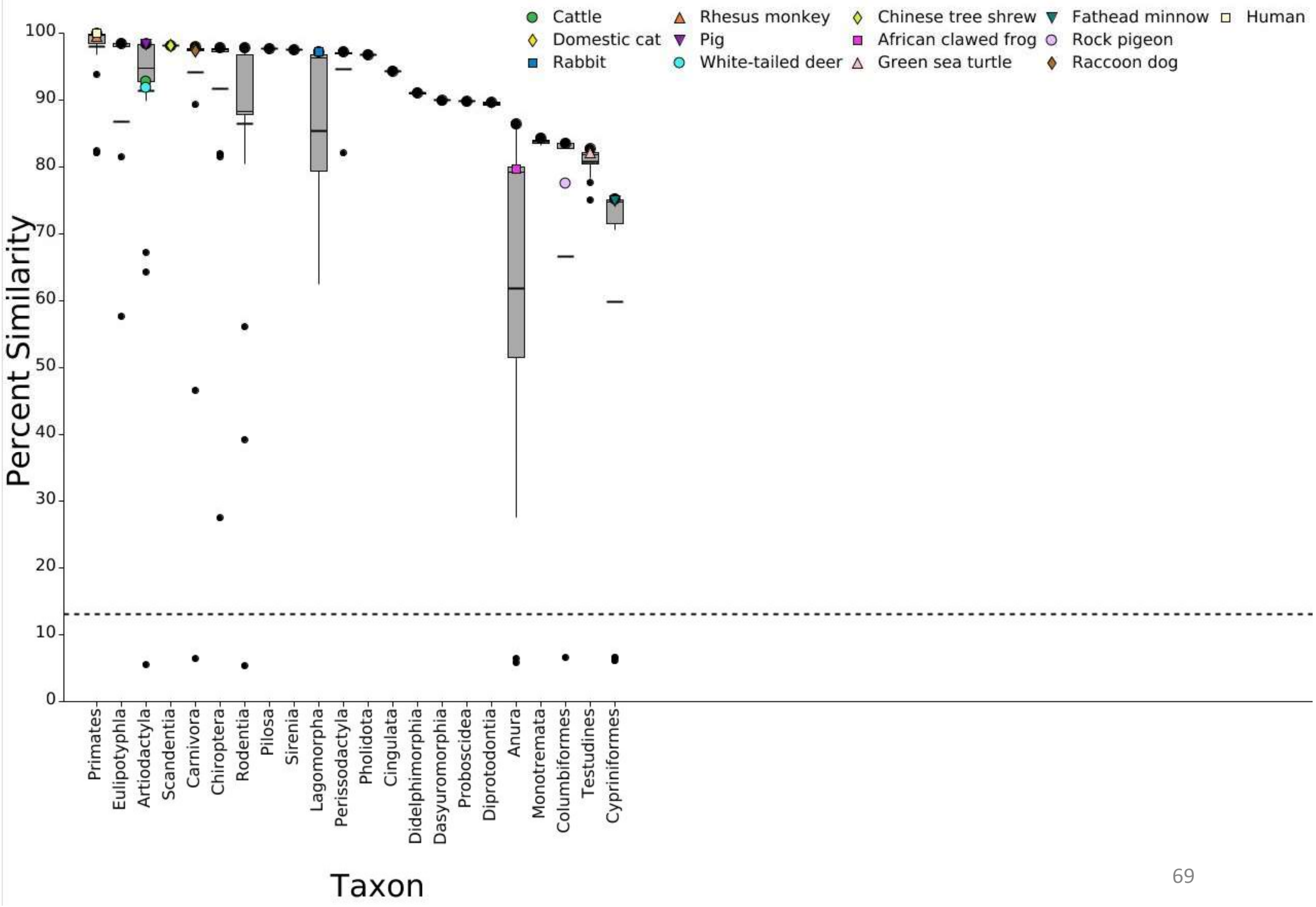

Total Match
Partial Match
Not a Match

Figure S25b. Translocase of outer mitochondrial membrane (TOMM70) Query Accession: NP_055635.3 Level 3: Bond

Common Name	Amino Acid 1	Amino Acid 2	Amino Acid 3	Amino Acid 4	Amino Acid 5	Amino Acid 6	Amino Acid 7	Amino Acid 8	Amino Acid 9	Amino Acid 10	Amino Acid 11	Amino Acid 12	Amino Acid 13
Human	219F	225M	379Q	381Q	413L	447R	477E	484Q	544C	545D	556V	580E	594Q
Western lowland gorilla	219F	225M	379Q	381Q	413L	447R	477E	484Q	544C	545D	556V	580E	594Q
Green monkey	219F	225M	379Q	381Q	413L	447R	477E	484Q	544C	545D	556V	580E	594Q
Rhesus monkey	219F	225M	379Q	381Q	413L	447R	477E	484Q	544C	545D	556V	580E	594Q
White-tufted-ear marmoset	219F	225M	379Q	381Q	413L	447R	477E	484Q	544C	545D	556V	580E	594Q
Pig	220F	226M	380Q	382Q	414L	448R	478E	485Q	545C	546D	557V	581E	595Q
Chinese tree shrew	220F	226M	380Q	382Q	414L	448R	478E	485Q	545C	546D	557V	581E	595Q
American mink	220F	226M	380Q	382Q	414L	448R	478E	485Q	545C	546D	557V	581E	595Q
Eurasian badger	220F	226M	380Q	382Q	414L	448R	478E	485Q	545C	546D	557V	581E	595Q
Egyptian rousette	220F	226M	380Q	382Q	414L	448R	478E	485Q	545C	546D	557V	581E	595Q
Domestic cat	220F	226M	380Q	382Q	414L	448R	478E	485Q	545C	546D	557V	581E	595Q
Lion	220F	226M	380Q	382Q	414L	448R	478E	485Q	545C	546D	557V	581E	595Q
Tiger	220F	226M	380Q	382Q	414L	448R	478E	485Q	545C	546D	557V	581E	595Q
Puma	220F	226M	380Q	382Q	414L	448R	478E	485Q	545C	546D	557V	581E	595Q
Canada lynx	220F	226M	380Q	382Q	414L	448R	478E	485Q	545C	546D	557V	581E	595Q
Fishing cat	220F	226M	380Q	382Q	414L	448R	478E	485Q	545C	546D	557V	581E	595Q
Domestic ferret	220F	226M	380Q	382Q	414L	448R	478E	485Q	545C	546D	557V	581E	595Q
Bobcat	220F	226M	380Q	382Q	414L	448R	478E	485Q	545C	546D	557V	581E	595Q
Raccoon dog	220F	226M	380Q	382Q	414L	448R	478E	485Q	545C	546D	557V	581E	595Q
Dog	220F	226M	380Q	382Q	414L	448R	478E	485Q	545C	546D	557V	581E	595Q
Big brown bat	222F	228M	382Q	384Q	416L	450R	480E	487Q	547C	548D	559V	583E	597Q
Gray squirrel	220F	226M	380Q	382Q	414L	448R	478E	485Q	545C	546D	557V	581E	595Q
Malayan pangolin	221F	227M	381Q	383Q	415L	449R	479E	486Q	546C	547D	558V	582E	596Q
Olive baboon	219F	225M	379Q	381Q	413L	447R	477E	484Q	544C	545D	556V	580E	594P
Cattle	220F	226M	380Q	382Q	414L	448R	478E	485Q	545C	546D	557V	581E	595Q
White-tailed deer	220F	226M	380Q	382Q	414L	448R	478E	485Q	545C	546D	557V	581E	595Q
Prairie deer mouse	223F	229M	383Q	385Q	417L	451R	481E	488Q	548C	549D	560V	584E	598Q
Norway rat	221F	227M	381Q	383Q	415L	449R	479E	486Q	546C	547D	558V	582E	596Q
Golden hamster	222F	228M	382Q	384Q	416L	450R	480E	487Q	547C	548D	559V	583E	597Q
African clawed frog	187F	193M	347Q	349Q	381L	415R	445E	452Q	512C	513D	524V	548E	562Q
Chinese alligator	112F	118M	272Q	274Q	306L	340R	370E	377Q	437C	438D	449V	473E	487Q
Rock pigeon	126F	132M	286Q	288Q	320L	354R	384E	391Q	451C	452D	463V	487E	501Q
Fathead minnow	190F	196M	350Q	352Q	384L	418R	448E	455Q	515C	516D	527V	551E	565Q

Figure S25b. Translocase of outer mitochondrial membrane (TOMM70)

Query Accession: NP_055635.3

Level 3: Distance

Total Match
Partial Match
Not a Match

Common Name	Amino Acid 1	Amino Acid 2	Amino Acid 3	Amino Acid 4	Amino Acid 5	Amino Acid 6	Amino Acid 7	Amino Acid 8	Amino Acid 9	Amino Acid 10	Amino Acid 11
Human	215I	256F	259S	260Y	340L	341L	375S	378M	409Q	410L	412I
Western lowland gorilla	215I	256F	259S	260Y	340L	341L	375S	378M	409Q	410L	412I
Green monkey	215I	256F	259S	260Y	340L	341L	375S	378M	409Q	410L	412I
Rhesus monkey	215I	256F	259S	260Y	340L	341L	375S	378M	409Q	410L	412I
White-tufted-ear marmoset	215I	256F	259S	260Y	340L	341L	375S	378M	409Q	410L	412I
Pig	216I	257F	260S	261Y	341L	342L	376S	379M	410Q	411L	413I
Chinese tree shrew	216I	257F	260S	261Y	341L	342L	376S	379M	410Q	411L	413I
American mink	216I	257F	260S	261Y	341L	342L	376S	379M	410Q	411L	413I
Eurasian badger	216I	257F	260S	261Y	341L	342L	376S	379M	410Q	411L	413I
Egyptian rousette	216I	257F	260S	261Y	341L	342L	376S	379M	410Q	411L	413I
Domestic cat	216I	257F	260S	261Y	341L	342L	376S	379M	410Q	411L	413I
Lion	216I	257F	260S	261Y	341L	342L	376S	379M	410Q	411L	413I
Tiger	216I	257F	260S	261Y	341L	342L	376S	379M	410Q	411L	413I
Puma	216I	257F	260S	261Y	341L	342L	376S	379M	410Q	411L	413I
Canada lynx	216I	257F	260S	261Y	341L	342L	376S	379M	410Q	411L	413I
Fishing cat	216I	257F	260S	261Y	341L	342L	376S	379M	410Q	411L	413I
Domestic ferret	216I	257F	260S	261Y	341L	342L	376S	379M	410Q	411L	413I
Bobcat	216I	257F	260S	261Y	341L	342L	376S	379M	410Q	411L	413I
Raccoon dog	216I	257F	260S	261Y	341L	342L	376S	379M	410Q	411L	413I
Dog	216I	257F	260S	261Y	341L	342L	376S	379M	410Q	411L	413I
Big brown bat	218I	259F	262S	263Y	343L	344L	378S	381M	412Q	413L	415I
Gray squirrel	216I	257F	260S	261Y	341L	342L	376S	379M	410Q	411L	413I
Malayan pangolin	217I	258F	261S	262Y	342L	343L	377S	380M	411Q	412L	414I
Olive baboon	215I	256F	259S	260Y	340L	341L	375S	378M	409Q	410L	412I
Cattle	216I	257F	260S	261Y	341L	342L	376S	379M	410Q	411L	413I
White-tailed deer	216I	257F	260S	261Y	341L	342L	376S	379M	410Q	411L	413I
Prairie deer mouse	219I	260F	263S	264Y	344L	345L	379T	382M	413Q	414L	416I
Norway rat	217I	258F	261S	262Y	342L	343L	377T	380M	411Q	412L	414I
Golden hamster	218I	259F	262S	263Y	343L	344L	378T	381M	412Q	413L	415I
African clawed frog	183I	224F	227S	228Y	308L	309L	343S	346M	377Q	378L	380I
Chinese alligator	108I	149F	152S	153Y	233L	234L	268S	271M	302Q	303L	305I
Rock pigeon	122I	163F	166S	167Y	247L	248L	282S	285M	316Q	317L	319I
Fathead minnow	186I	227F	230S	231Y	311L	312L	346S	349M	380Q	381L	383I

Common Name	Amino Acid 12	Amino Acid 13	Amino Acid 14	Amino Acid 15	Amino Acid 16	Amino Acid 17	Amino Acid 18	Amino Acid 19	Amino Acid 20	Amino Acid 21	Amino Acid 22
Human	414L	443F	480A	481L	511T	515H	518L	521L	522Q	546F	549E
Western lowland gorilla	414L	443F	480A	481L	511T	515H	518L	521L	522Q	546F	549E
Green monkey	414L	443F	480A	481L	511T	515H	518L	521L	522Q	546F	549E
Rhesus monkey	414L	443F	480A	481L	511T	515H	518L	521L	522Q	546F	549E
White-tufted-ear marmoset	414L	443F	480A	481L	511T	515H	518L	521L	522Q	546F	549E
Pig	415L	444F	481A	482L	512T	516H	519L	522L	523Q	547F	550E
Chinese tree shrew	415L	444F	481A	482L	512T	516H	519L	522L	523Q	547F	550E
American mink	415L	444F	481A	482L	512T	516H	519L	522L	523Q	547F	550E
Eurasian badger	415L	444F	481A	482L	512T	516H	519L	522L	523Q	547F	550E
Egyptian rousette	415L	444F	481A	482L	512T	516H	519L	522L	523Q	547F	550E
Domestic cat	415L	444F	481A	482L	512T	516H	519L	522L	523Q	547F	550E
Lion	415L	444F	481A	482L	512T	516H	519L	522L	523Q	547F	550E
Tiger	415L	444F	481A	482L	512T	516H	519L	522L	523Q	547F	550E
Puma	415L	444F	481A	482L	512T	516H	519L	522L	523Q	547F	550E
Canada lynx	415L	444F	481A	482L	512T	516H	519L	522L	523Q	547F	550E
Fishing cat	415L	444F	481A	482L	512T	516H	519L	522L	523Q	547F	550E
Domestic ferret	415L	444F	481A	482L	512T	516H	519L	522L	523Q	547F	550E
Bobcat	415L	444F	481A	482L	512T	516H	519L	522L	523Q	547F	550E
Raccoon dog	415L	444F	481A	482L	512T	516H	519L	522L	523Q	547F	550E
Dog	415L	444F	481A	482L	512T	516H	519L	522L	523Q	547F	550E
Big brown bat	417L	446F	483A	484L	514T	518H	521L	524L	525Q	549F	552E
Gray squirrel	415L	444F	481A	482L	512T	516H	519L	522L	523Q	547F	550E
Malayan pangolin	416L	445F	482A	483L	513T	517H	520L	523L	524Q	548F	551E
Olive baboon	414L	443F	480A	481L	511T	515H	518L	521L	522Q	546F	549E
Cattle	415L	444F	481A	482L	512T	516H	519L	522L	523Q	547F	550E
White-tailed deer	415L	444F	481A	482L	512T	516H	519L	522L	523Q	547F	550E
Prairie deer mouse	418L	447F	484A	485L	515T	519H	522L	525L	526Q	550F	553E
Norway rat	416L	445F	482A	483L	513T	517H	520L	523L	524Q	548F	551E
Golden hamster	417L	446F	483A	484L	514T	518H	521L	524L	525Q	549F	552E
African clawed frog	382L	411F	448A	449L	479T	483H	486L	489L	490Q	514F	517E
Chinese alligator	307L	336F	373A	374L	404T	408H	411L	414L	415Q	439F	442E
Rock pigeon	321L	350F	387A	388L	418T	422H	425L	428L	429Q	453F	456E
Fathead minnow	385L	414F	451A	452L	482T	486H	489L	492L	493Q	517F	520E

Common Name	Amino Acid 23	Amino Acid 24	Amino Acid 25	Amino Acid 26	Amino Acid 27	Amino Acid 28	Amino Acid 29	Amino Acid 30	Amino Acid 31	Amino Acid 32	Amino Acid 33
Human	550T	553T	557Q	559G	561M	579M	583H	587L	590A	591A	598A
Western lowland gorilla	550T	553T	557Q	559G	561M	579M	583H	587L	590A	591A	598A
Green monkey	550T	553T	557Q	559G	561M	579M	583H	587L	590A	591A	598A
Rhesus monkey	550T	553T	557Q	559G	561M	579M	583H	587L	590A	591A	598A
White-tufted-ear marmoset	550T	553T	557Q	559G	561M	579M	583H	587L	590A	591A	598A
Pig	551T	554T	558Q	560G	562M	580M	584H	588L	591A	592A	599A
Chinese tree shrew	551T	554T	558Q	560G	562M	580M	584H	588L	591A	592A	599A
American mink	551T	554T	558Q	560G	562M	580M	584H	588L	591A	592A	599A
Eurasian badger	551T	554T	558Q	560G	562M	580M	584H	588L	591A	592A	599A
Egyptian rousette	551T	554T	558Q	560G	562M	580M	584H	588L	591A	592A	599A
Domestic cat	551T	554T	558Q	560G	562M	580M	584H	588L	591A	592A	599A
Lion	551T	554T	558Q	560G	562M	580M	584H	588L	591A	592A	599A
Tiger	551T	554T	558Q	560G	562M	580M	584H	588L	591A	592A	599A
Puma	551T	554T	558Q	560G	562M	580M	584H	588L	591A	592A	599A
Canada lynx	551T	554T	558Q	560G	562M	580M	584H	588L	591A	592A	599A
Fishing cat	551T	554T	558Q	560G	562M	580M	584H	588L	591A	592A	599A
Domestic ferret	551T	554T	558Q	560G	562M	580M	584H	588L	591A	592A	599A
Bobcat	551T	554T	558Q	560G	562M	580M	584H	588L	591A	592A	599A
Raccoon dog	551T	554T	558Q	560G	562M	580M	584H	588L	591A	592A	599A
Dog	551T	554T	558Q	560G	562M	580M	584H	588L	591A	592A	599A
Big brown bat	553T	556T	560Q	562G	564M	582M	586H	590L	593A	594A	601A
Gray squirrel	551T	554T	558Q	560G	562M	580M	584H	588L	591A	592A	599A
Malayan pangolin	552T	555T	559Q	561G	563M	581T	585H	589L	592A	593A	600A
Olive baboon	550T	553T	557Q	559G	561M	579M	583H	587L	590V	591T	598C
Cattle	551T	554T	558Q	560G	562M	580M	584H	588L	591A	592A	599A
White-tailed deer	551T	554T	558Q	560G	562M	580M	584H	588L	591A	592A	599A
Prairie deer mouse	554T	557T	561Q	563G	565M	583M	587H	591L	594A	595A	602A
Norway rat	552T	555T	559Q	561G	563M	581M	585H	589L	592A	593A	600A
Golden hamster	553T	556T	560Q	562G	564M	582M	586H	590L	593A	594A	601A
African clawed frog	518T	521T	525Q	527G	529L	547M	551H	555L	558A	559A	566A
Chinese alligator	443T	446T	450Q	452G	454L	472M	476H	480L	483A	484A	491A
Rock pigeon	457T	460T	464Q	466G	468L	486M	490H	494L	497A	498A	505A
Fathead minnow	521T	524T	528Q	530G	532L	550M	554H	558L	561A	562A	569A
